# Supplementary figures and images for: Analysis on single nucleotide polymorphisms of the PeTPS-(-)Apin gene in Pinus elliottii
Source: PLoS One. 2022 May 27;17(5):e0266503. doi: 10.1371/journal.pone.0266503 (PMC9140247; doi:10.1371/journal.pone.0266503)

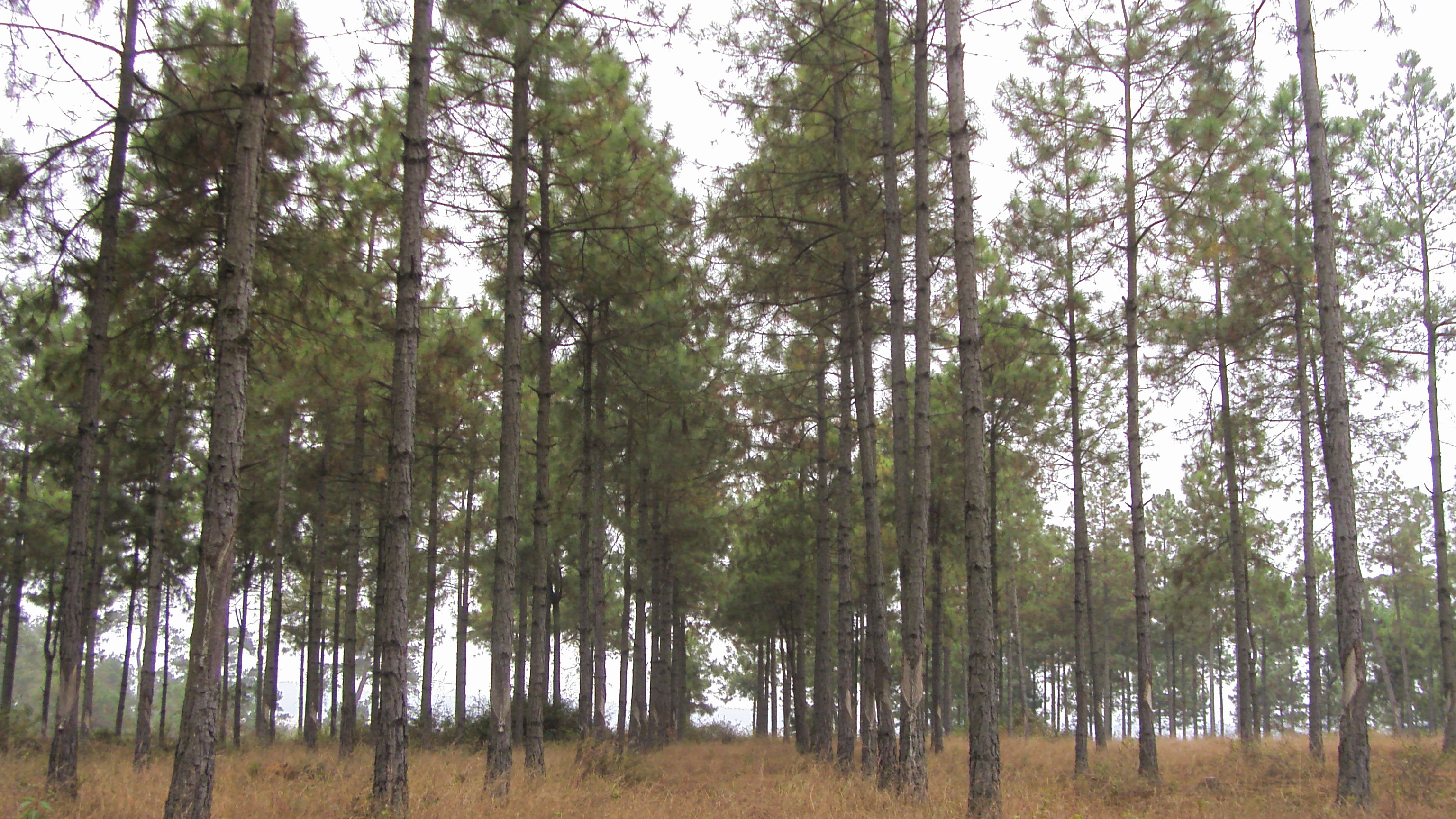

Supplement: S1 Fig — (JPG) [file pone.0266503.s001.jpg]

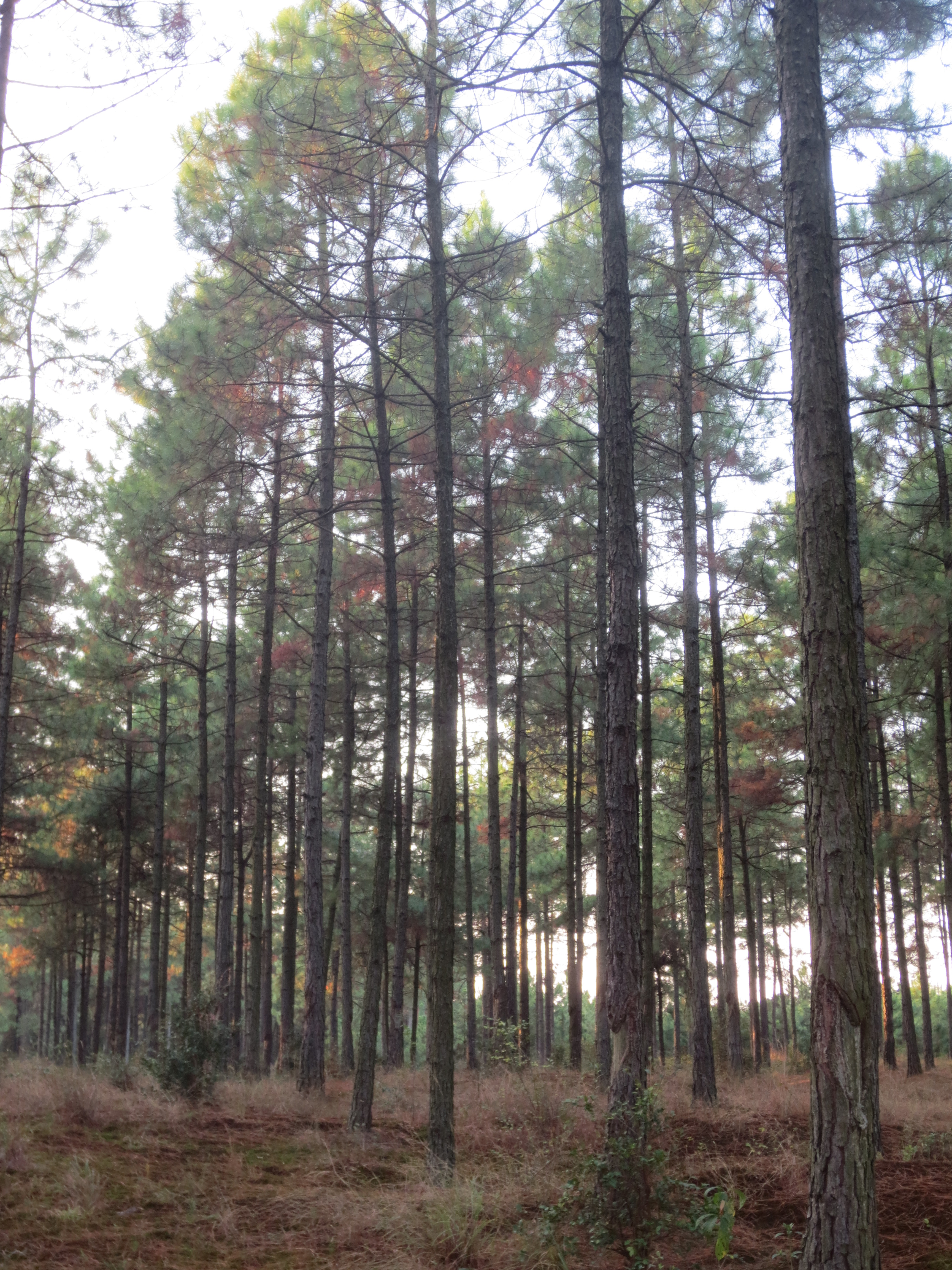

Supplement: S2 Fig — (JPG) [file pone.0266503.s002.jpg]
