# Supplementary material for: Analysis on single nucleotide polymorphisms of the PeTPS-(-)Apin gene in Pinus elliottii
Source: PLoS One. 2022 May 27;17(5):e0266503. doi: 10.1371/journal.pone.0266503 (PMC9140247; doi:10.1371/journal.pone.0266503)
Supplement: S1 Text — (DOCX) [file pone.0266503.s003.docx]

**Determination method for resin traits**

### Determination of resin production capacity

The total annual resin yield per individual (*W*) of sample trees was measured using the method of bark streak-wounding for resin-tapping from June to October, 2016. The outer bark at a height of 1.0 - 1.5 m was incised in a streak with a 45-degree side gutter, 5 mm deep and 2 mm wide, with a sharp sickle, and the tapping load rates maintained at 40 - 45 %. The resin was then collected with a special bag fixed at the base of the wounds. The streak was re-wounded every second day. Moreover, the collected resin was weighted once a month [3].

Basic resin-producing capacity (*W_0_*) was calculated using formula (1), and potential resin-producing-capacity (*W_P_*) was calculated using formula (2). The results were calculated and recorded in Microsoft Office Excel.

$W_{0}=W/L$ (1)

$W_{P}=W_{0}/\pi D$ (2)

*W* is the measured annual total resin yield, *L* the cutting surface length, *D* the DBH, *W_0_* the basic resin production capacity, and *W_P_* the potential resin production capacity.

### Determination of turpentine composition

**Resin sampling**

Each sample tree was sampled in July, September, and November, 2016. Firstly, a hole 6 cm deep and 16 mm wide was drilled into the trunk with a power auger. Then, a resin collector (microcentrifuge tube with a bore diameter of 18 mm) was placed at the trunk drill-hole approximately 1.8 m away from the ground to collect the resin. After 24 h, the collector tube was full of resin and then removed from the sample trees. The resin was then immediately sealed, labeled, and taken to the laboratory. This process isolated the resin sample from the external environment aimed to effectively reduce oxidation and prevent contamination. To avoid volatilization, the chemical constituents were promptly examined.

**Composition determination**

A small amount of turpentine was mixed in the test tube, dissolved in anhydrous ethanol, dropped into phenolphthalein, titrated with tetramethyl-ammonium hydroxide [(CH_3_)_4_NOH·5H_2_O] to a reddish color, and then qualitatively and quantitatively analyzed using gas chromatography (GC) and mass spectrometry (MS)[24]. GC was carried out using the following parameters: 60 ℃ for 2 min, 5 ℃·min^-1^ to 80 ℃, 30 ℃·min^-1^ to 230 ℃, finally 5℃·min^-1^ to 260 ℃, for 10 min. Injection volume: 0.4 μL. GC-MS was carried out using the following parameters: 60 ℃ for 2 min, 5 ℃·min^-1^ to 80 ℃, 30 ℃·min^-1^ to 230 ℃, finally 5℃·min^-1^ to 260 ℃, for 10 min. Injection volume: 0.1 μL.

**Qualitative and quantitative analyses**

Using a chemical workstation data processing system, each component was separately retrieved from an NIST08 spectral library. Using chromatographic peak area normalization methods with Excel statistical tools, the contents of the main ingredients were determined. The content of each chemical component was the average of three measurements.
