# Supplementary material for: Analysis on single nucleotide polymorphisms of the PeTPS-(-)Apin gene in Pinus elliottii
Source: PLoS One. 2022 May 27;17(5):e0266503. doi: 10.1371/journal.pone.0266503 (PMC9140247; doi:10.1371/journal.pone.0266503)
Supplement: S2 Text — (DOCX) [file pone.0266503.s004.docx]

**Population structure analysis**

**method and results**

**Population structure analysis**

To further investigate the population structure, 120 pairs of simple sequence repeat (SSR) primers were designed from more than 1,000 Unigene sequences that were selected from a slash pine trunk transcriptome database [23]. Capillary electrophoresis (CE) of PCR products using these primers were performed by Shanghai Sangon, and sequences were obtained using Genemapper 3.2 (https:// genemapper-id.software.informer.com/) and then imported to Microsoft Office Excel in a 0, 1 format, and then manually arranged into the format required by the software for analysis in the subsequent data analysis. Based on the electrophoretic patterns, a multi-locus genotype analysis software, Structure (http//pritch.bsd.uchicago.edu/structure.html), was used to evaluate population structure and genetic relationships, with the selected parameters: "Number of MCMC Reps" and "burn-in period", "length of burn-in period" set to 10,000, "Number of MCMC Reps after Burnin" set to 10,000, K set to 2~7, repeated 10 times. The optimal value of K was determined using the ΔK method with the online program (Structure Harvester:<http://taylor0.biology.ucla.edu/struct_harvest/>). The Q-matrix of population structure and K-matrix of related relationship were only used for association analysis in MLM model.

**Results of** **group structure and kinship**

We analyzed the population structure of 110 samples of *P. elliottii* using 120 SSR markers, K values set from 2 to 7, and the highest peak of ΔK appeared when K = 4 (Fig.1). In addition, 110 samples were divided into four subgroups corresponding to genetic clusters, which we labeled as red, blue, green, and yellow (Fig 2). Of these, the yellow subgroup shared only few genes with the other subgroups, whereas the red, blue, and green subgroups clearly overlapped. The population genetic structure of the samples was independent of the source of the samples, but there were some differences between sources. Fig 2 shows a Bayesian distribution where vertical bars represent individual samples, and the area of different colors illustrates the proportion of each subpopulation based on the SSR markers (Fig 3). It shows that approximately 80 % of individuals from Ji’an were assigned to the red subgroup, and 60-80 % from Mississippi and Florida to the blue and yellow subgroups, respectively. In individuals from Georgia, none were assigned to the red subgroup and more than 90 % were assigned to the green and blue subgroups.

When the proportion of a single color exceeded 40 % (Q≥0.4), we classified the individual into the subpopulation of that color, but the maximum Q value of sample No. 59 was less than 0.4, so no statistics were collected (Table 1). A total of 35, 35 and 39 samples generated Q < 0.6, 0.6 ≤ Q < 0.8, and Q ≥ 0.8, respectively. This indicated that the genetic relationship of the test group was relatively intricate. The gene introgression of the four subgroups was apparently significant, especially in the green subgroup, in which 59.57 % of the individual Q value was less than 60 %. In addition, the genetic structure was also complex and contained a considerable genetic background of the other subgroups (Fig 2). These results indicate that the 110 samples had a high genetic diversity.


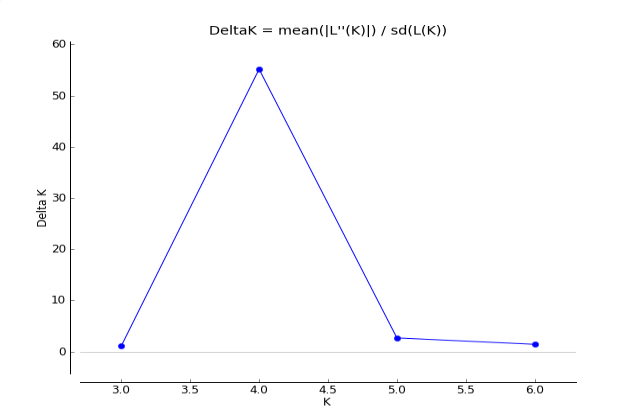


**Fig1. Optimal K value of the population structure.**


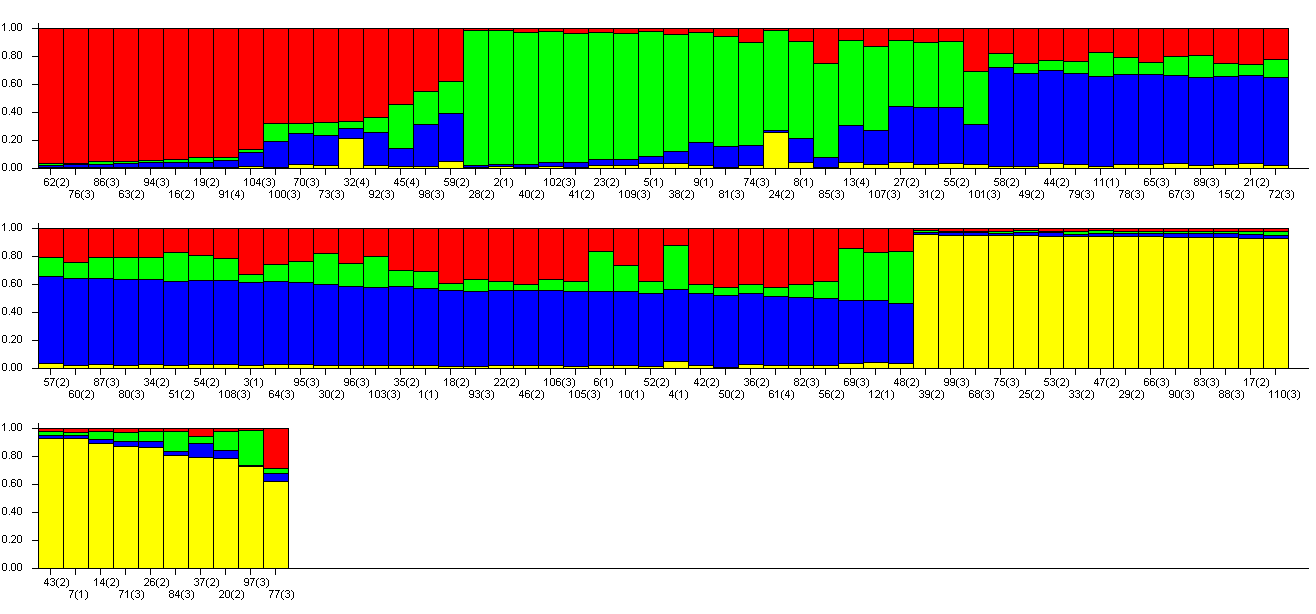


**Fig. 2 Bayesian distribution of the population structure of 110 samples of *P. elliottii*.** Vertical bars represent individual samples. Samples are numbered, with the number in brackets indicating provenance. The area of different colors illustrates the proportion of each subpopulation based on SSR markers.


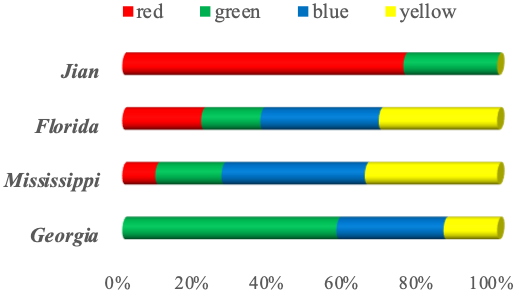


**Fig. 3 Percentage diagram of different provenance clusters.**

**Table 1 Q value distribution in the four subgroups**

| **Group** | **Number** | **Amount** | | |
| --- | --- | --- | --- | --- |
|  |  | **Q < 0.6** | **0.6 ≤ Q < 0.8** | **Q ≥ 0.8** |
| **Red** | 16 | 2 | 5 | 9 |
| **Blue** | 21 | 5 | 7 | 9 |
| **Green** | 47 | 28 | 19 | 0 |
| **Yellow** | 25 | 0 | 4 | 21 |
| **Total** |  | 35 | 35 | 39 |
