# Supplementary material for: Analysis on single nucleotide polymorphisms of the PeTPS-(-)Apin gene in Pinus elliottii
Source: PLoS One. 2022 May 27;17(5):e0266503. doi: 10.1371/journal.pone.0266503 (PMC9140247; doi:10.1371/journal.pone.0266503)
Supplement: S3 Text — (DOCX) [file pone.0266503.s005.docx]

**Full length sequence of *PeTPS-(-)Apin* gene**

> *PeTPS-(-)Apin*

CGGGGGCATTTTTTTACGAAGATTTTTTTTTAACATACTTGTATCTATGTACATGAAAAA

ATACAAACACAAATTTAATATCTTGTATATTATTCTATAAAGACCAAAAACTAAATGTAT

ATTCAATTAAATTTATATAATTAAAATAAGTTCTTAATCCAAGTTCACACCTCTTTAAGC

CGGAGTCTAAGACTTTCTTTAATGTGCATGTACCCGCAAAATAACTTTAATACAACAAAA

GGATTATCAACTAAACATGCAAAAACGTCAGGACAAGGAAACGCATCTATGCTGACATCA

AGTACGGAAGGCAGTGCTAAAATTCTAGCAATGGCCCTCAAATCTGTAGCATACATGGGC

AAGAACGTCGGGACAAGGAAAGGAATCTATGCTGATAAACTGAAGTGACGGATGGAAAGC

AGTGCTCGGACAAAACTGGGTATCTAAACTGAAATCAAAGTCTAGCAATGGCCCTCAAAT

CTGTAGCGTACATGGGCAAGAAGCCCTATATATACCACTGGCAGCTTATATTTTCGAGGT

ATCTCATAGGTCATAAGCTCTCATATCACATTATAAGCAAGCAGGGATGTCTCCTGTTTC

TGTGATCTCGTTGCCTTCCGACTTGTGCCTGCCCACATCGTTCATCGACAGGTCTGGTCG

TGAGCTTATACCTCTCCATATAACAATTCCAAATGTCGCAATGTGCAGGCAAGGGAAATT

AATGACACGTGCTTCCATGAGCATGAATTTGAGGACCGCCGTATCTGATGATGCTGTTAT

AAGACGCAGAGGTGATTTCCATTCCAACCTCTGGGACGATGATTTCATACAGTCCCTTTC

CTCGCCTTATGGGGTGAGTATTTAGCTTCAGCTTCAGCTTCAGTTGAATTTATTCTGGTT

TATAGTCGCTCCTAATTCTGGAAATAATTTTTTTCCCTAATTTA

ATGTATTTTAGGGTTTTTGGTGTTGGAATCTGATGAAATTTTTAATGTGAAATCTTGATT

TTTGTTATTTAAGTTAGTTTTTTGATTGTATATATGGATTTAGCACTATGAAAAATCCCA

AGGCACTTTTATTGTGCAAAATTTACTTATCAATTTTGTGATTTCAATGAATTCTTGACA

AATTTTAAAGAAATTAAACCAAAAAAATTTACCTAAAAAATTTCAAAAAAATCACACTAA

AAACAACTCCTTTGATGTATATGGCTCAGGAACCCTCTTATCGGGAAC

GTGCTGAGAGACTGATTGGGGAAGTAAAGAATAGCTTCAATTCAATGTCAAACGAAGATG

GCGAATCAATCACTCCCCTCGATGATCTGATTCAACGCCTTTGGATGGTCGACAGTGTTG

AACGTTTGGGAATCGACAGACATTTCAAAAAAGAGATAAAATCAGCGCTCGATCATGTTT

ACAGGTCAGCCACCGTAGCCTGAACATCAATATTGTTTATTTATGCCTTACAAATGAAAT

ACACATTATTATACCAATAATTTAAAATGGTATGCAGGTATTGGAGCGAAAAAGGCAT

TGGATGTGGGAGAGAGAGTGTTGTTACTGACCTCAACTCAACTGCCTTGGGTCTTCGAAC

CCTTCGTCTGCACGGATACGATGTGTCTGCAGTTATATATTTATATGTTTTTTTCTTAGT

TTCTGAATTTAAATTTTGAATATCATTGTTTACTGATAATACTTTATATGACAGTGATAT

AGTAATTGTCCACTTAACTACCCCAGTGATAATACCTAAATTTCAATCTAGTATAATTCA

GATAAGTAATTTAGCTCTTTTCTCTCTCCAAGATCTGATAACTTTTCATTTTGTTTCAGA

CGTTTTGAATCATTTCAAAAATCAAAGTGGGCAGTTTGCTTGCACTCTGAAGCAGACAGA

GGATCAGATCAGAACTGTACTTAATTTATATCGGGCTTCCCTCATTGCCTTTCCAGGAGA

GAAAGTCATGGACGAGGCTGAAACTTTCTCTGCGAAATATTTGAAAGAAGCCCTGCAAAA

GATTCCAGTCTCCAGTCTTTCACGAGAGGTGATCAGCCATACCTTTTCATTAACGCATCA

TATGACTT

TTGTTGGTCGCATTATGTGTTGTTATATGTGTTCATTGTTTCAGTAATGTGT

TGGTCTGTGTATGCATTGTTATATGTGTTTCATTATTTCAATAATGCGGTTTGCAGATAG

GGGACGTCCTCGAGTATGGTTGGCACACGTATTTGCCACGATTGGAAGCAAGGAATTACA

TCGACGTCTTCGGACAGGACACTGAAAACAGGTATGACGAGTCTCATAAATTCGGGATGT

TTTAA

GATCCTAGACTGGCATCTTTGTCAGATAGTTGGTCGAAAACTTGTCTATAAATTT

TAGTAATAATTTAAAAGGTAATCAAAACTTGTTAAATTTTTGTATTTTGACTTTTTTGTT

ATTGTTCATGATTGCGTGCTAGCATCAAGTTATTTTGACTTTAAATGGAGGATACCCTGT

TTGGCTTTCAGCAAGTCATATATGAAGACCGAGAAACTTCTCGAACTTGCAAAGTTGGAG

TTCAACATCTTTCACGCCTCACAAAAGCGAGAGTTGGAATATCTCGTGAGGTGATGCCAA

TTCTGTTTCTGAATTTTTTTTGTAGAGAAAAACGGATAGTTAGTGGTTCAGTAAAGTAAA

ATCCAAGAAACTTGTATGCACAGATGGTGGAAAGGCTCTGGTTCGCCTCAAATGACCTTT

TGTCGACATCGTCACGTGGAGTACTACACTTTGGCTTCTTGCATTGCGTTTGAGCCTCAA

CATTCTGGATTCAGACTCGGCTTTGCCAAAGCCTGTCATATCATCACGGTTCTTGATGAT

ATGTACGACACCTTCGGAACACTCGACGAGCTCGAACTCTTCACATCTGCAATTAAGAGG

TGTTCATCATGAATCCCAACAATGTAAAAAAACATTTGCTTGATTGTTCAGTAACTAACT

GACTTGACCAATATTTGTGGGTATAAATGAGCAGATGGGATCCGTCGGCGACAGAGTGCC

TTCCAGAATATATGAAAGGAGTTTACATGATAGTTTACAACACTGTAAATGAAATGTCTC

AGGAGGCAGACAAGGCTCAAGGCCGAGACACGCTCAACTATTGTCGACAGGCTGTATGCA

CAATAATATATCACACAAATAATTTTTCGCTGACAACGTTGAATTCGGCACGTATTTCGT

TAGGTAGTGGTTACTGAAATTGCGTGCTTGTACATGTGCAGTGGGAGGAATATATTGATG

CGTATATGCAAGAAGCAAAGTGGATCGCCAGTGGTGAGGTGCCAACATTTGAGGAGTACT

ATGAGAACGGGAAAGTTAGCTCTGGTCATCGCGTATCGGCATTGCAACCCATTCTGACGA

CCGACATCCCCTTTCCTGAGCACGTCCTCAAGGAAGTTGACATTCCATCGAAGCTCAATG

ACTTGGCATCTGCCATTCTTCGATTACGAGGGGATACGCGCTGCTACCAGGTACTCACTC

TAATCAAAACCATCGGCAACAAATTTGTATCGAATTTGCCATATATTTTAAAACTGTTTT

GCTTTGGAATCATATTGGTATTGGGAATGCAGGCGGACAGGGCCCGTGGAGAAGAAGCTT

CGTGTATATCTTGTTATATGAAAGACAATCCTGGAACAACAGAGGAAGATGCTCTCAATC

ATCTCAACGCCATGATCAGTGATGTAATTAAAGGATTAAATTGGGAGCTTCTCAAACCAA

ACAGCAGCGTTCCCATATCTGCCAAAAAACATGCTTTTGACATTAGCAGAGCTTTTCATT

GTGGCTACAAATATCGAGATGGCTACAGCGTTGCCAACATTGAAACAAGGAGTTTGGTGA

AGAGAACCGTCATTGATCCTGTCACTTTATAAACGCTTTTACACCGTTTAGCCTTTCGCC

CATCTTAAGCGGTCGTTGGATGTTAGTTACAATAAATAAAGTTATTCTATTTGAAGAGGT

GTATATGTATCCAGTGGCCACTTTGATGATAAGTATATTATTAAACTAAATTATTGAAAA

TTCTGTTTATTTATTTTTGATGTGTTCTTAATATTATATTAATTTTTTAGGAAAGGC

TTAGCCTATCAAAATTAGCCCCAAGGAATTGTCGAGAGGTTAAAA

GTGGTTGGAATTTCATAAAAAAAATTCAAAAAATCTTGCTAGTGGTTTCTGAATAATG

TTTTTGATGGTGGAAATGGGTTGGGCACCATCAAAAACCTTTGGGGCATCCAAAATCTTG

AAGTTTGAGAGTCGTAACATGGATTTACAAAAATCCCTCTAGAATTTCCAAAATAAAAGT

GAAAAATAAGAATTGGGATCATTCTCTAGCTTCTTCTAGAACTATCTTGGTTTTGCATAA

CATAAAGATCCCATGTTAAGTTGGATCCCAAGTGATATTGATCTAATTTTCGTAGTCAAA

ACTTGGGAGCATGAGGAATCTATAGCCCTCACATAGATGGATTCATTCTCTAGTAACCTA

GAATAAGAAATCCTCTCATAGAGGTTTTGGTTGTATAGCTTGTTATATAAAATATAATGT

TCATCTCATATCGCTCTATGCC
