# Supplementary material for: Analysis on single nucleotide polymorphisms of the PeTPS-(-)Apin gene in Pinus elliottii
Source: PLoS One. 2022 May 27;17(5):e0266503. doi: 10.1371/journal.pone.0266503 (PMC9140247; doi:10.1371/journal.pone.0266503)
Supplement: S4 Text — (DOCX) [file pone.0266503.s006.docx]

DnaSP Ver. 5.10.01 30 - June - 2020 10:53:54

=====================================================================

Linkage Disequilibrium

----------------------

Input Data File: C:\...\SP-QUANCHANG1.fas

Number of sequences: 22 Number of sequences used: 22

Selected region: 1-4592 Number of sites: 4592

Total number of sites (excluding sites with gaps / missing data): 4592

All Polymorphic sites were considered

Number of polymorphic sites analyzed: 72

Number of pairwise comparisons: 2556

Value of ZnS (Kelly 1997): 0.1847

Value of Za (Rozas et al. 2001): 0.4899

Value of ZZ (Rozas et al. 2001): 0.3052

Value of Wall's B: 0.3521

Value of Wall's Q: 0.4444

===== Regression Equation: Y = a + bX (X measured in kb) =====

|D| values: Y = 0.0608 - 0.0127X (2556 points)

|D'| values: Y = 0.9821 - 0.0457X (2556 points)

|D'|* values: Y = 0.9662 - 0.1115X (1110 points)

r^2 values: Y = 0.3318 - 0.1160X (2556 points)

------------------------------------------------------------------------------------

Site1 Site2 Dist D D' R

399 408 9 0.149 1.000 1.000

399 522 123 -0.017 -1.000 -0.149

399 615 216 0.149 1.000 1.000

399 641 242 0.149 1.000 1.000

399 897 498 -0.017 -1.000 -0.149

399 908 509 -0.017 -1.000 -0.149

399 973 574 -0.017 -1.000 -0.149

399 975 576 -0.017 -1.000 -0.149

399 976 577 -0.017 -1.000 -0.149

399 988 589 -0.017 -1.000 -0.149

399 993 594 -0.008 -1.000 -0.103

399 997 598 -0.017 -1.000 -0.149

399 1017 618 -0.017 -1.000 -0.149

399 1040 641 -0.017 -1.000 -0.149

399 1067 668 -0.017 -1.000 -0.149

399 1091 692 -0.017 -1.000 -0.149

399 1099 700 -0.017 -1.000 -0.149

399 1121 722 -0.017 -1.000 -0.149

399 1122 723 -0.017 -1.000 -0.149

399 1132 733 -0.017 -1.000 -0.149

399 1158 759 -0.008 -1.000 -0.103

399 1219 820 -0.017 -1.000 -0.149

399 1222 823 -0.008 -1.000 -0.103

399 1254 855 0.140 1.000 0.869

399 1277 878 -0.017 -1.000 -0.149

399 1294 895 -0.008 -1.000 -0.103

399 1309 910 -0.017 -1.000 -0.149

399 1412 1013 -0.033 -1.000 -0.222

399 1486 1087 0.107 1.000 0.567

399 1490 1091 -0.037 -0.450 -0.194

399 1499 1100 0.116 1.000 0.624

399 1510 1111 -0.017 -1.000 -0.149

399 1602 1203 0.070 0.607 0.379

399 1623 1224 -0.008 -1.000 -0.103

399 1786 1387 -0.037 -0.450 -0.194

399 1804 1405 0.070 0.607 0.379

399 1807 1408 0.062 0.577 0.327

399 1963 1564 0.062 0.577 0.327

399 1974 1575 0.070 0.607 0.379

399 2027 1628 -0.008 -1.000 -0.103

399 2122 1723 0.062 0.577 0.327

399 2340 1941 -0.008 -1.000 -0.103

399 2344 1945 -0.008 -1.000 -0.103

399 2370 1971 0.021 0.185 0.156

399 2371 1972 -0.012 -0.214 -0.069

399 2400 2001 -0.012 -0.214 -0.069

399 2453 2054 -0.012 -0.214 -0.069

399 2473 2074 -0.008 -1.000 -0.103

399 2493 2094 -0.008 -1.000 -0.103

399 2570 2171 -0.008 -1.000 -0.103

399 2598 2199 -0.012 -0.214 -0.069

399 2608 2209 -0.012 -0.214 -0.069

399 2613 2214 -0.012 -0.214 -0.069

399 2688 2289 -0.004 -0.083 -0.024

399 2889 2490 -0.008 -1.000 -0.103

399 2890 2491 -0.008 -1.000 -0.103

399 2913 2514 -0.012 -0.214 -0.069

399 2943 2544 -0.012 -0.214 -0.069

399 3384 2985 -0.004 -0.083 -0.024

399 3553 3154 0.004 0.029 0.026

399 3605 3206 0.070 0.607 0.379

399 3806 3407 -0.025 -1.000 -0.187

399 3859 3460 -0.004 -0.083 -0.024

399 3935 3536 0.004 0.029 0.026

399 4121 3722 0.004 0.029 0.026

399 4129 3730 0.021 0.185 0.156

399 4137 3738 0.004 0.029 0.026

399 4139 3740 0.054 0.542 0.280

399 4248 3849 0.004 0.029 0.026

399 4283 3884 -0.017 -1.000 -0.149

399 4400 4001 -0.017 -1.000 -0.149

408 522 114 -0.017 -1.000 -0.149

408 615 207 0.149 1.000 1.000

408 641 233 0.149 1.000 1.000

408 897 489 -0.017 -1.000 -0.149

408 908 500 -0.017 -1.000 -0.149

408 973 565 -0.017 -1.000 -0.149

408 975 567 -0.017 -1.000 -0.149

408 976 568 -0.017 -1.000 -0.149

408 988 580 -0.017 -1.000 -0.149

408 993 585 -0.008 -1.000 -0.103

408 997 589 -0.017 -1.000 -0.149

408 1017 609 -0.017 -1.000 -0.149

408 1040 632 -0.017 -1.000 -0.149

408 1067 659 -0.017 -1.000 -0.149

408 1091 683 -0.017 -1.000 -0.149

408 1099 691 -0.017 -1.000 -0.149

408 1121 713 -0.017 -1.000 -0.149

408 1122 714 -0.017 -1.000 -0.149

408 1132 724 -0.017 -1.000 -0.149

408 1158 750 -0.008 -1.000 -0.103

408 1219 811 -0.017 -1.000 -0.149

408 1222 814 -0.008 -1.000 -0.103

408 1254 846 0.140 1.000 0.869

408 1277 869 -0.017 -1.000 -0.149

408 1294 886 -0.008 -1.000 -0.103

408 1309 901 -0.017 -1.000 -0.149

408 1412 1004 -0.033 -1.000 -0.222

408 1486 1078 0.107 1.000 0.567

408 1490 1082 -0.037 -0.450 -0.194

408 1499 1091 0.116 1.000 0.624

408 1510 1102 -0.017 -1.000 -0.149

408 1602 1194 0.070 0.607 0.379

408 1623 1215 -0.008 -1.000 -0.103

408 1786 1378 -0.037 -0.450 -0.194

408 1804 1396 0.070 0.607 0.379

408 1807 1399 0.062 0.577 0.327

408 1963 1555 0.062 0.577 0.327

408 1974 1566 0.070 0.607 0.379

408 2027 1619 -0.008 -1.000 -0.103

408 2122 1714 0.062 0.577 0.327

408 2340 1932 -0.008 -1.000 -0.103

408 2344 1936 -0.008 -1.000 -0.103

408 2370 1962 0.021 0.185 0.156

408 2371 1963 -0.012 -0.214 -0.069

408 2400 1992 -0.012 -0.214 -0.069

408 2453 2045 -0.012 -0.214 -0.069

408 2473 2065 -0.008 -1.000 -0.103

408 2493 2085 -0.008 -1.000 -0.103

408 2570 2162 -0.008 -1.000 -0.103

408 2598 2190 -0.012 -0.214 -0.069

408 2608 2200 -0.012 -0.214 -0.069

408 2613 2205 -0.012 -0.214 -0.069

408 2688 2280 -0.004 -0.083 -0.024

408 2889 2481 -0.008 -1.000 -0.103

408 2890 2482 -0.008 -1.000 -0.103

408 2913 2505 -0.012 -0.214 -0.069

408 2943 2535 -0.012 -0.214 -0.069

408 3384 2976 -0.004 -0.083 -0.024

408 3553 3145 0.004 0.029 0.026

408 3605 3197 0.070 0.607 0.379

408 3806 3398 -0.025 -1.000 -0.187

408 3859 3451 -0.004 -0.083 -0.024

408 3935 3527 0.004 0.029 0.026

408 4121 3713 0.004 0.029 0.026

408 4129 3721 0.021 0.185 0.156

408 4137 3729 0.004 0.029 0.026

408 4139 3731 0.054 0.542 0.280

408 4248 3840 0.004 0.029 0.026

408 4283 3875 -0.017 -1.000 -0.149

408 4400 3992 -0.017 -1.000 -0.149

522 615 93 -0.017 -1.000 -0.149

522 641 119 -0.017 -1.000 -0.149

522 897 375 -0.008 -1.000 -0.100

522 908 386 -0.008 -1.000 -0.100

522 973 451 -0.008 -1.000 -0.100

522 975 453 -0.008 -1.000 -0.100

522 976 454 -0.008 -1.000 -0.100

522 988 466 -0.008 -1.000 -0.100

522 993 471 0.041 1.000 0.690

522 997 475 -0.008 -1.000 -0.100

522 1017 495 -0.008 -1.000 -0.100

522 1040 518 -0.008 -1.000 -0.100

522 1067 545 -0.008 -1.000 -0.100

522 1091 569 -0.008 -1.000 -0.100

522 1099 577 -0.008 -1.000 -0.100

522 1121 599 -0.008 -1.000 -0.100

522 1122 600 -0.008 -1.000 -0.100

522 1132 610 -0.008 -1.000 -0.100

522 1158 636 -0.004 -1.000 -0.069

522 1219 697 -0.008 -1.000 -0.100

522 1222 700 -0.004 -1.000 -0.069

522 1254 732 -0.021 -1.000 -0.171

522 1277 755 -0.008 -1.000 -0.100

522 1294 772 -0.004 -1.000 -0.069

522 1309 787 -0.008 -1.000 -0.100

522 1412 890 -0.017 -1.000 -0.149

522 1486 964 -0.037 -1.000 -0.263

522 1490 968 0.050 1.000 0.346

522 1499 977 -0.033 -1.000 -0.239

522 1510 988 -0.008 -1.000 -0.100

522 1602 1080 -0.033 -1.000 -0.239

522 1623 1101 -0.004 -1.000 -0.069

522 1786 1264 0.050 1.000 0.346

522 1804 1282 -0.033 -1.000 -0.239

522 1807 1285 -0.037 -1.000 -0.263

522 1963 1441 -0.037 -1.000 -0.263

522 1974 1452 -0.033 -1.000 -0.239

522 2027 1505 -0.004 -1.000 -0.069

522 2122 1600 -0.037 -1.000 -0.263

522 2340 1818 -0.004 -1.000 -0.069

522 2344 1822 -0.004 -1.000 -0.069

522 2370 1848 -0.012 -1.000 -0.126

522 2371 1849 0.017 0.267 0.123

522 2400 1878 0.017 0.267 0.123

522 2453 1931 0.017 0.267 0.123

522 2473 1951 -0.004 -1.000 -0.069

522 2493 1971 -0.004 -1.000 -0.069

522 2570 2048 -0.004 -1.000 -0.069

522 2598 2076 0.017 0.267 0.123

522 2608 2086 0.017 0.267 0.123

522 2613 2091 0.017 0.267 0.123

522 2688 2166 0.021 0.313 0.161

522 2889 2367 -0.004 -1.000 -0.069

522 2890 2368 -0.004 -1.000 -0.069

522 2913 2391 0.017 0.267 0.123

522 2943 2421 0.017 0.267 0.123

522 3384 2862 -0.025 -1.000 -0.194

522 3553 3031 -0.021 -1.000 -0.171

522 3605 3083 -0.033 -1.000 -0.239

522 3806 3284 -0.012 -1.000 -0.126

522 3859 3337 -0.025 -1.000 -0.194

522 3935 3413 -0.021 -1.000 -0.171

522 4121 3599 -0.021 -1.000 -0.171

522 4129 3607 -0.012 -1.000 -0.126

522 4137 3615 -0.021 -1.000 -0.171

522 4139 3617 -0.041 -1.000 -0.289

522 4248 3726 -0.021 -1.000 -0.171

522 4283 3761 0.037 0.450 0.450

522 4400 3878 -0.008 -1.000 -0.100

615 641 26 0.149 1.000 1.000

615 897 282 -0.017 -1.000 -0.149

615 908 293 -0.017 -1.000 -0.149

615 973 358 -0.017 -1.000 -0.149

615 975 360 -0.017 -1.000 -0.149

615 976 361 -0.017 -1.000 -0.149

615 988 373 -0.017 -1.000 -0.149

615 993 378 -0.008 -1.000 -0.103

615 997 382 -0.017 -1.000 -0.149

615 1017 402 -0.017 -1.000 -0.149

615 1040 425 -0.017 -1.000 -0.149

615 1067 452 -0.017 -1.000 -0.149

615 1091 476 -0.017 -1.000 -0.149

615 1099 484 -0.017 -1.000 -0.149

615 1121 506 -0.017 -1.000 -0.149

615 1122 507 -0.017 -1.000 -0.149

615 1132 517 -0.017 -1.000 -0.149

615 1158 543 -0.008 -1.000 -0.103

615 1219 604 -0.017 -1.000 -0.149

615 1222 607 -0.008 -1.000 -0.103

615 1254 639 0.140 1.000 0.869

615 1277 662 -0.017 -1.000 -0.149

615 1294 679 -0.008 -1.000 -0.103

615 1309 694 -0.017 -1.000 -0.149

615 1412 797 -0.033 -1.000 -0.222

615 1486 871 0.107 1.000 0.567

615 1490 875 -0.037 -0.450 -0.194

615 1499 884 0.116 1.000 0.624

615 1510 895 -0.017 -1.000 -0.149

615 1602 987 0.070 0.607 0.379

615 1623 1008 -0.008 -1.000 -0.103

615 1786 1171 -0.037 -0.450 -0.194

615 1804 1189 0.070 0.607 0.379

615 1807 1192 0.062 0.577 0.327

615 1963 1348 0.062 0.577 0.327

615 1974 1359 0.070 0.607 0.379

615 2027 1412 -0.008 -1.000 -0.103

615 2122 1507 0.062 0.577 0.327

615 2340 1725 -0.008 -1.000 -0.103

615 2344 1729 -0.008 -1.000 -0.103

615 2370 1755 0.021 0.185 0.156

615 2371 1756 -0.012 -0.214 -0.069

615 2400 1785 -0.012 -0.214 -0.069

615 2453 1838 -0.012 -0.214 -0.069

615 2473 1858 -0.008 -1.000 -0.103

615 2493 1878 -0.008 -1.000 -0.103

615 2570 1955 -0.008 -1.000 -0.103

615 2598 1983 -0.012 -0.214 -0.069

615 2608 1993 -0.012 -0.214 -0.069

615 2613 1998 -0.012 -0.214 -0.069

615 2688 2073 -0.004 -0.083 -0.024

615 2889 2274 -0.008 -1.000 -0.103

615 2890 2275 -0.008 -1.000 -0.103

615 2913 2298 -0.012 -0.214 -0.069

615 2943 2328 -0.012 -0.214 -0.069

615 3384 2769 -0.004 -0.083 -0.024

615 3553 2938 0.004 0.029 0.026

615 3605 2990 0.070 0.607 0.379

615 3806 3191 -0.025 -1.000 -0.187

615 3859 3244 -0.004 -0.083 -0.024

615 3935 3320 0.004 0.029 0.026

615 4121 3506 0.004 0.029 0.026

615 4129 3514 0.021 0.185 0.156

615 4137 3522 0.004 0.029 0.026

615 4139 3524 0.054 0.542 0.280

615 4248 3633 0.004 0.029 0.026

615 4283 3668 -0.017 -1.000 -0.149

615 4400 3785 -0.017 -1.000 -0.149

641 897 256 -0.017 -1.000 -0.149

641 908 267 -0.017 -1.000 -0.149

641 973 332 -0.017 -1.000 -0.149

641 975 334 -0.017 -1.000 -0.149

641 976 335 -0.017 -1.000 -0.149

641 988 347 -0.017 -1.000 -0.149

641 993 352 -0.008 -1.000 -0.103

641 997 356 -0.017 -1.000 -0.149

641 1017 376 -0.017 -1.000 -0.149

641 1040 399 -0.017 -1.000 -0.149

641 1067 426 -0.017 -1.000 -0.149

641 1091 450 -0.017 -1.000 -0.149

641 1099 458 -0.017 -1.000 -0.149

641 1121 480 -0.017 -1.000 -0.149

641 1122 481 -0.017 -1.000 -0.149

641 1132 491 -0.017 -1.000 -0.149

641 1158 517 -0.008 -1.000 -0.103

641 1219 578 -0.017 -1.000 -0.149

641 1222 581 -0.008 -1.000 -0.103

641 1254 613 0.140 1.000 0.869

641 1277 636 -0.017 -1.000 -0.149

641 1294 653 -0.008 -1.000 -0.103

641 1309 668 -0.017 -1.000 -0.149

641 1412 771 -0.033 -1.000 -0.222

641 1486 845 0.107 1.000 0.567

641 1490 849 -0.037 -0.450 -0.194

641 1499 858 0.116 1.000 0.624

641 1510 869 -0.017 -1.000 -0.149

641 1602 961 0.070 0.607 0.379

641 1623 982 -0.008 -1.000 -0.103

641 1786 1145 -0.037 -0.450 -0.194

641 1804 1163 0.070 0.607 0.379

641 1807 1166 0.062 0.577 0.327

641 1963 1322 0.062 0.577 0.327

641 1974 1333 0.070 0.607 0.379

641 2027 1386 -0.008 -1.000 -0.103

641 2122 1481 0.062 0.577 0.327

641 2340 1699 -0.008 -1.000 -0.103

641 2344 1703 -0.008 -1.000 -0.103

641 2370 1729 0.021 0.185 0.156

641 2371 1730 -0.012 -0.214 -0.069

641 2400 1759 -0.012 -0.214 -0.069

641 2453 1812 -0.012 -0.214 -0.069

641 2473 1832 -0.008 -1.000 -0.103

641 2493 1852 -0.008 -1.000 -0.103

641 2570 1929 -0.008 -1.000 -0.103

641 2598 1957 -0.012 -0.214 -0.069

641 2608 1967 -0.012 -0.214 -0.069

641 2613 1972 -0.012 -0.214 -0.069

641 2688 2047 -0.004 -0.083 -0.024

641 2889 2248 -0.008 -1.000 -0.103

641 2890 2249 -0.008 -1.000 -0.103

641 2913 2272 -0.012 -0.214 -0.069

641 2943 2302 -0.012 -0.214 -0.069

641 3384 2743 -0.004 -0.083 -0.024

641 3553 2912 0.004 0.029 0.026

641 3605 2964 0.070 0.607 0.379

641 3806 3165 -0.025 -1.000 -0.187

641 3859 3218 -0.004 -0.083 -0.024

641 3935 3294 0.004 0.029 0.026

641 4121 3480 0.004 0.029 0.026

641 4129 3488 0.021 0.185 0.156

641 4137 3496 0.004 0.029 0.026

641 4139 3498 0.054 0.542 0.280

641 4248 3607 0.004 0.029 0.026

641 4283 3642 -0.017 -1.000 -0.149

641 4400 3759 -0.017 -1.000 -0.149

897 908 11 0.083 1.000 1.000

897 973 76 0.083 1.000 1.000

897 975 78 0.083 1.000 1.000

897 976 79 0.083 1.000 1.000

897 988 91 0.083 1.000 1.000

897 993 96 -0.004 -1.000 -0.069

897 997 100 -0.008 -1.000 -0.100

897 1017 120 0.083 1.000 1.000

897 1040 143 0.083 1.000 1.000

897 1067 170 0.083 1.000 1.000

897 1091 194 0.083 1.000 1.000

897 1099 202 0.083 1.000 1.000

897 1121 224 0.083 1.000 1.000

897 1122 225 0.083 1.000 1.000

897 1132 235 0.083 1.000 1.000

897 1158 261 -0.004 -1.000 -0.069

897 1219 322 0.083 1.000 1.000

897 1222 325 0.041 1.000 0.690

897 1254 357 -0.021 -1.000 -0.171

897 1277 380 0.083 1.000 1.000

897 1294 397 -0.004 -1.000 -0.069

897 1309 412 0.083 1.000 1.000

897 1412 515 -0.017 -1.000 -0.149

897 1486 589 0.054 1.000 0.380

897 1490 593 0.050 1.000 0.346

897 1499 602 0.058 1.000 0.418

897 1510 613 0.083 1.000 1.000

897 1602 705 0.058 1.000 0.418

897 1623 726 -0.004 -1.000 -0.069

897 1786 889 -0.041 -1.000 -0.289

897 1804 907 0.058 1.000 0.418

897 1807 910 0.054 1.000 0.380

897 1963 1066 0.054 1.000 0.380

897 1974 1077 0.058 1.000 0.418

897 2027 1130 -0.004 -1.000 -0.069

897 2122 1225 0.054 1.000 0.380

897 2340 1443 -0.004 -1.000 -0.069

897 2344 1447 -0.004 -1.000 -0.069

897 2370 1473 -0.012 -1.000 -0.126

897 2371 1474 -0.029 -1.000 -0.216

897 2400 1503 -0.029 -1.000 -0.216

897 2453 1556 -0.029 -1.000 -0.216

897 2473 1576 -0.004 -1.000 -0.069

897 2493 1596 -0.004 -1.000 -0.069

897 2570 1673 -0.004 -1.000 -0.069

897 2598 1701 -0.029 -1.000 -0.216

897 2608 1711 -0.029 -1.000 -0.216

897 2613 1716 -0.029 -1.000 -0.216

897 2688 1791 -0.025 -1.000 -0.194

897 2889 1992 -0.004 -1.000 -0.069

897 2890 1993 -0.004 -1.000 -0.069

897 2913 2016 -0.029 -1.000 -0.216

897 2943 2046 -0.029 -1.000 -0.216

897 3384 2487 -0.025 -1.000 -0.194

897 3553 2656 -0.021 -1.000 -0.171

897 3605 2708 0.058 1.000 0.418

897 3806 2909 -0.012 -1.000 -0.126

897 3859 2962 -0.025 -1.000 -0.194

897 3935 3038 -0.021 -1.000 -0.171

897 4121 3224 -0.021 -1.000 -0.171

897 4129 3232 -0.012 -1.000 -0.126

897 4137 3240 -0.021 -1.000 -0.171

897 4139 3242 0.050 1.000 0.346

897 4248 3351 -0.021 -1.000 -0.171

897 4283 3386 -0.008 -1.000 -0.100

897 4400 3503 -0.008 -1.000 -0.100

908 973 65 0.083 1.000 1.000

908 975 67 0.083 1.000 1.000

908 976 68 0.083 1.000 1.000

908 988 80 0.083 1.000 1.000

908 993 85 -0.004 -1.000 -0.069

908 997 89 -0.008 -1.000 -0.100

908 1017 109 0.083 1.000 1.000

908 1040 132 0.083 1.000 1.000

908 1067 159 0.083 1.000 1.000

908 1091 183 0.083 1.000 1.000

908 1099 191 0.083 1.000 1.000

908 1121 213 0.083 1.000 1.000

908 1122 214 0.083 1.000 1.000

908 1132 224 0.083 1.000 1.000

908 1158 250 -0.004 -1.000 -0.069

908 1219 311 0.083 1.000 1.000

908 1222 314 0.041 1.000 0.690

908 1254 346 -0.021 -1.000 -0.171

908 1277 369 0.083 1.000 1.000

908 1294 386 -0.004 -1.000 -0.069

908 1309 401 0.083 1.000 1.000

908 1412 504 -0.017 -1.000 -0.149

908 1486 578 0.054 1.000 0.380

908 1490 582 0.050 1.000 0.346

908 1499 591 0.058 1.000 0.418

908 1510 602 0.083 1.000 1.000

908 1602 694 0.058 1.000 0.418

908 1623 715 -0.004 -1.000 -0.069

908 1786 878 -0.041 -1.000 -0.289

908 1804 896 0.058 1.000 0.418

908 1807 899 0.054 1.000 0.380

908 1963 1055 0.054 1.000 0.380

908 1974 1066 0.058 1.000 0.418

908 2027 1119 -0.004 -1.000 -0.069

908 2122 1214 0.054 1.000 0.380

908 2340 1432 -0.004 -1.000 -0.069

908 2344 1436 -0.004 -1.000 -0.069

908 2370 1462 -0.012 -1.000 -0.126

908 2371 1463 -0.029 -1.000 -0.216

908 2400 1492 -0.029 -1.000 -0.216

908 2453 1545 -0.029 -1.000 -0.216

908 2473 1565 -0.004 -1.000 -0.069

908 2493 1585 -0.004 -1.000 -0.069

908 2570 1662 -0.004 -1.000 -0.069

908 2598 1690 -0.029 -1.000 -0.216

908 2608 1700 -0.029 -1.000 -0.216

908 2613 1705 -0.029 -1.000 -0.216

908 2688 1780 -0.025 -1.000 -0.194

908 2889 1981 -0.004 -1.000 -0.069

908 2890 1982 -0.004 -1.000 -0.069

908 2913 2005 -0.029 -1.000 -0.216

908 2943 2035 -0.029 -1.000 -0.216

908 3384 2476 -0.025 -1.000 -0.194

908 3553 2645 -0.021 -1.000 -0.171

908 3605 2697 0.058 1.000 0.418

908 3806 2898 -0.012 -1.000 -0.126

908 3859 2951 -0.025 -1.000 -0.194

908 3935 3027 -0.021 -1.000 -0.171

908 4121 3213 -0.021 -1.000 -0.171

908 4129 3221 -0.012 -1.000 -0.126

908 4137 3229 -0.021 -1.000 -0.171

908 4139 3231 0.050 1.000 0.346

908 4248 3340 -0.021 -1.000 -0.171

908 4283 3375 -0.008 -1.000 -0.100

908 4400 3492 -0.008 -1.000 -0.100

973 975 2 0.083 1.000 1.000

973 976 3 0.083 1.000 1.000

973 988 15 0.083 1.000 1.000

973 993 20 -0.004 -1.000 -0.069

973 997 24 -0.008 -1.000 -0.100

973 1017 44 0.083 1.000 1.000

973 1040 67 0.083 1.000 1.000

973 1067 94 0.083 1.000 1.000

973 1091 118 0.083 1.000 1.000

973 1099 126 0.083 1.000 1.000

973 1121 148 0.083 1.000 1.000

973 1122 149 0.083 1.000 1.000

973 1132 159 0.083 1.000 1.000

973 1158 185 -0.004 -1.000 -0.069

973 1219 246 0.083 1.000 1.000

973 1222 249 0.041 1.000 0.690

973 1254 281 -0.021 -1.000 -0.171

973 1277 304 0.083 1.000 1.000

973 1294 321 -0.004 -1.000 -0.069

973 1309 336 0.083 1.000 1.000

973 1412 439 -0.017 -1.000 -0.149

973 1486 513 0.054 1.000 0.380

973 1490 517 0.050 1.000 0.346

973 1499 526 0.058 1.000 0.418

973 1510 537 0.083 1.000 1.000

973 1602 629 0.058 1.000 0.418

973 1623 650 -0.004 -1.000 -0.069

973 1786 813 -0.041 -1.000 -0.289

973 1804 831 0.058 1.000 0.418

973 1807 834 0.054 1.000 0.380

973 1963 990 0.054 1.000 0.380

973 1974 1001 0.058 1.000 0.418

973 2027 1054 -0.004 -1.000 -0.069

973 2122 1149 0.054 1.000 0.380

973 2340 1367 -0.004 -1.000 -0.069

973 2344 1371 -0.004 -1.000 -0.069

973 2370 1397 -0.012 -1.000 -0.126

973 2371 1398 -0.029 -1.000 -0.216

973 2400 1427 -0.029 -1.000 -0.216

973 2453 1480 -0.029 -1.000 -0.216

973 2473 1500 -0.004 -1.000 -0.069

973 2493 1520 -0.004 -1.000 -0.069

973 2570 1597 -0.004 -1.000 -0.069

973 2598 1625 -0.029 -1.000 -0.216

973 2608 1635 -0.029 -1.000 -0.216

973 2613 1640 -0.029 -1.000 -0.216

973 2688 1715 -0.025 -1.000 -0.194

973 2889 1916 -0.004 -1.000 -0.069

973 2890 1917 -0.004 -1.000 -0.069

973 2913 1940 -0.029 -1.000 -0.216

973 2943 1970 -0.029 -1.000 -0.216

973 3384 2411 -0.025 -1.000 -0.194

973 3553 2580 -0.021 -1.000 -0.171

973 3605 2632 0.058 1.000 0.418

973 3806 2833 -0.012 -1.000 -0.126

973 3859 2886 -0.025 -1.000 -0.194

973 3935 2962 -0.021 -1.000 -0.171

973 4121 3148 -0.021 -1.000 -0.171

973 4129 3156 -0.012 -1.000 -0.126

973 4137 3164 -0.021 -1.000 -0.171

973 4139 3166 0.050 1.000 0.346

973 4248 3275 -0.021 -1.000 -0.171

973 4283 3310 -0.008 -1.000 -0.100

973 4400 3427 -0.008 -1.000 -0.100

975 976 1 0.083 1.000 1.000

975 988 13 0.083 1.000 1.000

975 993 18 -0.004 -1.000 -0.069

975 997 22 -0.008 -1.000 -0.100

975 1017 42 0.083 1.000 1.000

975 1040 65 0.083 1.000 1.000

975 1067 92 0.083 1.000 1.000

975 1091 116 0.083 1.000 1.000

975 1099 124 0.083 1.000 1.000

975 1121 146 0.083 1.000 1.000

975 1122 147 0.083 1.000 1.000

975 1132 157 0.083 1.000 1.000

975 1158 183 -0.004 -1.000 -0.069

975 1219 244 0.083 1.000 1.000

975 1222 247 0.041 1.000 0.690

975 1254 279 -0.021 -1.000 -0.171

975 1277 302 0.083 1.000 1.000

975 1294 319 -0.004 -1.000 -0.069

975 1309 334 0.083 1.000 1.000

975 1412 437 -0.017 -1.000 -0.149

975 1486 511 0.054 1.000 0.380

975 1490 515 0.050 1.000 0.346

975 1499 524 0.058 1.000 0.418

975 1510 535 0.083 1.000 1.000

975 1602 627 0.058 1.000 0.418

975 1623 648 -0.004 -1.000 -0.069

975 1786 811 -0.041 -1.000 -0.289

975 1804 829 0.058 1.000 0.418

975 1807 832 0.054 1.000 0.380

975 1963 988 0.054 1.000 0.380

975 1974 999 0.058 1.000 0.418

975 2027 1052 -0.004 -1.000 -0.069

975 2122 1147 0.054 1.000 0.380

975 2340 1365 -0.004 -1.000 -0.069

975 2344 1369 -0.004 -1.000 -0.069

975 2370 1395 -0.012 -1.000 -0.126

975 2371 1396 -0.029 -1.000 -0.216

975 2400 1425 -0.029 -1.000 -0.216

975 2453 1478 -0.029 -1.000 -0.216

975 2473 1498 -0.004 -1.000 -0.069

975 2493 1518 -0.004 -1.000 -0.069

975 2570 1595 -0.004 -1.000 -0.069

975 2598 1623 -0.029 -1.000 -0.216

975 2608 1633 -0.029 -1.000 -0.216

975 2613 1638 -0.029 -1.000 -0.216

975 2688 1713 -0.025 -1.000 -0.194

975 2889 1914 -0.004 -1.000 -0.069

975 2890 1915 -0.004 -1.000 -0.069

975 2913 1938 -0.029 -1.000 -0.216

975 2943 1968 -0.029 -1.000 -0.216

975 3384 2409 -0.025 -1.000 -0.194

975 3553 2578 -0.021 -1.000 -0.171

975 3605 2630 0.058 1.000 0.418

975 3806 2831 -0.012 -1.000 -0.126

975 3859 2884 -0.025 -1.000 -0.194

975 3935 2960 -0.021 -1.000 -0.171

975 4121 3146 -0.021 -1.000 -0.171

975 4129 3154 -0.012 -1.000 -0.126

975 4137 3162 -0.021 -1.000 -0.171

975 4139 3164 0.050 1.000 0.346

975 4248 3273 -0.021 -1.000 -0.171

975 4283 3308 -0.008 -1.000 -0.100

975 4400 3425 -0.008 -1.000 -0.100

976 988 12 0.083 1.000 1.000

976 993 17 -0.004 -1.000 -0.069

976 997 21 -0.008 -1.000 -0.100

976 1017 41 0.083 1.000 1.000

976 1040 64 0.083 1.000 1.000

976 1067 91 0.083 1.000 1.000

976 1091 115 0.083 1.000 1.000

976 1099 123 0.083 1.000 1.000

976 1121 145 0.083 1.000 1.000

976 1122 146 0.083 1.000 1.000

976 1132 156 0.083 1.000 1.000

976 1158 182 -0.004 -1.000 -0.069

976 1219 243 0.083 1.000 1.000

976 1222 246 0.041 1.000 0.690

976 1254 278 -0.021 -1.000 -0.171

976 1277 301 0.083 1.000 1.000

976 1294 318 -0.004 -1.000 -0.069

976 1309 333 0.083 1.000 1.000

976 1412 436 -0.017 -1.000 -0.149

976 1486 510 0.054 1.000 0.380

976 1490 514 0.050 1.000 0.346

976 1499 523 0.058 1.000 0.418

976 1510 534 0.083 1.000 1.000

976 1602 626 0.058 1.000 0.418

976 1623 647 -0.004 -1.000 -0.069

976 1786 810 -0.041 -1.000 -0.289

976 1804 828 0.058 1.000 0.418

976 1807 831 0.054 1.000 0.380

976 1963 987 0.054 1.000 0.380

976 1974 998 0.058 1.000 0.418

976 2027 1051 -0.004 -1.000 -0.069

976 2122 1146 0.054 1.000 0.380

976 2340 1364 -0.004 -1.000 -0.069

976 2344 1368 -0.004 -1.000 -0.069

976 2370 1394 -0.012 -1.000 -0.126

976 2371 1395 -0.029 -1.000 -0.216

976 2400 1424 -0.029 -1.000 -0.216

976 2453 1477 -0.029 -1.000 -0.216

976 2473 1497 -0.004 -1.000 -0.069

976 2493 1517 -0.004 -1.000 -0.069

976 2570 1594 -0.004 -1.000 -0.069

976 2598 1622 -0.029 -1.000 -0.216

976 2608 1632 -0.029 -1.000 -0.216

976 2613 1637 -0.029 -1.000 -0.216

976 2688 1712 -0.025 -1.000 -0.194

976 2889 1913 -0.004 -1.000 -0.069

976 2890 1914 -0.004 -1.000 -0.069

976 2913 1937 -0.029 -1.000 -0.216

976 2943 1967 -0.029 -1.000 -0.216

976 3384 2408 -0.025 -1.000 -0.194

976 3553 2577 -0.021 -1.000 -0.171

976 3605 2629 0.058 1.000 0.418

976 3806 2830 -0.012 -1.000 -0.126

976 3859 2883 -0.025 -1.000 -0.194

976 3935 2959 -0.021 -1.000 -0.171

976 4121 3145 -0.021 -1.000 -0.171

976 4129 3153 -0.012 -1.000 -0.126

976 4137 3161 -0.021 -1.000 -0.171

976 4139 3163 0.050 1.000 0.346

976 4248 3272 -0.021 -1.000 -0.171

976 4283 3307 -0.008 -1.000 -0.100

976 4400 3424 -0.008 -1.000 -0.100

988 993 5 -0.004 -1.000 -0.069

988 997 9 -0.008 -1.000 -0.100

988 1017 29 0.083 1.000 1.000

988 1040 52 0.083 1.000 1.000

988 1067 79 0.083 1.000 1.000

988 1091 103 0.083 1.000 1.000

988 1099 111 0.083 1.000 1.000

988 1121 133 0.083 1.000 1.000

988 1122 134 0.083 1.000 1.000

988 1132 144 0.083 1.000 1.000

988 1158 170 -0.004 -1.000 -0.069

988 1219 231 0.083 1.000 1.000

988 1222 234 0.041 1.000 0.690

988 1254 266 -0.021 -1.000 -0.171

988 1277 289 0.083 1.000 1.000

988 1294 306 -0.004 -1.000 -0.069

988 1309 321 0.083 1.000 1.000

988 1412 424 -0.017 -1.000 -0.149

988 1486 498 0.054 1.000 0.380

988 1490 502 0.050 1.000 0.346

988 1499 511 0.058 1.000 0.418

988 1510 522 0.083 1.000 1.000

988 1602 614 0.058 1.000 0.418

988 1623 635 -0.004 -1.000 -0.069

988 1786 798 -0.041 -1.000 -0.289

988 1804 816 0.058 1.000 0.418

988 1807 819 0.054 1.000 0.380

988 1963 975 0.054 1.000 0.380

988 1974 986 0.058 1.000 0.418

988 2027 1039 -0.004 -1.000 -0.069

988 2122 1134 0.054 1.000 0.380

988 2340 1352 -0.004 -1.000 -0.069

988 2344 1356 -0.004 -1.000 -0.069

988 2370 1382 -0.012 -1.000 -0.126

988 2371 1383 -0.029 -1.000 -0.216

988 2400 1412 -0.029 -1.000 -0.216

988 2453 1465 -0.029 -1.000 -0.216

988 2473 1485 -0.004 -1.000 -0.069

988 2493 1505 -0.004 -1.000 -0.069

988 2570 1582 -0.004 -1.000 -0.069

988 2598 1610 -0.029 -1.000 -0.216

988 2608 1620 -0.029 -1.000 -0.216

988 2613 1625 -0.029 -1.000 -0.216

988 2688 1700 -0.025 -1.000 -0.194

988 2889 1901 -0.004 -1.000 -0.069

988 2890 1902 -0.004 -1.000 -0.069

988 2913 1925 -0.029 -1.000 -0.216

988 2943 1955 -0.029 -1.000 -0.216

988 3384 2396 -0.025 -1.000 -0.194

988 3553 2565 -0.021 -1.000 -0.171

988 3605 2617 0.058 1.000 0.418

988 3806 2818 -0.012 -1.000 -0.126

988 3859 2871 -0.025 -1.000 -0.194

988 3935 2947 -0.021 -1.000 -0.171

988 4121 3133 -0.021 -1.000 -0.171

988 4129 3141 -0.012 -1.000 -0.126

988 4137 3149 -0.021 -1.000 -0.171

988 4139 3151 0.050 1.000 0.346

988 4248 3260 -0.021 -1.000 -0.171

988 4283 3295 -0.008 -1.000 -0.100

988 4400 3412 -0.008 -1.000 -0.100

993 997 4 -0.004 -1.000 -0.069

993 1017 24 -0.004 -1.000 -0.069

993 1040 47 -0.004 -1.000 -0.069

993 1067 74 -0.004 -1.000 -0.069

993 1091 98 -0.004 -1.000 -0.069

993 1099 106 -0.004 -1.000 -0.069

993 1121 128 -0.004 -1.000 -0.069

993 1122 129 -0.004 -1.000 -0.069

993 1132 139 -0.004 -1.000 -0.069

993 1158 165 -0.002 -1.000 -0.048

993 1219 226 -0.004 -1.000 -0.069

993 1222 229 -0.002 -1.000 -0.048

993 1254 261 -0.010 -1.000 -0.118

993 1277 284 -0.004 -1.000 -0.069

993 1294 301 -0.002 -1.000 -0.048

993 1309 316 -0.004 -1.000 -0.069

993 1412 419 -0.008 -1.000 -0.103

993 1486 493 -0.019 -1.000 -0.182

993 1490 497 0.025 1.000 0.239

993 1499 506 -0.017 -1.000 -0.165

993 1510 517 -0.004 -1.000 -0.069

993 1602 609 -0.017 -1.000 -0.165

993 1623 630 -0.002 -1.000 -0.048

993 1786 793 0.025 1.000 0.239

993 1804 811 -0.017 -1.000 -0.165

993 1807 814 -0.019 -1.000 -0.182

993 1963 970 -0.019 -1.000 -0.182

993 1974 981 -0.017 -1.000 -0.165

993 2027 1034 -0.002 -1.000 -0.048

993 2122 1129 -0.019 -1.000 -0.182

993 2340 1347 -0.002 -1.000 -0.048

993 2344 1351 -0.002 -1.000 -0.048

993 2370 1377 -0.006 -1.000 -0.087

993 2371 1378 -0.014 -1.000 -0.149

993 2400 1407 -0.014 -1.000 -0.149

993 2453 1460 -0.014 -1.000 -0.149

993 2473 1480 -0.002 -1.000 -0.048

993 2493 1500 -0.002 -1.000 -0.048

993 2570 1577 -0.002 -1.000 -0.048

993 2598 1605 -0.014 -1.000 -0.149

993 2608 1615 -0.014 -1.000 -0.149

993 2613 1620 -0.014 -1.000 -0.149

993 2688 1695 -0.012 -1.000 -0.134

993 2889 1896 -0.002 -1.000 -0.048

993 2890 1897 -0.002 -1.000 -0.048

993 2913 1920 -0.014 -1.000 -0.149

993 2943 1950 -0.014 -1.000 -0.149

993 3384 2391 -0.012 -1.000 -0.134

993 3553 2560 -0.010 -1.000 -0.118

993 3605 2612 -0.017 -1.000 -0.165

993 3806 2813 -0.006 -1.000 -0.087

993 3859 2866 -0.012 -1.000 -0.134

993 3935 2942 -0.010 -1.000 -0.118

993 4121 3128 -0.010 -1.000 -0.118

993 4129 3136 -0.006 -1.000 -0.087

993 4137 3144 -0.010 -1.000 -0.118

993 4139 3146 -0.021 -1.000 -0.199

993 4248 3255 -0.010 -1.000 -0.118

993 4283 3290 0.041 1.000 0.690

993 4400 3407 -0.004 -1.000 -0.069

997 1017 20 -0.008 -1.000 -0.100

997 1040 43 -0.008 -1.000 -0.100

997 1067 70 -0.008 -1.000 -0.100

997 1091 94 -0.008 -1.000 -0.100

997 1099 102 -0.008 -1.000 -0.100

997 1121 124 -0.008 -1.000 -0.100

997 1122 125 -0.008 -1.000 -0.100

997 1132 135 -0.008 -1.000 -0.100

997 1158 161 -0.004 -1.000 -0.069

997 1219 222 -0.008 -1.000 -0.100

997 1222 225 -0.004 -1.000 -0.069

997 1254 257 -0.021 -1.000 -0.171

997 1277 280 -0.008 -1.000 -0.100

997 1294 297 -0.004 -1.000 -0.069

997 1309 312 -0.008 -1.000 -0.100

997 1412 415 0.074 1.000 0.671

997 1486 489 -0.037 -1.000 -0.263

997 1490 493 0.004 0.083 0.029

997 1499 502 -0.033 -1.000 -0.239

997 1510 513 -0.008 -1.000 -0.100

997 1602 605 -0.033 -1.000 -0.239

997 1623 626 -0.004 -1.000 -0.069

997 1786 789 0.050 1.000 0.346

997 1804 807 -0.033 -1.000 -0.239

997 1807 810 -0.037 -1.000 -0.263

997 1963 966 -0.037 -1.000 -0.263

997 1974 977 -0.033 -1.000 -0.239

997 2027 1030 -0.004 -1.000 -0.069

997 2122 1125 -0.037 -1.000 -0.263

997 2340 1343 -0.004 -1.000 -0.069

997 2344 1347 -0.004 -1.000 -0.069

997 2370 1373 -0.012 -1.000 -0.126

997 2371 1374 -0.029 -1.000 -0.216

997 2400 1403 -0.029 -1.000 -0.216

997 2453 1456 -0.029 -1.000 -0.216

997 2473 1476 -0.004 -1.000 -0.069

997 2493 1496 -0.004 -1.000 -0.069

997 2570 1573 -0.004 -1.000 -0.069

997 2598 1601 -0.029 -1.000 -0.216

997 2608 1611 -0.029 -1.000 -0.216

997 2613 1616 -0.029 -1.000 -0.216

997 2688 1691 -0.025 -1.000 -0.194

997 2889 1892 -0.004 -1.000 -0.069

997 2890 1893 -0.004 -1.000 -0.069

997 2913 1916 -0.029 -1.000 -0.216

997 2943 1946 -0.029 -1.000 -0.216

997 3384 2387 -0.025 -1.000 -0.194

997 3553 2556 -0.021 -1.000 -0.171

997 3605 2608 -0.033 -1.000 -0.239

997 3806 2809 -0.012 -1.000 -0.126

997 3859 2862 -0.025 -1.000 -0.194

997 3935 2938 -0.021 -1.000 -0.171

997 4121 3124 -0.021 -1.000 -0.171

997 4129 3132 -0.012 -1.000 -0.126

997 4137 3140 -0.021 -1.000 -0.171

997 4139 3142 -0.041 -1.000 -0.289

997 4248 3251 -0.021 -1.000 -0.171

997 4283 3286 -0.008 -1.000 -0.100

997 4400 3403 -0.008 -1.000 -0.100

1017 1040 23 0.083 1.000 1.000

1017 1067 50 0.083 1.000 1.000

1017 1091 74 0.083 1.000 1.000

1017 1099 82 0.083 1.000 1.000

1017 1121 104 0.083 1.000 1.000

1017 1122 105 0.083 1.000 1.000

1017 1132 115 0.083 1.000 1.000

1017 1158 141 -0.004 -1.000 -0.069

1017 1219 202 0.083 1.000 1.000

1017 1222 205 0.041 1.000 0.690

1017 1254 237 -0.021 -1.000 -0.171

1017 1277 260 0.083 1.000 1.000

1017 1294 277 -0.004 -1.000 -0.069

1017 1309 292 0.083 1.000 1.000

1017 1412 395 -0.017 -1.000 -0.149

1017 1486 469 0.054 1.000 0.380

1017 1490 473 0.050 1.000 0.346

1017 1499 482 0.058 1.000 0.418

1017 1510 493 0.083 1.000 1.000

1017 1602 585 0.058 1.000 0.418

1017 1623 606 -0.004 -1.000 -0.069

1017 1786 769 -0.041 -1.000 -0.289

1017 1804 787 0.058 1.000 0.418

1017 1807 790 0.054 1.000 0.380

1017 1963 946 0.054 1.000 0.380

1017 1974 957 0.058 1.000 0.418

1017 2027 1010 -0.004 -1.000 -0.069

1017 2122 1105 0.054 1.000 0.380

1017 2340 1323 -0.004 -1.000 -0.069

1017 2344 1327 -0.004 -1.000 -0.069

1017 2370 1353 -0.012 -1.000 -0.126

1017 2371 1354 -0.029 -1.000 -0.216

1017 2400 1383 -0.029 -1.000 -0.216

1017 2453 1436 -0.029 -1.000 -0.216

1017 2473 1456 -0.004 -1.000 -0.069

1017 2493 1476 -0.004 -1.000 -0.069

1017 2570 1553 -0.004 -1.000 -0.069

1017 2598 1581 -0.029 -1.000 -0.216

1017 2608 1591 -0.029 -1.000 -0.216

1017 2613 1596 -0.029 -1.000 -0.216

1017 2688 1671 -0.025 -1.000 -0.194

1017 2889 1872 -0.004 -1.000 -0.069

1017 2890 1873 -0.004 -1.000 -0.069

1017 2913 1896 -0.029 -1.000 -0.216

1017 2943 1926 -0.029 -1.000 -0.216

1017 3384 2367 -0.025 -1.000 -0.194

1017 3553 2536 -0.021 -1.000 -0.171

1017 3605 2588 0.058 1.000 0.418

1017 3806 2789 -0.012 -1.000 -0.126

1017 3859 2842 -0.025 -1.000 -0.194

1017 3935 2918 -0.021 -1.000 -0.171

1017 4121 3104 -0.021 -1.000 -0.171

1017 4129 3112 -0.012 -1.000 -0.126

1017 4137 3120 -0.021 -1.000 -0.171

1017 4139 3122 0.050 1.000 0.346

1017 4248 3231 -0.021 -1.000 -0.171

1017 4283 3266 -0.008 -1.000 -0.100

1017 4400 3383 -0.008 -1.000 -0.100

1040 1067 27 0.083 1.000 1.000

1040 1091 51 0.083 1.000 1.000

1040 1099 59 0.083 1.000 1.000

1040 1121 81 0.083 1.000 1.000

1040 1122 82 0.083 1.000 1.000

1040 1132 92 0.083 1.000 1.000

1040 1158 118 -0.004 -1.000 -0.069

1040 1219 179 0.083 1.000 1.000

1040 1222 182 0.041 1.000 0.690

1040 1254 214 -0.021 -1.000 -0.171

1040 1277 237 0.083 1.000 1.000

1040 1294 254 -0.004 -1.000 -0.069

1040 1309 269 0.083 1.000 1.000

1040 1412 372 -0.017 -1.000 -0.149

1040 1486 446 0.054 1.000 0.380

1040 1490 450 0.050 1.000 0.346

1040 1499 459 0.058 1.000 0.418

1040 1510 470 0.083 1.000 1.000

1040 1602 562 0.058 1.000 0.418

1040 1623 583 -0.004 -1.000 -0.069

1040 1786 746 -0.041 -1.000 -0.289

1040 1804 764 0.058 1.000 0.418

1040 1807 767 0.054 1.000 0.380

1040 1963 923 0.054 1.000 0.380

1040 1974 934 0.058 1.000 0.418

1040 2027 987 -0.004 -1.000 -0.069

1040 2122 1082 0.054 1.000 0.380

1040 2340 1300 -0.004 -1.000 -0.069

1040 2344 1304 -0.004 -1.000 -0.069

1040 2370 1330 -0.012 -1.000 -0.126

1040 2371 1331 -0.029 -1.000 -0.216

1040 2400 1360 -0.029 -1.000 -0.216

1040 2453 1413 -0.029 -1.000 -0.216

1040 2473 1433 -0.004 -1.000 -0.069

1040 2493 1453 -0.004 -1.000 -0.069

1040 2570 1530 -0.004 -1.000 -0.069

1040 2598 1558 -0.029 -1.000 -0.216

1040 2608 1568 -0.029 -1.000 -0.216

1040 2613 1573 -0.029 -1.000 -0.216

1040 2688 1648 -0.025 -1.000 -0.194

1040 2889 1849 -0.004 -1.000 -0.069

1040 2890 1850 -0.004 -1.000 -0.069

1040 2913 1873 -0.029 -1.000 -0.216

1040 2943 1903 -0.029 -1.000 -0.216

1040 3384 2344 -0.025 -1.000 -0.194

1040 3553 2513 -0.021 -1.000 -0.171

1040 3605 2565 0.058 1.000 0.418

1040 3806 2766 -0.012 -1.000 -0.126

1040 3859 2819 -0.025 -1.000 -0.194

1040 3935 2895 -0.021 -1.000 -0.171

1040 4121 3081 -0.021 -1.000 -0.171

1040 4129 3089 -0.012 -1.000 -0.126

1040 4137 3097 -0.021 -1.000 -0.171

1040 4139 3099 0.050 1.000 0.346

1040 4248 3208 -0.021 -1.000 -0.171

1040 4283 3243 -0.008 -1.000 -0.100

1040 4400 3360 -0.008 -1.000 -0.100

1067 1091 24 0.083 1.000 1.000

1067 1099 32 0.083 1.000 1.000

1067 1121 54 0.083 1.000 1.000

1067 1122 55 0.083 1.000 1.000

1067 1132 65 0.083 1.000 1.000

1067 1158 91 -0.004 -1.000 -0.069

1067 1219 152 0.083 1.000 1.000

1067 1222 155 0.041 1.000 0.690

1067 1254 187 -0.021 -1.000 -0.171

1067 1277 210 0.083 1.000 1.000

1067 1294 227 -0.004 -1.000 -0.069

1067 1309 242 0.083 1.000 1.000

1067 1412 345 -0.017 -1.000 -0.149

1067 1486 419 0.054 1.000 0.380

1067 1490 423 0.050 1.000 0.346

1067 1499 432 0.058 1.000 0.418

1067 1510 443 0.083 1.000 1.000

1067 1602 535 0.058 1.000 0.418

1067 1623 556 -0.004 -1.000 -0.069

1067 1786 719 -0.041 -1.000 -0.289

1067 1804 737 0.058 1.000 0.418

1067 1807 740 0.054 1.000 0.380

1067 1963 896 0.054 1.000 0.380

1067 1974 907 0.058 1.000 0.418

1067 2027 960 -0.004 -1.000 -0.069

1067 2122 1055 0.054 1.000 0.380

1067 2340 1273 -0.004 -1.000 -0.069

1067 2344 1277 -0.004 -1.000 -0.069

1067 2370 1303 -0.012 -1.000 -0.126

1067 2371 1304 -0.029 -1.000 -0.216

1067 2400 1333 -0.029 -1.000 -0.216

1067 2453 1386 -0.029 -1.000 -0.216

1067 2473 1406 -0.004 -1.000 -0.069

1067 2493 1426 -0.004 -1.000 -0.069

1067 2570 1503 -0.004 -1.000 -0.069

1067 2598 1531 -0.029 -1.000 -0.216

1067 2608 1541 -0.029 -1.000 -0.216

1067 2613 1546 -0.029 -1.000 -0.216

1067 2688 1621 -0.025 -1.000 -0.194

1067 2889 1822 -0.004 -1.000 -0.069

1067 2890 1823 -0.004 -1.000 -0.069

1067 2913 1846 -0.029 -1.000 -0.216

1067 2943 1876 -0.029 -1.000 -0.216

1067 3384 2317 -0.025 -1.000 -0.194

1067 3553 2486 -0.021 -1.000 -0.171

1067 3605 2538 0.058 1.000 0.418

1067 3806 2739 -0.012 -1.000 -0.126

1067 3859 2792 -0.025 -1.000 -0.194

1067 3935 2868 -0.021 -1.000 -0.171

1067 4121 3054 -0.021 -1.000 -0.171

1067 4129 3062 -0.012 -1.000 -0.126

1067 4137 3070 -0.021 -1.000 -0.171

1067 4139 3072 0.050 1.000 0.346

1067 4248 3181 -0.021 -1.000 -0.171

1067 4283 3216 -0.008 -1.000 -0.100

1067 4400 3333 -0.008 -1.000 -0.100

1091 1099 8 0.083 1.000 1.000

1091 1121 30 0.083 1.000 1.000

1091 1122 31 0.083 1.000 1.000

1091 1132 41 0.083 1.000 1.000

1091 1158 67 -0.004 -1.000 -0.069

1091 1219 128 0.083 1.000 1.000

1091 1222 131 0.041 1.000 0.690

1091 1254 163 -0.021 -1.000 -0.171

1091 1277 186 0.083 1.000 1.000

1091 1294 203 -0.004 -1.000 -0.069

1091 1309 218 0.083 1.000 1.000

1091 1412 321 -0.017 -1.000 -0.149

1091 1486 395 0.054 1.000 0.380

1091 1490 399 0.050 1.000 0.346

1091 1499 408 0.058 1.000 0.418

1091 1510 419 0.083 1.000 1.000

1091 1602 511 0.058 1.000 0.418

1091 1623 532 -0.004 -1.000 -0.069

1091 1786 695 -0.041 -1.000 -0.289

1091 1804 713 0.058 1.000 0.418

1091 1807 716 0.054 1.000 0.380

1091 1963 872 0.054 1.000 0.380

1091 1974 883 0.058 1.000 0.418

1091 2027 936 -0.004 -1.000 -0.069

1091 2122 1031 0.054 1.000 0.380

1091 2340 1249 -0.004 -1.000 -0.069

1091 2344 1253 -0.004 -1.000 -0.069

1091 2370 1279 -0.012 -1.000 -0.126

1091 2371 1280 -0.029 -1.000 -0.216

1091 2400 1309 -0.029 -1.000 -0.216

1091 2453 1362 -0.029 -1.000 -0.216

1091 2473 1382 -0.004 -1.000 -0.069

1091 2493 1402 -0.004 -1.000 -0.069

1091 2570 1479 -0.004 -1.000 -0.069

1091 2598 1507 -0.029 -1.000 -0.216

1091 2608 1517 -0.029 -1.000 -0.216

1091 2613 1522 -0.029 -1.000 -0.216

1091 2688 1597 -0.025 -1.000 -0.194

1091 2889 1798 -0.004 -1.000 -0.069

1091 2890 1799 -0.004 -1.000 -0.069

1091 2913 1822 -0.029 -1.000 -0.216

1091 2943 1852 -0.029 -1.000 -0.216

1091 3384 2293 -0.025 -1.000 -0.194

1091 3553 2462 -0.021 -1.000 -0.171

1091 3605 2514 0.058 1.000 0.418

1091 3806 2715 -0.012 -1.000 -0.126

1091 3859 2768 -0.025 -1.000 -0.194

1091 3935 2844 -0.021 -1.000 -0.171

1091 4121 3030 -0.021 -1.000 -0.171

1091 4129 3038 -0.012 -1.000 -0.126

1091 4137 3046 -0.021 -1.000 -0.171

1091 4139 3048 0.050 1.000 0.346

1091 4248 3157 -0.021 -1.000 -0.171

1091 4283 3192 -0.008 -1.000 -0.100

1091 4400 3309 -0.008 -1.000 -0.100

1099 1121 22 0.083 1.000 1.000

1099 1122 23 0.083 1.000 1.000

1099 1132 33 0.083 1.000 1.000

1099 1158 59 -0.004 -1.000 -0.069

1099 1219 120 0.083 1.000 1.000

1099 1222 123 0.041 1.000 0.690

1099 1254 155 -0.021 -1.000 -0.171

1099 1277 178 0.083 1.000 1.000

1099 1294 195 -0.004 -1.000 -0.069

1099 1309 210 0.083 1.000 1.000

1099 1412 313 -0.017 -1.000 -0.149

1099 1486 387 0.054 1.000 0.380

1099 1490 391 0.050 1.000 0.346

1099 1499 400 0.058 1.000 0.418

1099 1510 411 0.083 1.000 1.000

1099 1602 503 0.058 1.000 0.418

1099 1623 524 -0.004 -1.000 -0.069

1099 1786 687 -0.041 -1.000 -0.289

1099 1804 705 0.058 1.000 0.418

1099 1807 708 0.054 1.000 0.380

1099 1963 864 0.054 1.000 0.380

1099 1974 875 0.058 1.000 0.418

1099 2027 928 -0.004 -1.000 -0.069

1099 2122 1023 0.054 1.000 0.380

1099 2340 1241 -0.004 -1.000 -0.069

1099 2344 1245 -0.004 -1.000 -0.069

1099 2370 1271 -0.012 -1.000 -0.126

1099 2371 1272 -0.029 -1.000 -0.216

1099 2400 1301 -0.029 -1.000 -0.216

1099 2453 1354 -0.029 -1.000 -0.216

1099 2473 1374 -0.004 -1.000 -0.069

1099 2493 1394 -0.004 -1.000 -0.069

1099 2570 1471 -0.004 -1.000 -0.069

1099 2598 1499 -0.029 -1.000 -0.216

1099 2608 1509 -0.029 -1.000 -0.216

1099 2613 1514 -0.029 -1.000 -0.216

1099 2688 1589 -0.025 -1.000 -0.194

1099 2889 1790 -0.004 -1.000 -0.069

1099 2890 1791 -0.004 -1.000 -0.069

1099 2913 1814 -0.029 -1.000 -0.216

1099 2943 1844 -0.029 -1.000 -0.216

1099 3384 2285 -0.025 -1.000 -0.194

1099 3553 2454 -0.021 -1.000 -0.171

1099 3605 2506 0.058 1.000 0.418

1099 3806 2707 -0.012 -1.000 -0.126

1099 3859 2760 -0.025 -1.000 -0.194

1099 3935 2836 -0.021 -1.000 -0.171

1099 4121 3022 -0.021 -1.000 -0.171

1099 4129 3030 -0.012 -1.000 -0.126

1099 4137 3038 -0.021 -1.000 -0.171

1099 4139 3040 0.050 1.000 0.346

1099 4248 3149 -0.021 -1.000 -0.171

1099 4283 3184 -0.008 -1.000 -0.100

1099 4400 3301 -0.008 -1.000 -0.100

1121 1122 1 0.083 1.000 1.000

1121 1132 11 0.083 1.000 1.000

1121 1158 37 -0.004 -1.000 -0.069

1121 1219 98 0.083 1.000 1.000

1121 1222 101 0.041 1.000 0.690

1121 1254 133 -0.021 -1.000 -0.171

1121 1277 156 0.083 1.000 1.000

1121 1294 173 -0.004 -1.000 -0.069

1121 1309 188 0.083 1.000 1.000

1121 1412 291 -0.017 -1.000 -0.149

1121 1486 365 0.054 1.000 0.380

1121 1490 369 0.050 1.000 0.346

1121 1499 378 0.058 1.000 0.418

1121 1510 389 0.083 1.000 1.000

1121 1602 481 0.058 1.000 0.418

1121 1623 502 -0.004 -1.000 -0.069

1121 1786 665 -0.041 -1.000 -0.289

1121 1804 683 0.058 1.000 0.418

1121 1807 686 0.054 1.000 0.380

1121 1963 842 0.054 1.000 0.380

1121 1974 853 0.058 1.000 0.418

1121 2027 906 -0.004 -1.000 -0.069

1121 2122 1001 0.054 1.000 0.380

1121 2340 1219 -0.004 -1.000 -0.069

1121 2344 1223 -0.004 -1.000 -0.069

1121 2370 1249 -0.012 -1.000 -0.126

1121 2371 1250 -0.029 -1.000 -0.216

1121 2400 1279 -0.029 -1.000 -0.216

1121 2453 1332 -0.029 -1.000 -0.216

1121 2473 1352 -0.004 -1.000 -0.069

1121 2493 1372 -0.004 -1.000 -0.069

1121 2570 1449 -0.004 -1.000 -0.069

1121 2598 1477 -0.029 -1.000 -0.216

1121 2608 1487 -0.029 -1.000 -0.216

1121 2613 1492 -0.029 -1.000 -0.216

1121 2688 1567 -0.025 -1.000 -0.194

1121 2889 1768 -0.004 -1.000 -0.069

1121 2890 1769 -0.004 -1.000 -0.069

1121 2913 1792 -0.029 -1.000 -0.216

1121 2943 1822 -0.029 -1.000 -0.216

1121 3384 2263 -0.025 -1.000 -0.194

1121 3553 2432 -0.021 -1.000 -0.171

1121 3605 2484 0.058 1.000 0.418

1121 3806 2685 -0.012 -1.000 -0.126

1121 3859 2738 -0.025 -1.000 -0.194

1121 3935 2814 -0.021 -1.000 -0.171

1121 4121 3000 -0.021 -1.000 -0.171

1121 4129 3008 -0.012 -1.000 -0.126

1121 4137 3016 -0.021 -1.000 -0.171

1121 4139 3018 0.050 1.000 0.346

1121 4248 3127 -0.021 -1.000 -0.171

1121 4283 3162 -0.008 -1.000 -0.100

1121 4400 3279 -0.008 -1.000 -0.100

1122 1132 10 0.083 1.000 1.000

1122 1158 36 -0.004 -1.000 -0.069

1122 1219 97 0.083 1.000 1.000

1122 1222 100 0.041 1.000 0.690

1122 1254 132 -0.021 -1.000 -0.171

1122 1277 155 0.083 1.000 1.000

1122 1294 172 -0.004 -1.000 -0.069

1122 1309 187 0.083 1.000 1.000

1122 1412 290 -0.017 -1.000 -0.149

1122 1486 364 0.054 1.000 0.380

1122 1490 368 0.050 1.000 0.346

1122 1499 377 0.058 1.000 0.418

1122 1510 388 0.083 1.000 1.000

1122 1602 480 0.058 1.000 0.418

1122 1623 501 -0.004 -1.000 -0.069

1122 1786 664 -0.041 -1.000 -0.289

1122 1804 682 0.058 1.000 0.418

1122 1807 685 0.054 1.000 0.380

1122 1963 841 0.054 1.000 0.380

1122 1974 852 0.058 1.000 0.418

1122 2027 905 -0.004 -1.000 -0.069

1122 2122 1000 0.054 1.000 0.380

1122 2340 1218 -0.004 -1.000 -0.069

1122 2344 1222 -0.004 -1.000 -0.069

1122 2370 1248 -0.012 -1.000 -0.126

1122 2371 1249 -0.029 -1.000 -0.216

1122 2400 1278 -0.029 -1.000 -0.216

1122 2453 1331 -0.029 -1.000 -0.216

1122 2473 1351 -0.004 -1.000 -0.069

1122 2493 1371 -0.004 -1.000 -0.069

1122 2570 1448 -0.004 -1.000 -0.069

1122 2598 1476 -0.029 -1.000 -0.216

1122 2608 1486 -0.029 -1.000 -0.216

1122 2613 1491 -0.029 -1.000 -0.216

1122 2688 1566 -0.025 -1.000 -0.194

1122 2889 1767 -0.004 -1.000 -0.069

1122 2890 1768 -0.004 -1.000 -0.069

1122 2913 1791 -0.029 -1.000 -0.216

1122 2943 1821 -0.029 -1.000 -0.216

1122 3384 2262 -0.025 -1.000 -0.194

1122 3553 2431 -0.021 -1.000 -0.171

1122 3605 2483 0.058 1.000 0.418

1122 3806 2684 -0.012 -1.000 -0.126

1122 3859 2737 -0.025 -1.000 -0.194

1122 3935 2813 -0.021 -1.000 -0.171

1122 4121 2999 -0.021 -1.000 -0.171

1122 4129 3007 -0.012 -1.000 -0.126

1122 4137 3015 -0.021 -1.000 -0.171

1122 4139 3017 0.050 1.000 0.346

1122 4248 3126 -0.021 -1.000 -0.171

1122 4283 3161 -0.008 -1.000 -0.100

1122 4400 3278 -0.008 -1.000 -0.100

1132 1158 26 -0.004 -1.000 -0.069

1132 1219 87 0.083 1.000 1.000

1132 1222 90 0.041 1.000 0.690

1132 1254 122 -0.021 -1.000 -0.171

1132 1277 145 0.083 1.000 1.000

1132 1294 162 -0.004 -1.000 -0.069

1132 1309 177 0.083 1.000 1.000

1132 1412 280 -0.017 -1.000 -0.149

1132 1486 354 0.054 1.000 0.380

1132 1490 358 0.050 1.000 0.346

1132 1499 367 0.058 1.000 0.418

1132 1510 378 0.083 1.000 1.000

1132 1602 470 0.058 1.000 0.418

1132 1623 491 -0.004 -1.000 -0.069

1132 1786 654 -0.041 -1.000 -0.289

1132 1804 672 0.058 1.000 0.418

1132 1807 675 0.054 1.000 0.380

1132 1963 831 0.054 1.000 0.380

1132 1974 842 0.058 1.000 0.418

1132 2027 895 -0.004 -1.000 -0.069

1132 2122 990 0.054 1.000 0.380

1132 2340 1208 -0.004 -1.000 -0.069

1132 2344 1212 -0.004 -1.000 -0.069

1132 2370 1238 -0.012 -1.000 -0.126

1132 2371 1239 -0.029 -1.000 -0.216

1132 2400 1268 -0.029 -1.000 -0.216

1132 2453 1321 -0.029 -1.000 -0.216

1132 2473 1341 -0.004 -1.000 -0.069

1132 2493 1361 -0.004 -1.000 -0.069

1132 2570 1438 -0.004 -1.000 -0.069

1132 2598 1466 -0.029 -1.000 -0.216

1132 2608 1476 -0.029 -1.000 -0.216

1132 2613 1481 -0.029 -1.000 -0.216

1132 2688 1556 -0.025 -1.000 -0.194

1132 2889 1757 -0.004 -1.000 -0.069

1132 2890 1758 -0.004 -1.000 -0.069

1132 2913 1781 -0.029 -1.000 -0.216

1132 2943 1811 -0.029 -1.000 -0.216

1132 3384 2252 -0.025 -1.000 -0.194

1132 3553 2421 -0.021 -1.000 -0.171

1132 3605 2473 0.058 1.000 0.418

1132 3806 2674 -0.012 -1.000 -0.126

1132 3859 2727 -0.025 -1.000 -0.194

1132 3935 2803 -0.021 -1.000 -0.171

1132 4121 2989 -0.021 -1.000 -0.171

1132 4129 2997 -0.012 -1.000 -0.126

1132 4137 3005 -0.021 -1.000 -0.171

1132 4139 3007 0.050 1.000 0.346

1132 4248 3116 -0.021 -1.000 -0.171

1132 4283 3151 -0.008 -1.000 -0.100

1132 4400 3268 -0.008 -1.000 -0.100

1158 1219 61 -0.004 -1.000 -0.069

1158 1222 64 -0.002 -1.000 -0.048

1158 1254 96 -0.010 -1.000 -0.118

1158 1277 119 -0.004 -1.000 -0.069

1158 1294 136 -0.002 -1.000 -0.048

1158 1309 151 -0.004 -1.000 -0.069

1158 1412 254 -0.008 -1.000 -0.103

1158 1486 328 -0.019 -1.000 -0.182

1158 1490 332 0.025 1.000 0.239

1158 1499 341 -0.017 -1.000 -0.165

1158 1510 352 -0.004 -1.000 -0.069

1158 1602 444 -0.017 -1.000 -0.165

1158 1623 465 -0.002 -1.000 -0.048

1158 1786 628 0.025 1.000 0.239

1158 1804 646 -0.017 -1.000 -0.165

1158 1807 649 -0.019 -1.000 -0.182

1158 1963 805 -0.019 -1.000 -0.182

1158 1974 816 -0.017 -1.000 -0.165

1158 2027 869 0.043 1.000 1.000

1158 2122 964 -0.019 -1.000 -0.182

1158 2340 1182 -0.002 -1.000 -0.048

1158 2344 1186 -0.002 -1.000 -0.048

1158 2370 1212 -0.006 -1.000 -0.087

1158 2371 1213 0.031 1.000 0.319

1158 2400 1242 0.031 1.000 0.319

1158 2453 1295 0.031 1.000 0.319

1158 2473 1315 -0.002 -1.000 -0.048

1158 2493 1335 -0.002 -1.000 -0.048

1158 2570 1412 -0.002 -1.000 -0.048

1158 2598 1440 0.031 1.000 0.319

1158 2608 1450 0.031 1.000 0.319

1158 2613 1455 0.031 1.000 0.319

1158 2688 1530 -0.012 -1.000 -0.134

1158 2889 1731 0.043 1.000 1.000

1158 2890 1732 0.043 1.000 1.000

1158 2913 1755 0.031 1.000 0.319

1158 2943 1785 0.031 1.000 0.319

1158 3384 2226 0.033 1.000 0.356

1158 3553 2395 0.035 1.000 0.402

1158 3605 2447 -0.017 -1.000 -0.165

1158 3806 2648 0.039 1.000 0.549

1158 3859 2701 0.033 1.000 0.356

1158 3935 2777 0.035 1.000 0.402

1158 4121 2963 0.035 1.000 0.402

1158 4129 2971 0.039 1.000 0.549

1158 4137 2979 0.035 1.000 0.402

1158 4139 2981 -0.021 -1.000 -0.199

1158 4248 3090 0.035 1.000 0.402

1158 4283 3125 -0.004 -1.000 -0.069

1158 4400 3242 -0.004 -1.000 -0.069

1219 1222 3 0.041 1.000 0.690

1219 1254 35 -0.021 -1.000 -0.171

1219 1277 58 0.083 1.000 1.000

1219 1294 75 -0.004 -1.000 -0.069

1219 1309 90 0.083 1.000 1.000

1219 1412 193 -0.017 -1.000 -0.149

1219 1486 267 0.054 1.000 0.380

1219 1490 271 0.050 1.000 0.346

1219 1499 280 0.058 1.000 0.418

1219 1510 291 0.083 1.000 1.000

1219 1602 383 0.058 1.000 0.418

1219 1623 404 -0.004 -1.000 -0.069

1219 1786 567 -0.041 -1.000 -0.289

1219 1804 585 0.058 1.000 0.418

1219 1807 588 0.054 1.000 0.380

1219 1963 744 0.054 1.000 0.380

1219 1974 755 0.058 1.000 0.418

1219 2027 808 -0.004 -1.000 -0.069

1219 2122 903 0.054 1.000 0.380

1219 2340 1121 -0.004 -1.000 -0.069

1219 2344 1125 -0.004 -1.000 -0.069

1219 2370 1151 -0.012 -1.000 -0.126

1219 2371 1152 -0.029 -1.000 -0.216

1219 2400 1181 -0.029 -1.000 -0.216

1219 2453 1234 -0.029 -1.000 -0.216

1219 2473 1254 -0.004 -1.000 -0.069

1219 2493 1274 -0.004 -1.000 -0.069

1219 2570 1351 -0.004 -1.000 -0.069

1219 2598 1379 -0.029 -1.000 -0.216

1219 2608 1389 -0.029 -1.000 -0.216

1219 2613 1394 -0.029 -1.000 -0.216

1219 2688 1469 -0.025 -1.000 -0.194

1219 2889 1670 -0.004 -1.000 -0.069

1219 2890 1671 -0.004 -1.000 -0.069

1219 2913 1694 -0.029 -1.000 -0.216

1219 2943 1724 -0.029 -1.000 -0.216

1219 3384 2165 -0.025 -1.000 -0.194

1219 3553 2334 -0.021 -1.000 -0.171

1219 3605 2386 0.058 1.000 0.418

1219 3806 2587 -0.012 -1.000 -0.126

1219 3859 2640 -0.025 -1.000 -0.194

1219 3935 2716 -0.021 -1.000 -0.171

1219 4121 2902 -0.021 -1.000 -0.171

1219 4129 2910 -0.012 -1.000 -0.126

1219 4137 2918 -0.021 -1.000 -0.171

1219 4139 2920 0.050 1.000 0.346

1219 4248 3029 -0.021 -1.000 -0.171

1219 4283 3064 -0.008 -1.000 -0.100

1219 4400 3181 -0.008 -1.000 -0.100

1222 1254 32 -0.010 -1.000 -0.118

1222 1277 55 0.041 1.000 0.690

1222 1294 72 -0.002 -1.000 -0.048

1222 1309 87 0.041 1.000 0.690

1222 1412 190 -0.008 -1.000 -0.103

1222 1486 264 0.027 1.000 0.262

1222 1490 268 0.025 1.000 0.239

1222 1499 277 0.029 1.000 0.289

1222 1510 288 0.041 1.000 0.690

1222 1602 380 0.029 1.000 0.289

1222 1623 401 -0.002 -1.000 -0.048

1222 1786 564 -0.021 -1.000 -0.199

1222 1804 582 0.029 1.000 0.289

1222 1807 585 0.027 1.000 0.262

1222 1963 741 0.027 1.000 0.262

1222 1974 752 0.029 1.000 0.289

1222 2027 805 -0.002 -1.000 -0.048

1222 2122 900 0.027 1.000 0.262

1222 2340 1118 -0.002 -1.000 -0.048

1222 2344 1122 -0.002 -1.000 -0.048

1222 2370 1148 -0.006 -1.000 -0.087

1222 2371 1149 -0.014 -1.000 -0.149

1222 2400 1178 -0.014 -1.000 -0.149

1222 2453 1231 -0.014 -1.000 -0.149

1222 2473 1251 -0.002 -1.000 -0.048

1222 2493 1271 -0.002 -1.000 -0.048

1222 2570 1348 -0.002 -1.000 -0.048

1222 2598 1376 -0.014 -1.000 -0.149

1222 2608 1386 -0.014 -1.000 -0.149

1222 2613 1391 -0.014 -1.000 -0.149

1222 2688 1466 -0.012 -1.000 -0.134

1222 2889 1667 -0.002 -1.000 -0.048

1222 2890 1668 -0.002 -1.000 -0.048

1222 2913 1691 -0.014 -1.000 -0.149

1222 2943 1721 -0.014 -1.000 -0.149

1222 3384 2162 -0.012 -1.000 -0.134

1222 3553 2331 -0.010 -1.000 -0.118

1222 3605 2383 0.029 1.000 0.289

1222 3806 2584 -0.006 -1.000 -0.087

1222 3859 2637 -0.012 -1.000 -0.134

1222 3935 2713 -0.010 -1.000 -0.118

1222 4121 2899 -0.010 -1.000 -0.118

1222 4129 2907 -0.006 -1.000 -0.087

1222 4137 2915 -0.010 -1.000 -0.118

1222 4139 2917 0.025 1.000 0.239

1222 4248 3026 -0.010 -1.000 -0.118

1222 4283 3061 -0.004 -1.000 -0.069

1222 4400 3178 -0.004 -1.000 -0.069

1254 1277 23 -0.021 -1.000 -0.171

1254 1294 40 -0.010 -1.000 -0.118

1254 1309 55 -0.021 -1.000 -0.171

1254 1412 158 -0.041 -1.000 -0.256

1254 1486 232 0.089 0.662 0.431

1254 1490 236 -0.058 -0.560 -0.277

1254 1499 245 0.099 0.686 0.492

1254 1510 256 -0.021 -1.000 -0.171

1254 1602 348 0.054 0.371 0.266

1254 1623 369 -0.010 -1.000 -0.118

1254 1786 532 -0.012 -0.120 -0.059

1254 1804 550 0.054 0.371 0.266

1254 1807 553 0.043 0.323 0.211

1254 1963 709 0.043 0.323 0.211

1254 1974 720 0.054 0.371 0.266

1254 2027 773 -0.010 -1.000 -0.118

1254 2122 868 0.043 0.323 0.211

1254 2340 1086 -0.010 -1.000 -0.118

1254 2344 1090 -0.010 -1.000 -0.118

1254 2370 1116 0.014 0.137 0.101

1254 2371 1117 -0.027 -0.371 -0.138

1254 2400 1146 -0.027 -0.371 -0.138

1254 2453 1199 -0.027 -0.371 -0.138

1254 2473 1219 -0.010 -1.000 -0.118

1254 2493 1239 -0.010 -1.000 -0.118

1254 2570 1316 -0.010 -1.000 -0.118

1254 2598 1344 -0.027 -0.371 -0.138

1254 2608 1354 -0.027 -0.371 -0.138

1254 2613 1359 -0.027 -0.371 -0.138

1254 2688 1434 -0.017 -0.267 -0.089

1254 2889 1635 -0.010 -1.000 -0.118

1254 2890 1636 -0.010 -1.000 -0.118

1254 2913 1659 -0.027 -0.371 -0.138

1254 2943 1689 -0.027 -0.371 -0.138

1254 3384 2130 -0.017 -0.267 -0.089

1254 3553 2299 -0.006 -0.120 -0.035

1254 3605 2351 0.054 0.371 0.266

1254 3806 2552 -0.031 -1.000 -0.215

1254 3859 2605 -0.017 -0.267 -0.089

1254 3935 2681 -0.006 -0.120 -0.035

1254 4121 2867 -0.006 -0.120 -0.035

1254 4129 2875 0.014 0.137 0.101

1254 4137 2883 -0.006 -0.120 -0.035

1254 4139 2885 0.033 0.267 0.158

1254 4248 2994 -0.006 -0.120 -0.035

1254 4283 3029 0.025 0.353 0.206

1254 4400 3146 -0.021 -1.000 -0.171

1277 1294 17 -0.004 -1.000 -0.069

1277 1309 32 0.083 1.000 1.000

1277 1412 135 -0.017 -1.000 -0.149

1277 1486 209 0.054 1.000 0.380

1277 1490 213 0.050 1.000 0.346

1277 1499 222 0.058 1.000 0.418

1277 1510 233 0.083 1.000 1.000

1277 1602 325 0.058 1.000 0.418

1277 1623 346 -0.004 -1.000 -0.069

1277 1786 509 -0.041 -1.000 -0.289

1277 1804 527 0.058 1.000 0.418

1277 1807 530 0.054 1.000 0.380

1277 1963 686 0.054 1.000 0.380

1277 1974 697 0.058 1.000 0.418

1277 2027 750 -0.004 -1.000 -0.069

1277 2122 845 0.054 1.000 0.380

1277 2340 1063 -0.004 -1.000 -0.069

1277 2344 1067 -0.004 -1.000 -0.069

1277 2370 1093 -0.012 -1.000 -0.126

1277 2371 1094 -0.029 -1.000 -0.216

1277 2400 1123 -0.029 -1.000 -0.216

1277 2453 1176 -0.029 -1.000 -0.216

1277 2473 1196 -0.004 -1.000 -0.069

1277 2493 1216 -0.004 -1.000 -0.069

1277 2570 1293 -0.004 -1.000 -0.069

1277 2598 1321 -0.029 -1.000 -0.216

1277 2608 1331 -0.029 -1.000 -0.216

1277 2613 1336 -0.029 -1.000 -0.216

1277 2688 1411 -0.025 -1.000 -0.194

1277 2889 1612 -0.004 -1.000 -0.069

1277 2890 1613 -0.004 -1.000 -0.069

1277 2913 1636 -0.029 -1.000 -0.216

1277 2943 1666 -0.029 -1.000 -0.216

1277 3384 2107 -0.025 -1.000 -0.194

1277 3553 2276 -0.021 -1.000 -0.171

1277 3605 2328 0.058 1.000 0.418

1277 3806 2529 -0.012 -1.000 -0.126

1277 3859 2582 -0.025 -1.000 -0.194

1277 3935 2658 -0.021 -1.000 -0.171

1277 4121 2844 -0.021 -1.000 -0.171

1277 4129 2852 -0.012 -1.000 -0.126

1277 4137 2860 -0.021 -1.000 -0.171

1277 4139 2862 0.050 1.000 0.346

1277 4248 2971 -0.021 -1.000 -0.171

1277 4283 3006 -0.008 -1.000 -0.100

1277 4400 3123 -0.008 -1.000 -0.100

1294 1309 15 -0.004 -1.000 -0.069

1294 1412 118 -0.008 -1.000 -0.103

1294 1486 192 -0.019 -1.000 -0.182

1294 1490 196 -0.021 -1.000 -0.199

1294 1499 205 -0.017 -1.000 -0.165

1294 1510 216 -0.004 -1.000 -0.069

1294 1602 308 -0.017 -1.000 -0.165

1294 1623 329 -0.002 -1.000 -0.048

1294 1786 492 0.025 1.000 0.239

1294 1804 510 -0.017 -1.000 -0.165

1294 1807 513 -0.019 -1.000 -0.182

1294 1963 669 -0.019 -1.000 -0.182

1294 1974 680 -0.017 -1.000 -0.165

1294 2027 733 -0.002 -1.000 -0.048

1294 2122 828 -0.019 -1.000 -0.182

1294 2340 1046 -0.002 -1.000 -0.048

1294 2344 1050 -0.002 -1.000 -0.048

1294 2370 1076 -0.006 -1.000 -0.087

1294 2371 1077 0.031 1.000 0.319

1294 2400 1106 0.031 1.000 0.319

1294 2453 1159 0.031 1.000 0.319

1294 2473 1179 -0.002 -1.000 -0.048

1294 2493 1199 -0.002 -1.000 -0.048

1294 2570 1276 -0.002 -1.000 -0.048

1294 2598 1304 0.031 1.000 0.319

1294 2608 1314 0.031 1.000 0.319

1294 2613 1319 0.031 1.000 0.319

1294 2688 1394 0.033 1.000 0.356

1294 2889 1595 -0.002 -1.000 -0.048

1294 2890 1596 -0.002 -1.000 -0.048

1294 2913 1619 0.031 1.000 0.319

1294 2943 1649 0.031 1.000 0.319

1294 3384 2090 0.033 1.000 0.356

1294 3553 2259 0.035 1.000 0.402

1294 3605 2311 -0.017 -1.000 -0.165

1294 3806 2512 -0.006 -1.000 -0.087

1294 3859 2565 0.033 1.000 0.356

1294 3935 2641 0.035 1.000 0.402

1294 4121 2827 0.035 1.000 0.402

1294 4129 2835 -0.006 -1.000 -0.087

1294 4137 2843 0.035 1.000 0.402

1294 4139 2845 -0.021 -1.000 -0.199

1294 4248 2954 0.035 1.000 0.402

1294 4283 2989 -0.004 -1.000 -0.069

1294 4400 3106 0.041 1.000 0.690

1309 1412 103 -0.017 -1.000 -0.149

1309 1486 177 0.054 1.000 0.380

1309 1490 181 0.050 1.000 0.346

1309 1499 190 0.058 1.000 0.418

1309 1510 201 0.083 1.000 1.000

1309 1602 293 0.058 1.000 0.418

1309 1623 314 -0.004 -1.000 -0.069

1309 1786 477 -0.041 -1.000 -0.289

1309 1804 495 0.058 1.000 0.418

1309 1807 498 0.054 1.000 0.380

1309 1963 654 0.054 1.000 0.380

1309 1974 665 0.058 1.000 0.418

1309 2027 718 -0.004 -1.000 -0.069

1309 2122 813 0.054 1.000 0.380

1309 2340 1031 -0.004 -1.000 -0.069

1309 2344 1035 -0.004 -1.000 -0.069

1309 2370 1061 -0.012 -1.000 -0.126

1309 2371 1062 -0.029 -1.000 -0.216

1309 2400 1091 -0.029 -1.000 -0.216

1309 2453 1144 -0.029 -1.000 -0.216

1309 2473 1164 -0.004 -1.000 -0.069

1309 2493 1184 -0.004 -1.000 -0.069

1309 2570 1261 -0.004 -1.000 -0.069

1309 2598 1289 -0.029 -1.000 -0.216

1309 2608 1299 -0.029 -1.000 -0.216

1309 2613 1304 -0.029 -1.000 -0.216

1309 2688 1379 -0.025 -1.000 -0.194

1309 2889 1580 -0.004 -1.000 -0.069

1309 2890 1581 -0.004 -1.000 -0.069

1309 2913 1604 -0.029 -1.000 -0.216

1309 2943 1634 -0.029 -1.000 -0.216

1309 3384 2075 -0.025 -1.000 -0.194

1309 3553 2244 -0.021 -1.000 -0.171

1309 3605 2296 0.058 1.000 0.418

1309 3806 2497 -0.012 -1.000 -0.126

1309 3859 2550 -0.025 -1.000 -0.194

1309 3935 2626 -0.021 -1.000 -0.171

1309 4121 2812 -0.021 -1.000 -0.171

1309 4129 2820 -0.012 -1.000 -0.126

1309 4137 2828 -0.021 -1.000 -0.171

1309 4139 2830 0.050 1.000 0.346

1309 4248 2939 -0.021 -1.000 -0.171

1309 4283 2974 -0.008 -1.000 -0.100

1309 4400 3091 -0.008 -1.000 -0.100

1412 1486 74 0.017 0.154 0.087

1412 1490 78 0.054 0.542 0.280

1412 1499 87 -0.021 -0.313 -0.111

1412 1510 98 -0.017 -1.000 -0.149

1412 1602 190 0.025 0.214 0.134

1412 1623 211 -0.008 -1.000 -0.103

1412 1786 374 0.008 0.083 0.043

1412 1804 392 -0.066 -1.000 -0.356

1412 1807 395 -0.074 -1.000 -0.392

1412 1963 551 -0.074 -1.000 -0.392

1412 1974 562 -0.066 -1.000 -0.356

1412 2027 615 -0.008 -1.000 -0.103

1412 2122 710 -0.074 -1.000 -0.392

1412 2340 928 -0.008 -1.000 -0.103

1412 2344 932 -0.008 -1.000 -0.103

1412 2370 958 -0.025 -1.000 -0.187

1412 2371 959 -0.058 -1.000 -0.322

1412 2400 988 -0.058 -1.000 -0.322

1412 2453 1041 -0.058 -1.000 -0.322

1412 2473 1061 -0.008 -1.000 -0.103

1412 2493 1081 -0.008 -1.000 -0.103

1412 2570 1158 -0.008 -1.000 -0.103

1412 2598 1186 -0.058 -1.000 -0.322

1412 2608 1196 -0.058 -1.000 -0.322

1412 2613 1201 -0.058 -1.000 -0.322

1412 2688 1276 -0.050 -1.000 -0.289

1412 2889 1477 -0.008 -1.000 -0.103

1412 2890 1478 -0.008 -1.000 -0.103

1412 2913 1501 -0.058 -1.000 -0.322

1412 2943 1531 -0.058 -1.000 -0.322

1412 3384 1972 -0.050 -1.000 -0.289

1412 3553 2141 -0.041 -1.000 -0.256

1412 3605 2193 -0.066 -1.000 -0.356

1412 3806 2394 -0.025 -1.000 -0.187

1412 3859 2447 -0.050 -1.000 -0.289

1412 3935 2523 -0.041 -1.000 -0.256

1412 4121 2709 -0.041 -1.000 -0.256

1412 4129 2717 -0.025 -1.000 -0.187

1412 4137 2725 -0.041 -1.000 -0.256

1412 4139 2727 -0.083 -1.000 -0.430

1412 4248 2836 -0.041 -1.000 -0.256

1412 4283 2871 -0.017 -1.000 -0.149

1412 4400 2988 -0.017 -1.000 -0.149

1486 1490 4 0.041 0.185 0.169

1486 1499 13 0.215 1.000 0.909

1486 1510 24 0.054 1.000 0.380

1486 1602 116 0.215 1.000 0.909

1486 1623 137 -0.019 -1.000 -0.182

1486 1786 300 -0.140 -0.756 -0.574

1486 1804 318 0.079 0.365 0.332

1486 1807 321 0.105 0.436 0.436

1486 1963 477 0.105 0.436 0.436

1486 1974 488 0.079 0.365 0.332

1486 2027 541 -0.019 -1.000 -0.182

1486 2122 636 0.060 0.248 0.248

1486 2340 854 -0.019 -1.000 -0.182

1486 2344 858 -0.019 -1.000 -0.182

1486 2370 884 -0.010 -0.185 -0.061

1486 2371 885 -0.085 -0.651 -0.370

1486 2400 914 -0.085 -0.651 -0.370

1486 2453 967 -0.085 -0.651 -0.370

1486 2473 987 -0.019 -1.000 -0.182

1486 2493 1007 -0.019 -1.000 -0.182

1486 2570 1084 -0.019 -1.000 -0.182

1486 2598 1112 -0.085 -0.651 -0.370

1486 2608 1122 -0.085 -0.651 -0.370

1486 2613 1127 -0.085 -0.651 -0.370

1486 2688 1202 -0.066 -0.593 -0.302

1486 2889 1403 -0.019 -1.000 -0.182

1486 2890 1404 -0.019 -1.000 -0.182

1486 2913 1427 -0.085 -0.651 -0.370

1486 2943 1457 -0.085 -0.651 -0.370

1486 3384 1898 -0.066 -0.593 -0.302

1486 3553 2067 -0.048 -0.511 -0.231

1486 3605 2119 0.079 0.365 0.332

1486 3806 2320 -0.056 -1.000 -0.331

1486 3859 2373 -0.066 -0.593 -0.302

1486 3935 2449 -0.048 -0.511 -0.231

1486 4121 2635 -0.048 -0.511 -0.231

1486 4129 2643 -0.010 -0.185 -0.061

1486 4137 2651 -0.048 -0.511 -0.231

1486 4139 2653 0.087 0.389 0.354

1486 4248 2762 -0.048 -0.511 -0.231

1486 4283 2797 -0.037 -1.000 -0.263

1486 4400 2914 -0.037 -1.000 -0.263

1490 1499 9 0.017 0.083 0.069

1490 1510 20 0.050 1.000 0.346

1490 1602 112 0.062 0.313 0.259

1490 1623 133 -0.021 -1.000 -0.199

1490 1786 296 0.021 0.083 0.083

1490 1804 314 -0.029 -0.175 -0.121

1490 1807 317 -0.050 -0.267 -0.203

1490 1963 473 -0.050 -0.267 -0.203

1490 1974 484 -0.029 -0.175 -0.121

1490 2027 537 0.025 1.000 0.239

1490 2122 632 -0.050 -0.267 -0.203

1490 2340 850 -0.021 -1.000 -0.199

1490 2344 854 -0.021 -1.000 -0.199

1490 2370 880 -0.017 -0.267 -0.097

1490 2371 881 -0.008 -0.057 -0.036

1490 2400 910 -0.008 -0.057 -0.036

1490 2453 963 -0.008 -0.057 -0.036

1490 2473 983 -0.021 -1.000 -0.199

1490 2493 1003 -0.021 -1.000 -0.199

1490 2570 1080 -0.021 -1.000 -0.199

1490 2598 1108 -0.008 -0.057 -0.036

1490 2608 1118 -0.008 -0.057 -0.036

1490 2613 1123 -0.008 -0.057 -0.036

1490 2688 1198 -0.033 -0.267 -0.149

1490 2889 1399 0.025 1.000 0.239

1490 2890 1400 0.025 1.000 0.239

1490 2913 1423 -0.008 -0.057 -0.036

1490 2943 1453 -0.008 -0.057 -0.036

1490 3384 1894 -0.033 -0.267 -0.149

1490 3553 2063 -0.058 -0.560 -0.277

1490 3605 2115 -0.029 -0.175 -0.121

1490 3806 2316 0.029 0.389 0.169

1490 3859 2369 -0.033 -0.267 -0.149

1490 3935 2445 -0.012 -0.120 -0.059

1490 4121 2631 -0.012 -0.120 -0.059

1490 4129 2639 0.029 0.389 0.169

1490 4137 2647 -0.012 -0.120 -0.059

1490 4139 2649 -0.070 -0.340 -0.283

1490 4248 2758 -0.012 -0.120 -0.059

1490 4283 2793 0.004 0.083 0.029

1490 4400 2910 -0.041 -1.000 -0.289

1499 1510 11 0.058 1.000 0.418

1499 1602 103 0.186 0.804 0.804

1499 1623 124 -0.017 -1.000 -0.165

1499 1786 287 -0.120 -0.725 -0.500

1499 1804 305 0.095 0.411 0.411

1499 1807 308 0.124 0.577 0.524

1499 1963 464 0.124 0.577 0.524

1499 1974 475 0.095 0.411 0.411

1499 2027 528 -0.017 -1.000 -0.165

1499 2122 623 0.079 0.365 0.332

1499 2340 841 -0.017 -1.000 -0.165

1499 2344 845 -0.017 -1.000 -0.165

1499 2370 871 -0.004 -0.083 -0.025

1499 2371 872 -0.070 -0.607 -0.314

1499 2400 901 -0.070 -0.607 -0.314

1499 2453 954 -0.070 -0.607 -0.314

1499 2473 974 -0.017 -1.000 -0.165

1499 2493 994 -0.017 -1.000 -0.165

1499 2570 1071 -0.017 -1.000 -0.165

1499 2598 1099 -0.070 -0.607 -0.314

1499 2608 1109 -0.070 -0.607 -0.314

1499 2613 1114 -0.070 -0.607 -0.314

1499 2688 1189 -0.054 -0.542 -0.251

1499 2889 1390 -0.017 -1.000 -0.165

1499 2890 1391 -0.017 -1.000 -0.165

1499 2913 1414 -0.070 -0.607 -0.314

1499 2943 1444 -0.070 -0.607 -0.314

1499 3384 1885 -0.054 -0.542 -0.251

1499 3553 2054 -0.037 -0.450 -0.184

1499 3605 2106 0.095 0.411 0.411

1499 3806 2307 -0.050 -1.000 -0.300

1499 3859 2360 -0.054 -0.542 -0.251

1499 3935 2436 -0.037 -0.450 -0.184

1499 4121 2622 -0.037 -0.450 -0.184

1499 4129 2630 -0.004 -0.083 -0.025

1499 4137 2638 -0.037 -0.450 -0.184

1499 4139 2640 0.107 0.542 0.449

1499 4248 2749 -0.037 -0.450 -0.184

1499 4283 2784 -0.033 -1.000 -0.239

1499 4400 2901 -0.033 -1.000 -0.239

1510 1602 92 0.058 1.000 0.418

1510 1623 113 -0.004 -1.000 -0.069

1510 1786 276 -0.041 -1.000 -0.289

1510 1804 294 0.058 1.000 0.418

1510 1807 297 0.054 1.000 0.380

1510 1963 453 0.054 1.000 0.380

1510 1974 464 0.058 1.000 0.418

1510 2027 517 -0.004 -1.000 -0.069

1510 2122 612 0.054 1.000 0.380

1510 2340 830 -0.004 -1.000 -0.069

1510 2344 834 -0.004 -1.000 -0.069

1510 2370 860 -0.012 -1.000 -0.126

1510 2371 861 -0.029 -1.000 -0.216

1510 2400 890 -0.029 -1.000 -0.216

1510 2453 943 -0.029 -1.000 -0.216

1510 2473 963 -0.004 -1.000 -0.069

1510 2493 983 -0.004 -1.000 -0.069

1510 2570 1060 -0.004 -1.000 -0.069

1510 2598 1088 -0.029 -1.000 -0.216

1510 2608 1098 -0.029 -1.000 -0.216

1510 2613 1103 -0.029 -1.000 -0.216

1510 2688 1178 -0.025 -1.000 -0.194

1510 2889 1379 -0.004 -1.000 -0.069

1510 2890 1380 -0.004 -1.000 -0.069

1510 2913 1403 -0.029 -1.000 -0.216

1510 2943 1433 -0.029 -1.000 -0.216

1510 3384 1874 -0.025 -1.000 -0.194

1510 3553 2043 -0.021 -1.000 -0.171

1510 3605 2095 0.058 1.000 0.418

1510 3806 2296 -0.012 -1.000 -0.126

1510 3859 2349 -0.025 -1.000 -0.194

1510 3935 2425 -0.021 -1.000 -0.171

1510 4121 2611 -0.021 -1.000 -0.171

1510 4129 2619 -0.012 -1.000 -0.126

1510 4137 2627 -0.021 -1.000 -0.171

1510 4139 2629 0.050 1.000 0.346

1510 4248 2738 -0.021 -1.000 -0.171

1510 4283 2773 -0.008 -1.000 -0.100

1510 4400 2890 -0.008 -1.000 -0.100

1602 1623 21 -0.017 -1.000 -0.165

1602 1786 184 -0.165 -1.000 -0.690

1602 1804 202 0.095 0.411 0.411

1602 1807 205 0.124 0.577 0.524

1602 1963 361 0.124 0.577 0.524

1602 1974 372 0.095 0.411 0.411

1602 2027 425 -0.017 -1.000 -0.165

1602 2122 520 0.079 0.365 0.332

1602 2340 738 -0.017 -1.000 -0.165

1602 2344 742 -0.017 -1.000 -0.165

1602 2370 768 -0.050 -1.000 -0.300

1602 2371 769 -0.116 -1.000 -0.516

1602 2400 798 -0.116 -1.000 -0.516

1602 2453 851 -0.116 -1.000 -0.516

1602 2473 871 -0.017 -1.000 -0.165

1602 2493 891 -0.017 -1.000 -0.165

1602 2570 968 -0.017 -1.000 -0.165

1602 2598 996 -0.116 -1.000 -0.516

1602 2608 1006 -0.116 -1.000 -0.516

1602 2613 1011 -0.116 -1.000 -0.516

1602 2688 1086 -0.099 -1.000 -0.463

1602 2889 1287 -0.017 -1.000 -0.165

1602 2890 1288 -0.017 -1.000 -0.165

1602 2913 1311 -0.116 -1.000 -0.516

1602 2943 1341 -0.116 -1.000 -0.516

1602 3384 1782 -0.099 -1.000 -0.463

1602 3553 1951 -0.083 -1.000 -0.410

1602 3605 2003 0.095 0.411 0.411

1602 3806 2204 -0.050 -1.000 -0.300

1602 3859 2257 -0.099 -1.000 -0.463

1602 3935 2333 -0.083 -1.000 -0.410

1602 4121 2519 -0.083 -1.000 -0.410

1602 4129 2527 -0.050 -1.000 -0.300

1602 4137 2535 -0.083 -1.000 -0.410

1602 4139 2537 0.107 0.542 0.449

1602 4248 2646 -0.083 -1.000 -0.410

1602 4283 2681 -0.033 -1.000 -0.239

1602 4400 2798 -0.033 -1.000 -0.239

1623 1786 163 -0.021 -1.000 -0.199

1623 1804 181 -0.017 -1.000 -0.165

1623 1807 184 -0.019 -1.000 -0.182

1623 1963 340 -0.019 -1.000 -0.182

1623 1974 351 -0.017 -1.000 -0.165

1623 2027 404 -0.002 -1.000 -0.048

1623 2122 499 0.027 1.000 0.262

1623 2340 717 0.043 1.000 1.000

1623 2344 721 0.043 1.000 1.000

1623 2370 747 0.039 1.000 0.549

1623 2371 748 0.031 1.000 0.319

1623 2400 777 0.031 1.000 0.319

1623 2453 830 0.031 1.000 0.319

1623 2473 850 0.043 1.000 1.000

1623 2493 870 0.043 1.000 1.000

1623 2570 947 0.043 1.000 1.000

1623 2598 975 0.031 1.000 0.319

1623 2608 985 0.031 1.000 0.319

1623 2613 990 0.031 1.000 0.319

1623 2688 1065 0.033 1.000 0.356

1623 2889 1266 -0.002 -1.000 -0.048

1623 2890 1267 -0.002 -1.000 -0.048

1623 2913 1290 0.031 1.000 0.319

1623 2943 1320 0.031 1.000 0.319

1623 3384 1761 0.033 1.000 0.356

1623 3553 1930 0.035 1.000 0.402

1623 3605 1982 -0.017 -1.000 -0.165

1623 3806 2183 0.039 1.000 0.549

1623 3859 2236 0.033 1.000 0.356

1623 3935 2312 -0.010 -1.000 -0.118

1623 4121 2498 -0.010 -1.000 -0.118

1623 4129 2506 -0.006 -1.000 -0.087

1623 4137 2514 -0.010 -1.000 -0.118

1623 4139 2516 0.025 1.000 0.239

1623 4248 2625 -0.010 -1.000 -0.118

1623 4283 2660 -0.004 -1.000 -0.069

1623 4400 2777 -0.004 -1.000 -0.069

1786 1804 18 -0.165 -1.000 -0.690

1786 1807 21 -0.186 -1.000 -0.760

1786 1963 177 -0.186 -1.000 -0.760

1786 1974 188 -0.165 -1.000 -0.690

1786 2027 241 0.025 1.000 0.239

1786 2122 336 -0.186 -1.000 -0.760

1786 2340 554 -0.021 -1.000 -0.199

1786 2344 558 -0.021 -1.000 -0.199

1786 2370 584 0.029 0.389 0.169

1786 2371 585 0.128 0.738 0.552

1786 2400 614 0.128 0.738 0.552

1786 2453 667 0.128 0.738 0.552

1786 2473 687 -0.021 -1.000 -0.199

1786 2493 707 -0.021 -1.000 -0.199

1786 2570 784 -0.021 -1.000 -0.199

1786 2598 812 0.128 0.738 0.552

1786 2608 822 0.128 0.738 0.552

1786 2613 827 0.128 0.738 0.552

1786 2688 902 0.103 0.694 0.466

1786 2889 1103 0.025 1.000 0.239

1786 2890 1104 0.025 1.000 0.239

1786 2913 1127 0.128 0.738 0.552

1786 2943 1157 0.128 0.738 0.552

1786 3384 1598 0.103 0.694 0.466

1786 3553 1767 0.079 0.633 0.376

1786 3605 1819 -0.165 -1.000 -0.690

1786 3806 2020 0.029 0.389 0.169

1786 3859 2073 0.103 0.694 0.466

1786 3935 2149 0.124 1.000 0.594

1786 4121 2335 0.124 1.000 0.594

1786 4129 2343 0.074 1.000 0.435

1786 4137 2351 0.124 1.000 0.594

1786 4139 2353 -0.207 -1.000 -0.833

1786 4248 2462 0.124 1.000 0.594

1786 4283 2497 0.050 1.000 0.346

1786 4400 2614 0.050 1.000 0.346

1804 1807 3 0.215 1.000 0.909

1804 1963 159 0.215 1.000 0.909

1804 1974 170 0.231 1.000 1.000

1804 2027 223 -0.017 -1.000 -0.165

1804 2122 318 0.215 1.000 0.909

1804 2340 536 -0.017 -1.000 -0.165

1804 2344 540 -0.017 -1.000 -0.165

1804 2370 566 -0.050 -1.000 -0.300

1804 2371 567 -0.116 -1.000 -0.516

1804 2400 596 -0.116 -1.000 -0.516

1804 2453 649 -0.116 -1.000 -0.516

1804 2473 669 -0.017 -1.000 -0.165

1804 2493 689 -0.017 -1.000 -0.165

1804 2570 766 -0.017 -1.000 -0.165

1804 2598 794 -0.116 -1.000 -0.516

1804 2608 804 -0.116 -1.000 -0.516

1804 2613 809 -0.116 -1.000 -0.516

1804 2688 884 -0.099 -1.000 -0.463

1804 2889 1085 -0.017 -1.000 -0.165

1804 2890 1086 -0.017 -1.000 -0.165

1804 2913 1109 -0.116 -1.000 -0.516

1804 2943 1139 -0.116 -1.000 -0.516

1804 3384 1580 -0.099 -1.000 -0.463

1804 3553 1749 -0.083 -1.000 -0.410

1804 3605 1801 0.231 1.000 1.000

1804 3806 2002 -0.050 -1.000 -0.300

1804 3859 2055 -0.099 -1.000 -0.463

1804 3935 2131 -0.083 -1.000 -0.410

1804 4121 2317 -0.083 -1.000 -0.410

1804 4129 2325 -0.050 -1.000 -0.300

1804 4137 2333 -0.083 -1.000 -0.410

1804 4139 2335 0.198 1.000 0.828

1804 4248 2444 -0.083 -1.000 -0.410

1804 4283 2479 -0.033 -1.000 -0.239

1804 4400 2596 -0.033 -1.000 -0.239

1807 1963 156 0.242 1.000 1.000

1807 1974 167 0.215 1.000 0.909

1807 2027 220 -0.019 -1.000 -0.182

1807 2122 315 0.196 0.812 0.812

1807 2340 533 -0.019 -1.000 -0.182

1807 2344 537 -0.019 -1.000 -0.182

1807 2370 563 -0.056 -1.000 -0.331

1807 2371 564 -0.130 -1.000 -0.568

1807 2400 593 -0.130 -1.000 -0.568

1807 2453 646 -0.130 -1.000 -0.568

1807 2473 666 -0.019 -1.000 -0.182

1807 2493 686 -0.019 -1.000 -0.182

1807 2570 763 -0.019 -1.000 -0.182

1807 2598 791 -0.130 -1.000 -0.568

1807 2608 801 -0.130 -1.000 -0.568

1807 2613 806 -0.130 -1.000 -0.568

1807 2688 881 -0.112 -1.000 -0.510

1807 2889 1082 -0.019 -1.000 -0.182

1807 2890 1083 -0.019 -1.000 -0.182

1807 2913 1106 -0.130 -1.000 -0.568

1807 2943 1136 -0.130 -1.000 -0.568

1807 3384 1577 -0.112 -1.000 -0.510

1807 3553 1746 -0.093 -1.000 -0.451

1807 3605 1798 0.215 1.000 0.909

1807 3806 1999 -0.056 -1.000 -0.331

1807 3859 2052 -0.112 -1.000 -0.510

1807 3935 2128 -0.093 -1.000 -0.451

1807 4121 2314 -0.093 -1.000 -0.451

1807 4129 2322 -0.056 -1.000 -0.331

1807 4137 2330 -0.093 -1.000 -0.451

1807 4139 2332 0.223 1.000 0.911

1807 4248 2441 -0.093 -1.000 -0.451

1807 4283 2476 -0.037 -1.000 -0.263

1807 4400 2593 -0.037 -1.000 -0.263

1963 1974 11 0.215 1.000 0.909

1963 2027 64 -0.019 -1.000 -0.182

1963 2122 159 0.196 0.812 0.812

1963 2340 377 -0.019 -1.000 -0.182

1963 2344 381 -0.019 -1.000 -0.182

1963 2370 407 -0.056 -1.000 -0.331

1963 2371 408 -0.130 -1.000 -0.568

1963 2400 437 -0.130 -1.000 -0.568

1963 2453 490 -0.130 -1.000 -0.568

1963 2473 510 -0.019 -1.000 -0.182

1963 2493 530 -0.019 -1.000 -0.182

1963 2570 607 -0.019 -1.000 -0.182

1963 2598 635 -0.130 -1.000 -0.568

1963 2608 645 -0.130 -1.000 -0.568

1963 2613 650 -0.130 -1.000 -0.568

1963 2688 725 -0.112 -1.000 -0.510

1963 2889 926 -0.019 -1.000 -0.182

1963 2890 927 -0.019 -1.000 -0.182

1963 2913 950 -0.130 -1.000 -0.568

1963 2943 980 -0.130 -1.000 -0.568

1963 3384 1421 -0.112 -1.000 -0.510

1963 3553 1590 -0.093 -1.000 -0.451

1963 3605 1642 0.215 1.000 0.909

1963 3806 1843 -0.056 -1.000 -0.331

1963 3859 1896 -0.112 -1.000 -0.510

1963 3935 1972 -0.093 -1.000 -0.451

1963 4121 2158 -0.093 -1.000 -0.451

1963 4129 2166 -0.056 -1.000 -0.331

1963 4137 2174 -0.093 -1.000 -0.451

1963 4139 2176 0.223 1.000 0.911

1963 4248 2285 -0.093 -1.000 -0.451

1963 4283 2320 -0.037 -1.000 -0.263

1963 4400 2437 -0.037 -1.000 -0.263

1974 2027 53 -0.017 -1.000 -0.165

1974 2122 148 0.215 1.000 0.909

1974 2340 366 -0.017 -1.000 -0.165

1974 2344 370 -0.017 -1.000 -0.165

1974 2370 396 -0.050 -1.000 -0.300

1974 2371 397 -0.116 -1.000 -0.516

1974 2400 426 -0.116 -1.000 -0.516

1974 2453 479 -0.116 -1.000 -0.516

1974 2473 499 -0.017 -1.000 -0.165

1974 2493 519 -0.017 -1.000 -0.165

1974 2570 596 -0.017 -1.000 -0.165

1974 2598 624 -0.116 -1.000 -0.516

1974 2608 634 -0.116 -1.000 -0.516

1974 2613 639 -0.116 -1.000 -0.516

1974 2688 714 -0.099 -1.000 -0.463

1974 2889 915 -0.017 -1.000 -0.165

1974 2890 916 -0.017 -1.000 -0.165

1974 2913 939 -0.116 -1.000 -0.516

1974 2943 969 -0.116 -1.000 -0.516

1974 3384 1410 -0.099 -1.000 -0.463

1974 3553 1579 -0.083 -1.000 -0.410

1974 3605 1631 0.231 1.000 1.000

1974 3806 1832 -0.050 -1.000 -0.300

1974 3859 1885 -0.099 -1.000 -0.463

1974 3935 1961 -0.083 -1.000 -0.410

1974 4121 2147 -0.083 -1.000 -0.410

1974 4129 2155 -0.050 -1.000 -0.300

1974 4137 2163 -0.083 -1.000 -0.410

1974 4139 2165 0.198 1.000 0.828

1974 4248 2274 -0.083 -1.000 -0.410

1974 4283 2309 -0.033 -1.000 -0.239

1974 4400 2426 -0.033 -1.000 -0.239

2027 2122 95 -0.019 -1.000 -0.182

2027 2340 313 -0.002 -1.000 -0.048

2027 2344 317 -0.002 -1.000 -0.048

2027 2370 343 -0.006 -1.000 -0.087

2027 2371 344 0.031 1.000 0.319

2027 2400 373 0.031 1.000 0.319

2027 2453 426 0.031 1.000 0.319

2027 2473 446 -0.002 -1.000 -0.048

2027 2493 466 -0.002 -1.000 -0.048

2027 2570 543 -0.002 -1.000 -0.048

2027 2598 571 0.031 1.000 0.319

2027 2608 581 0.031 1.000 0.319

2027 2613 586 0.031 1.000 0.319

2027 2688 661 -0.012 -1.000 -0.134

2027 2889 862 0.043 1.000 1.000

2027 2890 863 0.043 1.000 1.000

2027 2913 886 0.031 1.000 0.319

2027 2943 916 0.031 1.000 0.319

2027 3384 1357 0.033 1.000 0.356

2027 3553 1526 0.035 1.000 0.402

2027 3605 1578 -0.017 -1.000 -0.165

2027 3806 1779 0.039 1.000 0.549

2027 3859 1832 0.033 1.000 0.356

2027 3935 1908 0.035 1.000 0.402

2027 4121 2094 0.035 1.000 0.402

2027 4129 2102 0.039 1.000 0.549

2027 4137 2110 0.035 1.000 0.402

2027 4139 2112 -0.021 -1.000 -0.199

2027 4248 2221 0.035 1.000 0.402

2027 4283 2256 -0.004 -1.000 -0.069

2027 4400 2373 -0.004 -1.000 -0.069

2122 2340 218 0.027 1.000 0.262

2122 2344 222 0.027 1.000 0.262

2122 2370 248 -0.010 -0.185 -0.061

2122 2371 249 -0.085 -0.651 -0.370

2122 2400 278 -0.085 -0.651 -0.370

2122 2453 331 -0.085 -0.651 -0.370

2122 2473 351 0.027 1.000 0.262

2122 2493 371 0.027 1.000 0.262

2122 2570 448 0.027 1.000 0.262

2122 2598 476 -0.085 -0.651 -0.370

2122 2608 486 -0.085 -0.651 -0.370

2122 2613 491 -0.085 -0.651 -0.370

2122 2688 566 -0.066 -0.593 -0.302

2122 2889 767 -0.019 -1.000 -0.182

2122 2890 768 -0.019 -1.000 -0.182

2122 2913 791 -0.085 -0.651 -0.370

2122 2943 821 -0.085 -0.651 -0.370

2122 3384 1262 -0.066 -0.593 -0.302

2122 3553 1431 -0.048 -0.511 -0.231

2122 3605 1483 0.215 1.000 0.909

2122 3806 1684 -0.010 -0.185 -0.061

2122 3859 1737 -0.066 -0.593 -0.302

2122 3935 1813 -0.093 -1.000 -0.451

2122 4121 1999 -0.093 -1.000 -0.451

2122 4129 2007 -0.056 -1.000 -0.331

2122 4137 2015 -0.093 -1.000 -0.451

2122 4139 2017 0.223 1.000 0.911

2122 4248 2126 -0.093 -1.000 -0.451

2122 4283 2161 -0.037 -1.000 -0.263

2122 4400 2278 -0.037 -1.000 -0.263

2340 2344 4 0.043 1.000 1.000

2340 2370 30 0.039 1.000 0.549

2340 2371 31 0.031 1.000 0.319

2340 2400 60 0.031 1.000 0.319

2340 2453 113 0.031 1.000 0.319

2340 2473 133 0.043 1.000 1.000

2340 2493 153 0.043 1.000 1.000

2340 2570 230 0.043 1.000 1.000

2340 2598 258 0.031 1.000 0.319

2340 2608 268 0.031 1.000 0.319

2340 2613 273 0.031 1.000 0.319

2340 2688 348 0.033 1.000 0.356

2340 2889 549 -0.002 -1.000 -0.048

2340 2890 550 -0.002 -1.000 -0.048

2340 2913 573 0.031 1.000 0.319

2340 2943 603 0.031 1.000 0.319

2340 3384 1044 0.033 1.000 0.356

2340 3553 1213 0.035 1.000 0.402

2340 3605 1265 -0.017 -1.000 -0.165

2340 3806 1466 0.039 1.000 0.549

2340 3859 1519 0.033 1.000 0.356

2340 3935 1595 -0.010 -1.000 -0.118

2340 4121 1781 -0.010 -1.000 -0.118

2340 4129 1789 -0.006 -1.000 -0.087

2340 4137 1797 -0.010 -1.000 -0.118

2340 4139 1799 0.025 1.000 0.239

2340 4248 1908 -0.010 -1.000 -0.118

2340 4283 1943 -0.004 -1.000 -0.069

2340 4400 2060 -0.004 -1.000 -0.069

2344 2370 26 0.039 1.000 0.549

2344 2371 27 0.031 1.000 0.319

2344 2400 56 0.031 1.000 0.319

2344 2453 109 0.031 1.000 0.319

2344 2473 129 0.043 1.000 1.000

2344 2493 149 0.043 1.000 1.000

2344 2570 226 0.043 1.000 1.000

2344 2598 254 0.031 1.000 0.319

2344 2608 264 0.031 1.000 0.319

2344 2613 269 0.031 1.000 0.319

2344 2688 344 0.033 1.000 0.356

2344 2889 545 -0.002 -1.000 -0.048

2344 2890 546 -0.002 -1.000 -0.048

2344 2913 569 0.031 1.000 0.319

2344 2943 599 0.031 1.000 0.319

2344 3384 1040 0.033 1.000 0.356

2344 3553 1209 0.035 1.000 0.402

2344 3605 1261 -0.017 -1.000 -0.165

2344 3806 1462 0.039 1.000 0.549

2344 3859 1515 0.033 1.000 0.356

2344 3935 1591 -0.010 -1.000 -0.118

2344 4121 1777 -0.010 -1.000 -0.118

2344 4129 1785 -0.006 -1.000 -0.087

2344 4137 1793 -0.010 -1.000 -0.118

2344 4139 1795 0.025 1.000 0.239

2344 4248 1904 -0.010 -1.000 -0.118

2344 4283 1939 -0.004 -1.000 -0.069

2344 4400 2056 -0.004 -1.000 -0.069

2370 2371 1 0.093 1.000 0.582

2370 2400 30 0.093 1.000 0.582

2370 2453 83 0.093 1.000 0.582

2370 2473 103 0.039 1.000 0.549

2370 2493 123 0.039 1.000 0.549

2370 2570 200 0.039 1.000 0.549

2370 2598 228 0.093 1.000 0.582

2370 2608 238 0.093 1.000 0.582

2370 2613 243 0.093 1.000 0.582

2370 2688 318 0.099 1.000 0.649

2370 2889 519 -0.006 -1.000 -0.087

2370 2890 520 -0.006 -1.000 -0.087

2370 2913 543 0.093 1.000 0.582

2370 2943 573 0.093 1.000 0.582

2370 3384 1014 0.099 1.000 0.649

2370 3553 1183 0.060 0.569 0.417

2370 3605 1235 -0.050 -1.000 -0.300

2370 3806 1436 0.072 0.614 0.614

2370 3859 1489 0.099 1.000 0.649

2370 3935 1565 0.060 0.569 0.417

2370 4121 1751 0.060 0.569 0.417

2370 4129 1759 0.072 0.614 0.614

2370 4137 1767 0.060 0.569 0.417

2370 4139 1769 -0.017 -0.267 -0.097

2370 4248 1878 0.060 0.569 0.417

2370 4283 1913 -0.012 -1.000 -0.126

2370 4400 2030 -0.012 -1.000 -0.126

2371 2400 29 0.217 1.000 1.000

2371 2453 82 0.217 1.000 1.000

2371 2473 102 0.031 1.000 0.319

2371 2493 122 0.031 1.000 0.319

2371 2570 199 0.031 1.000 0.319

2371 2598 227 0.217 1.000 1.000

2371 2608 237 0.217 1.000 1.000

2371 2613 242 0.217 1.000 1.000

2371 2688 317 0.186 1.000 0.896

2371 2889 518 0.031 1.000 0.319

2371 2890 519 0.031 1.000 0.319

2371 2913 542 0.217 1.000 1.000

2371 2943 572 0.217 1.000 1.000

2371 3384 1013 0.186 1.000 0.896

2371 3553 1182 0.155 1.000 0.794

2371 3605 1234 -0.116 -1.000 -0.516

2371 3806 1435 0.093 1.000 0.582

2371 3859 1488 0.186 1.000 0.896

2371 3935 1564 0.155 1.000 0.794

2371 4121 1750 0.155 1.000 0.794

2371 4129 1758 0.093 1.000 0.582

2371 4137 1766 0.155 1.000 0.794

2371 4139 1768 -0.099 -0.686 -0.428

2371 4248 1877 0.155 1.000 0.794

2371 4283 1912 -0.029 -1.000 -0.216

2371 4400 2029 0.062 1.000 0.463

2400 2453 53 0.217 1.000 1.000

2400 2473 73 0.031 1.000 0.319

2400 2493 93 0.031 1.000 0.319

2400 2570 170 0.031 1.000 0.319

2400 2598 198 0.217 1.000 1.000

2400 2608 208 0.217 1.000 1.000

2400 2613 213 0.217 1.000 1.000

2400 2688 288 0.186 1.000 0.896

2400 2889 489 0.031 1.000 0.319

2400 2890 490 0.031 1.000 0.319

2400 2913 513 0.217 1.000 1.000

2400 2943 543 0.217 1.000 1.000

2400 3384 984 0.186 1.000 0.896

2400 3553 1153 0.155 1.000 0.794

2400 3605 1205 -0.116 -1.000 -0.516

2400 3806 1406 0.093 1.000 0.582

2400 3859 1459 0.186 1.000 0.896

2400 3935 1535 0.155 1.000 0.794

2400 4121 1721 0.155 1.000 0.794

2400 4129 1729 0.093 1.000 0.582

2400 4137 1737 0.155 1.000 0.794

2400 4139 1739 -0.099 -0.686 -0.428

2400 4248 1848 0.155 1.000 0.794

2400 4283 1883 -0.029 -1.000 -0.216

2400 4400 2000 0.062 1.000 0.463

2453 2473 20 0.031 1.000 0.319

2453 2493 40 0.031 1.000 0.319

2453 2570 117 0.031 1.000 0.319

2453 2598 145 0.217 1.000 1.000

2453 2608 155 0.217 1.000 1.000

2453 2613 160 0.217 1.000 1.000

2453 2688 235 0.186 1.000 0.896

2453 2889 436 0.031 1.000 0.319

2453 2890 437 0.031 1.000 0.319

2453 2913 460 0.217 1.000 1.000

2453 2943 490 0.217 1.000 1.000

2453 3384 931 0.186 1.000 0.896

2453 3553 1100 0.155 1.000 0.794

2453 3605 1152 -0.116 -1.000 -0.516

2453 3806 1353 0.093 1.000 0.582

2453 3859 1406 0.186 1.000 0.896

2453 3935 1482 0.155 1.000 0.794

2453 4121 1668 0.155 1.000 0.794

2453 4129 1676 0.093 1.000 0.582

2453 4137 1684 0.155 1.000 0.794

2453 4139 1686 -0.099 -0.686 -0.428

2453 4248 1795 0.155 1.000 0.794

2453 4283 1830 -0.029 -1.000 -0.216

2453 4400 1947 0.062 1.000 0.463

2473 2493 20 0.043 1.000 1.000

2473 2570 97 0.043 1.000 1.000

2473 2598 125 0.031 1.000 0.319

2473 2608 135 0.031 1.000 0.319

2473 2613 140 0.031 1.000 0.319

2473 2688 215 0.033 1.000 0.356

2473 2889 416 -0.002 -1.000 -0.048

2473 2890 417 -0.002 -1.000 -0.048

2473 2913 440 0.031 1.000 0.319

2473 2943 470 0.031 1.000 0.319

2473 3384 911 0.033 1.000 0.356

2473 3553 1080 0.035 1.000 0.402

2473 3605 1132 -0.017 -1.000 -0.165

2473 3806 1333 0.039 1.000 0.549

2473 3859 1386 0.033 1.000 0.356

2473 3935 1462 -0.010 -1.000 -0.118

2473 4121 1648 -0.010 -1.000 -0.118

2473 4129 1656 -0.006 -1.000 -0.087

2473 4137 1664 -0.010 -1.000 -0.118

2473 4139 1666 0.025 1.000 0.239

2473 4248 1775 -0.010 -1.000 -0.118

2473 4283 1810 -0.004 -1.000 -0.069

2473 4400 1927 -0.004 -1.000 -0.069

2493 2570 77 0.043 1.000 1.000

2493 2598 105 0.031 1.000 0.319

2493 2608 115 0.031 1.000 0.319

2493 2613 120 0.031 1.000 0.319

2493 2688 195 0.033 1.000 0.356

2493 2889 396 -0.002 -1.000 -0.048

2493 2890 397 -0.002 -1.000 -0.048

2493 2913 420 0.031 1.000 0.319

2493 2943 450 0.031 1.000 0.319

2493 3384 891 0.033 1.000 0.356

2493 3553 1060 0.035 1.000 0.402

2493 3605 1112 -0.017 -1.000 -0.165

2493 3806 1313 0.039 1.000 0.549

2493 3859 1366 0.033 1.000 0.356

2493 3935 1442 -0.010 -1.000 -0.118

2493 4121 1628 -0.010 -1.000 -0.118

2493 4129 1636 -0.006 -1.000 -0.087

2493 4137 1644 -0.010 -1.000 -0.118

2493 4139 1646 0.025 1.000 0.239

2493 4248 1755 -0.010 -1.000 -0.118

2493 4283 1790 -0.004 -1.000 -0.069

2493 4400 1907 -0.004 -1.000 -0.069

2570 2598 28 0.031 1.000 0.319

2570 2608 38 0.031 1.000 0.319

2570 2613 43 0.031 1.000 0.319

2570 2688 118 0.033 1.000 0.356

2570 2889 319 -0.002 -1.000 -0.048

2570 2890 320 -0.002 -1.000 -0.048

2570 2913 343 0.031 1.000 0.319

2570 2943 373 0.031 1.000 0.319

2570 3384 814 0.033 1.000 0.356

2570 3553 983 0.035 1.000 0.402

2570 3605 1035 -0.017 -1.000 -0.165

2570 3806 1236 0.039 1.000 0.549

2570 3859 1289 0.033 1.000 0.356

2570 3935 1365 -0.010 -1.000 -0.118

2570 4121 1551 -0.010 -1.000 -0.118

2570 4129 1559 -0.006 -1.000 -0.087

2570 4137 1567 -0.010 -1.000 -0.118

2570 4139 1569 0.025 1.000 0.239

2570 4248 1678 -0.010 -1.000 -0.118

2570 4283 1713 -0.004 -1.000 -0.069

2570 4400 1830 -0.004 -1.000 -0.069

2598 2608 10 0.217 1.000 1.000

2598 2613 15 0.217 1.000 1.000

2598 2688 90 0.186 1.000 0.896

2598 2889 291 0.031 1.000 0.319

2598 2890 292 0.031 1.000 0.319

2598 2913 315 0.217 1.000 1.000

2598 2943 345 0.217 1.000 1.000

2598 3384 786 0.186 1.000 0.896

2598 3553 955 0.155 1.000 0.794

2598 3605 1007 -0.116 -1.000 -0.516

2598 3806 1208 0.093 1.000 0.582

2598 3859 1261 0.186 1.000 0.896

2598 3935 1337 0.155 1.000 0.794

2598 4121 1523 0.155 1.000 0.794

2598 4129 1531 0.093 1.000 0.582

2598 4137 1539 0.155 1.000 0.794

2598 4139 1541 -0.099 -0.686 -0.428

2598 4248 1650 0.155 1.000 0.794

2598 4283 1685 -0.029 -1.000 -0.216

2598 4400 1802 0.062 1.000 0.463

2608 2613 5 0.217 1.000 1.000

2608 2688 80 0.186 1.000 0.896

2608 2889 281 0.031 1.000 0.319

2608 2890 282 0.031 1.000 0.319

2608 2913 305 0.217 1.000 1.000

2608 2943 335 0.217 1.000 1.000

2608 3384 776 0.186 1.000 0.896

2608 3553 945 0.155 1.000 0.794

2608 3605 997 -0.116 -1.000 -0.516

2608 3806 1198 0.093 1.000 0.582

2608 3859 1251 0.186 1.000 0.896

2608 3935 1327 0.155 1.000 0.794

2608 4121 1513 0.155 1.000 0.794

2608 4129 1521 0.093 1.000 0.582

2608 4137 1529 0.155 1.000 0.794

2608 4139 1531 -0.099 -0.686 -0.428

2608 4248 1640 0.155 1.000 0.794

2608 4283 1675 -0.029 -1.000 -0.216

2608 4400 1792 0.062 1.000 0.463

2613 2688 75 0.186 1.000 0.896

2613 2889 276 0.031 1.000 0.319

2613 2890 277 0.031 1.000 0.319

2613 2913 300 0.217 1.000 1.000

2613 2943 330 0.217 1.000 1.000

2613 3384 771 0.186 1.000 0.896

2613 3553 940 0.155 1.000 0.794

2613 3605 992 -0.116 -1.000 -0.516

2613 3806 1193 0.093 1.000 0.582

2613 3859 1246 0.186 1.000 0.896

2613 3935 1322 0.155 1.000 0.794

2613 4121 1508 0.155 1.000 0.794

2613 4129 1516 0.093 1.000 0.582

2613 4137 1524 0.155 1.000 0.794

2613 4139 1526 -0.099 -0.686 -0.428

2613 4248 1635 0.155 1.000 0.794

2613 4283 1670 -0.029 -1.000 -0.216

2613 4400 1787 0.062 1.000 0.463

2688 2889 201 -0.012 -1.000 -0.134

2688 2890 202 -0.012 -1.000 -0.134

2688 2913 225 0.186 1.000 0.896

2688 2943 255 0.186 1.000 0.896

2688 3384 696 0.153 0.771 0.771

2688 3553 865 0.120 0.725 0.642

2688 3605 917 -0.099 -1.000 -0.463

2688 3806 1118 0.054 0.542 0.351

2688 3859 1171 0.153 0.771 0.771

2688 3935 1247 0.120 0.725 0.642

2688 4121 1433 0.120 0.725 0.642

2688 4129 1441 0.054 0.542 0.351

2688 4137 1449 0.120 0.725 0.642

2688 4139 1451 -0.079 -0.633 -0.354

2688 4248 1560 0.120 0.725 0.642

2688 4283 1595 -0.025 -1.000 -0.194

2688 4400 1712 0.066 1.000 0.516

2889 2890 1 0.043 1.000 1.000

2889 2913 24 0.031 1.000 0.319

2889 2943 54 0.031 1.000 0.319

2889 3384 495 0.033 1.000 0.356

2889 3553 664 0.035 1.000 0.402

2889 3605 716 -0.017 -1.000 -0.165

2889 3806 917 0.039 1.000 0.549

2889 3859 970 0.033 1.000 0.356

2889 3935 1046 0.035 1.000 0.402

2889 4121 1232 0.035 1.000 0.402

2889 4129 1240 0.039 1.000 0.549

2889 4137 1248 0.035 1.000 0.402

2889 4139 1250 -0.021 -1.000 -0.199

2889 4248 1359 0.035 1.000 0.402

2889 4283 1394 -0.004 -1.000 -0.069

2889 4400 1511 -0.004 -1.000 -0.069

2890 2913 23 0.031 1.000 0.319

2890 2943 53 0.031 1.000 0.319

2890 3384 494 0.033 1.000 0.356

2890 3553 663 0.035 1.000 0.402

2890 3605 715 -0.017 -1.000 -0.165

2890 3806 916 0.039 1.000 0.549

2890 3859 969 0.033 1.000 0.356

2890 3935 1045 0.035 1.000 0.402

2890 4121 1231 0.035 1.000 0.402

2890 4129 1239 0.039 1.000 0.549

2890 4137 1247 0.035 1.000 0.402

2890 4139 1249 -0.021 -1.000 -0.199

2890 4248 1358 0.035 1.000 0.402

2890 4283 1393 -0.004 -1.000 -0.069

2890 4400 1510 -0.004 -1.000 -0.069

2913 2943 30 0.217 1.000 1.000

2913 3384 471 0.186 1.000 0.896

2913 3553 640 0.155 1.000 0.794

2913 3605 692 -0.116 -1.000 -0.516

2913 3806 893 0.093 1.000 0.582

2913 3859 946 0.186 1.000 0.896

2913 3935 1022 0.155 1.000 0.794

2913 4121 1208 0.155 1.000 0.794

2913 4129 1216 0.093 1.000 0.582

2913 4137 1224 0.155 1.000 0.794

2913 4139 1226 -0.099 -0.686 -0.428

2913 4248 1335 0.155 1.000 0.794

2913 4283 1370 -0.029 -1.000 -0.216

2913 4400 1487 0.062 1.000 0.463

2943 3384 441 0.186 1.000 0.896

2943 3553 610 0.155 1.000 0.794

2943 3605 662 -0.116 -1.000 -0.516

2943 3806 863 0.093 1.000 0.582

2943 3859 916 0.186 1.000 0.896

2943 3935 992 0.155 1.000 0.794

2943 4121 1178 0.155 1.000 0.794

2943 4129 1186 0.093 1.000 0.582

2943 4137 1194 0.155 1.000 0.794

2943 4139 1196 -0.099 -0.686 -0.428

2943 4248 1305 0.155 1.000 0.794

2943 4283 1340 -0.029 -1.000 -0.216

2943 4400 1457 0.062 1.000 0.463

3384 3553 169 0.165 1.000 0.886

3384 3605 221 -0.099 -1.000 -0.463

3384 3806 422 0.099 1.000 0.649

3384 3859 475 0.198 1.000 1.000

3384 3935 551 0.165 1.000 0.886

3384 4121 737 0.165 1.000 0.886

3384 4129 745 0.099 1.000 0.649

3384 4137 753 0.165 1.000 0.886

3384 4139 755 -0.079 -0.633 -0.354

3384 4248 864 0.165 1.000 0.886

3384 4283 899 -0.025 -1.000 -0.194

3384 4400 1016 0.066 1.000 0.516

3553 3605 52 -0.083 -1.000 -0.410

3553 3806 253 0.060 0.569 0.417

3553 3859 306 0.165 1.000 0.886

3553 3935 382 0.130 0.741 0.741

3553 4121 568 0.130 0.741 0.741

3553 4129 576 0.060 0.569 0.417

3553 4137 584 0.130 0.741 0.741

3553 4139 586 -0.058 -0.560 -0.277

3553 4248 695 0.130 0.741 0.741

3553 4283 730 -0.021 -1.000 -0.171

3553 4400 847 0.070 1.000 0.583

3605 3806 201 -0.050 -1.000 -0.300

3605 3859 254 -0.099 -1.000 -0.463

3605 3935 330 -0.083 -1.000 -0.410

3605 4121 516 -0.083 -1.000 -0.410

3605 4129 524 -0.050 -1.000 -0.300

3605 4137 532 -0.083 -1.000 -0.410

3605 4139 534 0.198 1.000 0.828

3605 4248 643 -0.083 -1.000 -0.410

3605 4283 678 -0.033 -1.000 -0.239

3605 4400 795 -0.033 -1.000 -0.239

3806 3859 53 0.099 1.000 0.649

3806 3935 129 0.060 0.569 0.417

3806 4121 315 0.060 0.569 0.417

3806 4129 323 0.072 0.614 0.614

3806 4137 331 0.060 0.569 0.417

3806 4139 333 -0.017 -0.267 -0.097

3806 4248 442 0.060 0.569 0.417

3806 4283 477 -0.012 -1.000 -0.126

3806 4400 594 -0.012 -1.000 -0.126

3859 3935 76 0.165 1.000 0.886

3859 4121 262 0.165 1.000 0.886

3859 4129 270 0.099 1.000 0.649

3859 4137 278 0.165 1.000 0.886

3859 4139 280 -0.079 -0.633 -0.354

3859 4248 389 0.165 1.000 0.886

3859 4283 424 -0.025 -1.000 -0.194

3859 4400 541 0.066 1.000 0.516

3935 4121 186 0.176 1.000 1.000

3935 4129 194 0.105 1.000 0.733

3935 4137 202 0.176 1.000 1.000

3935 4139 204 -0.103 -1.000 -0.495

3935 4248 313 0.176 1.000 1.000

3935 4283 348 -0.021 -1.000 -0.171

3935 4400 465 0.070 1.000 0.583

4121 4129 8 0.105 1.000 0.733

4121 4137 16 0.176 1.000 1.000

4121 4139 18 -0.103 -1.000 -0.495

4121 4248 127 0.176 1.000 1.000

4121 4283 162 -0.021 -1.000 -0.171

4121 4400 279 0.070 1.000 0.583

4129 4137 8 0.105 1.000 0.733

4129 4139 10 -0.062 -1.000 -0.363

4129 4248 119 0.105 1.000 0.733

4129 4283 154 -0.012 -1.000 -0.126

4129 4400 271 -0.012 -1.000 -0.126

4137 4139 2 -0.103 -1.000 -0.495

4137 4248 111 0.176 1.000 1.000

4137 4283 146 -0.021 -1.000 -0.171

4137 4400 263 0.070 1.000 0.583

4139 4248 109 -0.103 -1.000 -0.495

4139 4283 144 -0.041 -1.000 -0.289

4139 4400 261 -0.041 -1.000 -0.289

4248 4283 35 -0.021 -1.000 -0.171

4248 4400 152 0.070 1.000 0.583

4283 4400 117 -0.008 -1.000 -0.100
