# Supplementary material for: Analysis on single nucleotide polymorphisms of the PeTPS-(-)Apin gene in Pinus elliottii
Source: PLoS One. 2022 May 27;17(5):e0266503. doi: 10.1371/journal.pone.0266503 (PMC9140247; doi:10.1371/journal.pone.0266503)
Supplement: S1 Schedule — (DOCX) [file pone.0266503.s010.docx]

**S1 Schedule. Variation in 29 resin traits of 110 slash pines.**

| **Resin Character** | **Mean** | **Maximum** | **Minimum** | **Standard Deviation** | **Variable Coefficient** |
| --- | --- | --- | --- | --- | --- |
| **Basic resin-producing-capacity *W_0_* (kg)** | 0.12 | 0.21 | 0.05 | 0.02 | 16.67% |
| **Potential resin-producing-capacity *W_P_* (kg)** | 6.63 | 12.10 | 2.45 | 1.24 | 18.70% |
| **Turpentine oil (%)** | 43.75 | 120.43 | 11.68 | 16.77 | 38.33% |
| **α-Pinene (%)** | 20.61 | 53.89 | 8.64 | 7.52 | 28.92% |
| **Camphene (%)** | 0.36 | 0.73 | 0.12 | 0.09 | 25.00% |
| **β-Pinene (%)** | 14.43 | 37.46 | 2.59 | 6.20 | 42.97% |
| **Dipentene (%)** | 0.37 | 2.22 | 0 | 0.26 | 70.27% |
| **Cymene (%)** | 0.04 | 0.32 | 0 | 0.07 | 175.00% |
| **Myrcene (%)** | 2.24 | 25.71 | 0.05 | 2.66 | 118.75% |
| **Cycloisilongifolene (%)** | 0.35 | 0.42 | 0.28 | 0.04 | 11.43% |
| **Resin (%)** | 74.68 | 215.38 | 42.73 | 63.32 | 84.79% |
| **Pimanthrene (%)** | 2.23 | 15.08 | 0.07 | 5.67 | 254.26% |
| **Pimarinal (%)** | 0.27 | 1.91 | 0.04 | 0.31 | 114.81% |
| **Pimaric acid (%)** | 2.48 | 15.5 | 0.16 | 5.74 | 231.45% |
| **Elliotinoic acid (%)** | 2.49 | 15.86 | 0.11 | 5.90 | 236.95% |
| **Sandaracopimaric acid (%)** | 0.22 | 0.81 | 0.04 | 0.10 | 45.45% |
| **Dehydroabietic aldehyde (%)** | 0.26 | 0.56 | 0.02 | 0.12 | 46.15% |
| **Isopimaric acid (%)** | 5.77 | 16.14 | 2.68 | 4.65 | 80.59% |
| **Levopimaric acid (%)** | 4.43 | 6.41 | 2.06 | 0.73 | 16.48% |
| **Palustric acid (%)** | 19.43 | 22.88 | 17.01 | 2.25 | 11.58% |
| **6,8,11,13-Abietatetraenoic acid (%)** | 2.65 | 17.14 | 0 | 6.40 | 241.51% |
| **Dehydroabietic acid (%)** | 5.90 | 17.44 | 2.93 | 5.17 | 87.63% |
| **Abietic acid (%)** | 12.75 | 18.24 | 10.37 | 2.65 | 20.78% |
| **Neoabietic acid (%)** | 11.41 | 19.41 | 8.77 | 3.66 | 32.08% |
| **Mercusic acid (%)** | 0.28 | 0.70 | 0.06 | 0.14 | 50.00% |
| **7,13,15-Abietatrienoic acid (%)** | 3.46 | 20.55 | 0.10 | 7.55 | 218.21% |
| **8,14-Dihydro pimaric acid (%)** | 0.29 | 1.18 | 0.04 | 0.21 | 72.41% |
| **15-Hydroxyl hydrogen abietic acid (%)** | 3.75 | 21.89 | 0.40 | 8.00 | 213.33% |
| **7-Hydroxyl hydrogen abietic acid (%)** | 0.49 | 2.22 | 0 | 0.42 | 85.71% |
