# Supplementary material for: Analysis on single nucleotide polymorphisms of the PeTPS-(-)Apin gene in Pinus elliottii
Source: PLoS One. 2022 May 27;17(5):e0266503. doi: 10.1371/journal.pone.0266503 (PMC9140247; doi:10.1371/journal.pone.0266503)
Supplement: S2 Schedule — (DOCX) [file pone.0266503.s011.docx]

**S2 Schedule. Overview of 12 plus trees with high α-pinene content.**

| **Name** | **Genotype** | **Subgroup** | ***W_0_*** | ***W_P_*** | **Turpentine** | **α-Pinene** | **β-Pinene** |
| --- | --- | --- | --- | --- | --- | --- | --- |
| **2Ⅰ②** | AABBCC | Green | 0.10 | 9.94 | 72.73 | 53.89 | 14.14 |
| **70Ⅰ②** | AABBCC | Red | 0.11 | 9.00 | 38.31 | 35.97 | 1.71 |
| **72Ⅰ③** | AABBCc | Blue | 0.08 | 9.00 | 48.79 | 30.37 | 25.75 |
| **4Ⅰ②** | AABBCc | Blue | 0.16 | 11.94 | 71.43 | 44.97 | 16.68 |
| **93Ⅰ②** | AABBCc | Blue | 0.09 | 8.75 | 34.07 | 27.18 | 5.85 |
| **81Ⅰ③** | AABBCc | Green | 0.11 | 10.50 | 33.29 | 25.86 | 6.86 |
| **27Ⅰ④** | AABBCc | Green | 0.18 | 16.25 | 31.360 | 24.63 | 4.96 |
| **101Ⅰ④** | AABBCc | Blue | 0.19 | 12.00 | 31.57 | 24.23 | 5.35 |
| **38Ⅰ④** | AABBCc | Blue | 0.15 | 12.88 | 34.84 | 23.19 | 10.06 |
| **100Ⅱ①** | AABBCc | Red | 0.12 | 9.13 | 29.28 | 22.51 | 5.02 |
| **48Ⅰ②** | AABBCc | Blue | 0.13 | 8.73 | 29.09 | 22.22 | 6.51 |
| **87Ⅰ②** | AABBCc | Blue | 0.12 | 9.50 | 28.79 | 22.04 | 6.50 |
| **Mean** |  |  | 0.13 | 10.63 | 40.30 | 29.76 | 9.11 |
| **Control** |  |  | 0.12 | 6.63 | 43.75 | 20.61 | 8.70 |

Mean refers to the average of traits of 12 plus trees, and control refers to the average of 110 samples. Subgroup was based on SSR group structure analysis results of 110 *P. elliottii*, and the method and results were described in support information S5. The method of *W_0_*, *W_P_*, turpentine, α-pinene, and β-pinene content were described in support information S3, S4.
