# Supplementary material for: Analysis on single nucleotide polymorphisms of the PeTPS-(-)Apin gene in Pinus elliottii
Source: PLoS One. 2022 May 27;17(5):e0266503. doi: 10.1371/journal.pone.0266503 (PMC9140247; doi:10.1371/journal.pone.0266503)
Supplement: Attachment — Submitted filename: Response to Reviewers .docx [file pone.0266503.s012.docx]

Reviewer #1: In this study the authors aimed to identify molecular markers associated with 29 resin traits in Pinus elliotii, for their possible later use in marker-assisted selection. They use 110 trees and 120 SSRs to assess population structure, and then identify 72 SNPs in gene PeTPS-(-)Apin, related to rosin quality. They find evidence of 10 SNPs in this gene significantly associated with 7 resin traits, and suggest using the target gene for plus tree selection within a species’ breeding program.

Overall, this manuscript tackles an interesting topic by bringing together genetic and phenotypic data, for further tree breeding purposes. However, the manuscript is not properly structured and I think that several limitations of the analysis may compromise the findings. In its current state and in light of my major comments below, I do not think this manuscript is suitable for publication in PLOS ONE. However, if these issues below were to be comprehensively addressed, I would be happy to review a new version of the document.

Response: We really appreciate your suggestions that would improve the manuscript. Thus, we have revised the manuscript according to your comments.

Major comments:

1. The papers needs to be streamlined, and will benefit from rigorous scientific editing. While the english language is largely correct, the poor structure of the manuscript (intro, m&m, results, discussion, conclusions) makes it difficult to read and understand. Many unimportant details are provided along the text, while the most important ideas and hypothesis to be tested are not so much highlighted.

Response:I have modified part of the structure and language according to your requirements, especially the discussion section.

2. Regarding the methods, considering that this is a paper focused on breeding, I have missed important basic data, like for example heritability. Also genetic correlations among resin components. Are there correlations or trade-offs between the studied resin traits? I think it could be helpful to look for pairwise correlations between these traits. A classic breeding approach, including quantitative genetic data, would complement the understanding of the document, provided that the data are already available.

Response:Thanks for your suggestions. In terms of experimental materials, 110 trees from different families were selected for association analysis at the molecular level. Based on the comments, the main purpose of this paper is genetic improvement and breeding. The basic data of heritability and genetic correlation are important prerequisites for this paper to be carried out, and these data need more experimental materials than this paper. Our research group studied on these basic data before, and some of the data you mentined have been published in Chinese core journals. For the integrity of this paper, the research methods and analysis of these data are not included in the text, but the main data and references are added in the revised manuscript to ensure the data reliability of this paper. (L423-L425)

3. From the described methods, I have not found a justification for all the steps. Starting from the genetic structure analysis. A great number of SSRs (120) is used to assess population structure. But, how are the results taken into account in the discussion? For example: from the final 12 selected trees, do they come from different genetic pools? What can be done to widen the genetic basis of the breeding population? These are important questions regarding the sustainability of a breeding program.

Response:Due to the huge genome of *Pinus ellidtii*, only EST-SSR can be used to evaluate the population structure with limited information. Although there may be some errors between the experimental results and the real situation, I used 120 pairs of primers, hoping to reduce the errors by increasing the number of tests. Of these, the results of population structure can only be used to obtain the data required for association analysis in this experiment However, it neither represent the population structure of the whole *Pinus ellidata* population, nor be used to study the origin and geographical differences.

However, I have added the information of group structure in Schedule I (original Table 10) according to the comments, and supplemented it with text analysis (L445-446).

4. However, my major concern is about the statistical methods used for association analysis. A said by authors, they cannot discard false positives because of the small population size, population structure and a reliable genetic distance matrix (L328-330). LD between markers and false positives due to kinship relations and population structure are indeed key issues to be solved in association analysis. First of all, I am concerned that the number (59) and distribution of SNPs used in this study may bias the results. The authors recognize that there is actually a high level of LD and close linkage between markers (L262-263, L278). This could certainly affect the results of the association test, increasing the number of associated markers. The number of trees (110) is also quite small for association testing. Regarding kinship, how where the genotyped trees selected within families? The K matrix was calculated using SSRs or SNPs? If that is the case, I would not rely too much on a K matrix calculated with only 59 SNPs. Regarding population structure, admixture coefficients would improve if averaged across replicated runs (using for example software CLUMPP). Moreover, after association analysis, multiple testing correction is also needed to control for false positives when a large number of traits are tested. One option could be using the false discovery rate (FDR) method (Storey and Tibshirani 2003). Overall, I have reasonable doubts that the associations described as significant are really significant, or could rather be false positives.

Response:K matrix and Q matrix were SSR markers.

Due to limited funds and backward study of tree species. This paper carried out association analysis of candidate genes, and the whole genome was unknown. Thus, there were only 59 SNPs with a frequency of 1/78.61bp. I would not rely K and Q matrix calculated with only 59 SNPs, so I selected another 120 pairs of SSR primers from the gene banks for the calculation of K and Q matrices. Moreover, this data is only for subsequent association analysis which would be accurately generated by MLM model rather than GLM.

I know that many association analyses based on a large number of SNPs (GWAS, etc.) use SNP data to calculate K and Q matrices, because the data is large enough and accurate enough. However, in association analysis of candidate genes, GLM model or simple correlation is generally used and performed. Using SSR markers to calculate K and Q matrices.And then, doing association analysis with SNP markers.We agree with your suggestions. Actually, the research group is also continuing to carry out other verification with the replenishment of funds and experimental materials in the later stage,.

I have therefore added these risks in the article (L344-347).

Minor comments:

- Introduction: I found excess literature about non-relevant information for the scope of the paper, such as information about resin production (L38-43, L50-52), while lacking key information about the genetic control of resin traits.

Response:I have deleted the production information according to your comments and added the genetic information of resin properties.

-Overall, the manuscript lacks bibliographic references. Examples: L58, 70, 83, 90, 258. I would encourage authors to upgrade their references with publications of high impact international journals.

Response:I have added references of international journals according to your suggestions.

-Materials and methods: regarding plant materials, it is not clear how the 110 trees genotyped and phenotyped for resin traits where selected from the 110 families of the progeny trials. In population structure analysis, I would clarify how the 0,1 format data was transformed to round numbers, corresponding to a bp position.

Response:How the 110 trees are selected to supplement in L121-124.

Data conversion methods for population structure analysis are supplemented in L136-137

-L336-338: Why are SNPs in HWE discarded for further analysis and breeding pourposes?

Response:I have explained this problem in L353-355. I believe that even SNP sites that do not conform to HWE may be associated with the target trait, but this will increase the false positives in association analysis. In the case that the population in this paper is not particularly large, I chose to discard them. However, I will not give up these valuable sites. If I can find a suitable verification group in the future, I will choose SNP sites with high reproducible to continue my research.

-Discussion: a first short paragraph with main findings would be appreciated.
Response:I have added the main findings in L325-329 according to your comments

- Figure 1 should preferably belong to supplementary material.

Response:I have taken Figure 1 as the supplementary material according to your opinion.

- Figures 2 an 3 are redundant. They show the same information, in different scale. It would be useful to see how are progeny trial populations or families distributed in the x-axis.

Response:I have deleted Figure 2 according to your request.

- Suggestion for associated SNP data figure: Instead of table, a boxplot for each SNP marker, showing median and interquantile range of phenotype variation would be more visual and informative for the reader.

Response:Table 9 has been deleted. Figures 10 and 11 have been added. Table 10 is schedule.

Reviewer #2: Manuscript may be considered after addressing the following comments, importantly point 2.

Response:We are so delighted to have your comments. Thanks for the suggestions. We have revised the manuscript according to the comments.

1. Provide details and references fr the methods used such as GC and MS procedures.

Response:The GC and MS programs were developed by our research group in the laboratory for many years.

I have supplemented the references for the use of GC and MS for the composition of turpentine according .L185

2. Attach a picture of cultivated plants (field trial) and plots made along with distances between each plants on which genetic analysis was performed. This is the core of the study.

Response: I have added the photos of woodland according to your idea (Fig.1).

Because the area of the experimental forest land is too large. I added a schematic diagram of the forest land (Fig.2).

I also added the spacing of individual plants. L119-120

3. Verify the data in Table 3. example red group totals 16.

Response:I have checked it and revised it.

4. Provide a heading Figure legends.

Response:I have provided an example of the title diagram as you requested.

5. Some figures lacks legends.

Response:I have added the relevant data.

6. Figure 5 needs more details about intron and exons or put it in the legend.

Response:I have marked the details of exons and introns on the diagram according to your instructions, and added the legend. L265

7. Mention the details of structure parameters used. Ex. Burn-in period, MCMC etc.

Response:I have explained the parameter details as you suggested. L139-140
