# Supplementary material for: Analysis on single nucleotide polymorphisms of the PeTPS-(-)Apin gene in Pinus elliottii
Source: PLoS One. 2022 May 27;17(5):e0266503. doi: 10.1371/journal.pone.0266503 (PMC9140247; doi:10.1371/journal.pone.0266503)
Supplement: Attachment — Submitted filename: Response to Reviewers .docx [file pone.0266503.s013.docx]

Response Letter

I have received the modification suggestions of two experts, and the modification has been completed according to the expert suggestions. The reply to the expert suggestions is as follows:

Reviewer #1:

1. Overall the authors tried to address most comments, improving the manuscript. However, I still think that statistical analysis may compromise their findings, and thus their results and conclusions. I’m especially concerned about association testing results, as highlighted in major comment 4. Thus, I do not think this manuscript is suitable for publication in PLOS ONE in its present form. Response to authors for major comments review:

Response: We really appreciate your suggestions that would improve the manuscript. Thus, we have carefully revised the manuscript according to your comments.

2. Authors now provide a citation about heritability for turpentine and alfa-pinene in the species. My sense was that there is a need for trustable genetic control if then you are going to perform an association test, which consists in stablishing an statistic relationship between fenotypes (SNPs) and genotypes.

My interest in testing for genetic correlations between traits is to avoid false positives. If the studied traits are strongly correlated, we cannot say that the associated SNPs are responsible for variation in all those traits, indepently. We cannot ignore that they could rather be just associated with one of these traits, or them in all.

Response: Thank you again for your comments. As you said, if the traits are correlated, it is hard to say that the SNPs are responsible for variation in it. In fact, many studies have shown that there was a significant negative correlation between α-pinene and β-pinene content. That is why I use to different methods when we are choosing plus trees. Before I start this article, we used four genes, including *PeTPS-Bpin* (β-pinene synthetase gene), not just *PeTPS-(-)Apin* gene, but no SNPs associated with any of the other traits. Addtionally, candidate gene association analysis is not as accurate as the GWAS method, but the only advantage is to save the cost of the test. We believe that our research has some reference value even though there are shortcomings in technology. I added some correlation data and references in L441-442.

3. Despite the huge genome of pine tree species, I cannot agree that population structure can only be assessed through EST-SSR data. The genome is huge but highly repetitive. Population structure can be assessed using SNP data in selected genes or a higher number of SNPs distributed along the genome. However, population structure assessed with SSRs could be ok. But, if the computed population structure does not represent the structure of the studied population, as said by authors… How could it be used to correct for population structure in the mixed linear model implemented in the association analysis?

Response: Thank you again for your comments. As you said, SNP in selected genes or distributed along the genome can be used to evaluate population structure. However, only a limited number of specific genes have been published in *Pinus elliottii*, and there were few SNPs in the database. Conversely, there were a large number of SSR markers for *P. elliottii* itself, as well as general markers for other species of *Pinus*. The use of SSR markers is also aimed at greatly reducing our research costs. So we selected SSR markers after referring to studies on other *Pinus* such as Loblolly pine (*P. taeda*) (Eckert, 2010). Although this method would not perfectly perform an accurate result, it is a reasonable method and has certain reference value. I have made supplementary explanations in the text according to your suggestions (L136-137).

4. The statistical methods used for association analysis are still my major concern about the manuscript. I think the authors have not addressed what was highlighted about basic statistical problems which I think are at the base of their results and main findings. If you do not apply a false discovery rate correction method, neither know about genetic correlations between phenotypes in the studied traits, in my opinion, association analysis results are not reliable.

Response: Thank you very much for your advice. As you said, the results of our previous association analysis are not reliable. I have tested the results of my association analysis with The FDR method as suggested by you, and finally eliminated seven false positives, so that the phenomena I failed to explain before can be scientifically explained. The article has made a large number of modifications (L21-26, 193-195, 312-317, 327, 354-357, 431 ).

Reviewer #2:

All the comments have been address appropriately and manuscript may be accepted for publication. For future publications remember to provide comment by comment response in separate sheet (response sheet) not in the revised manuscript.

Response: Thank you again for your approval of this article.
