# Supplementary material for: Analysis on single nucleotide polymorphisms of the PeTPS-(-)Apin gene in Pinus elliottii
Source: PLoS One. 2022 May 27;17(5):e0266503. doi: 10.1371/journal.pone.0266503 (PMC9140247; doi:10.1371/journal.pone.0266503)
Supplement: Attachment — Submitted filename: Response to Reviewers.docx [file pone.0266503.s014.docx]

PONE-D-21-06793R2

Association of single nucleotide polymorphisms in the *PeTPS-(-)Apin* gene with resin traits in *Pinus elliottii*

PLOS ONE

**Dear Dr. Sharma:**

I’m delighted to receive your e-mail, and thank you very much for giving us an opportunity to revise our manuscript. We appreciate the editor and reviewers very much for their positive and constructive comments and suggestions on our manuscript entitled “Association of single nucleotide polymorphisms in the *PeTPS-(-)Apin* gene with resin traits in *Pinus elliottii*” (PONE-D-21-06793R2).

We revised it according to reviewers’ comments , and uploaded it according to your requirements. In terms of technology, we are grateful for the opinions of reviewers and very respectful to it. However, considering the particularity of the sample itself, we discussed this in the response letter. We only made slight modifications in the manuscript, and we also look forward to the understanding of the reviewers. In terms of data, we carefully checked the supporting information of all data, modified the incorrect format and reuploaded it, and supplemented the original data about Table 3. In terms of language, due to some language and grammatical errors mentioned by the reviewer, we require the Editage company to retouch the revised version and upload the certificate of editing (filename: Certificate_of_editing.docx).

If you have any questions, please feel free to contact us at the following address and email:

**Address:** Jiangxi Key Laboratory of Silviculture, 2011 Co-Innovation Center of Jiangxi Typical Trees Cultivation and Utilization, College of Forestry, Jiangxi Agricultural University, Nanchang 330045, China

**E-mail：**[zhanglu856@mail.jxau.edu.cn](mailto:zhanglu856@mail.jxau.edu.cn)

Thank you very much for your consideration of this manuscript.

We look forward to your kind response.

Sincerely,

Lu Zhang

**Response to comments and suggestions of reviewers:**

**1. If the authors have adequately addressed your comments raised in a previous round of review and you feel that this manuscript is now acceptable for publication, you may indicate that here to bypass the “Comments to the Author” section, enter your conflict of interest statement in the “Confidential to Editor” section, and submit your "Accept" recommendation.**

**Reviewer #3: (No Response)**

**2. Is the manuscript technically sound, and do the data support the conclusions?

The manuscript must describe a technically sound piece of scientific research with data that supports the conclusions. Experiments must have been conducted rigorously, with appropriate controls, replication, and sample sizes. The conclusions must be drawn appropriately based on the data presented.**

**Reviewer #3: No**

Response: In this manuscript, 29 resin traits were determined by methods of bark stress wounding and GC-MS, and the sampling and experiment were repeated to ensure accurate and authentic data. Structure V2.3.3 software was used to analyze population structure and genetic relationship, MLM program of TASSEL software was used for SNP association analysis, and FDR program was used to adjust P values. Finally, three SNPs of *PeTPS-(-)Apin* gene were found to be significantly correlated with α-pinene content, and realistic gain of 118.0 % of α-pinene content was obtained. The research of this manuscript has a certain reference value.

**3. Has the statistical analysis been performed appropriately and rigorously?**

**Reviewer #3: Yes**

Response: Thank you very much for your recognition of the statistical analysis method in this manuscript.

**4. Have the authors made all data underlying the findings in their manuscript fully available?

The** [**PLOS Data policy**](http://www.plosone.org/static/policies.action#sharing) **requires authors to make all data underlying the findings described in their manuscript fully available without restriction, with rare exception (please refer to the Data Availability Statement in the manuscript PDF file). The data should be provided as part of the manuscript or its supporting information, or deposited to a public repository. For example, in addition to summary statistics, the data points behind means, medians and variance measures should be available. If there are restrictions on publicly sharing data—e.g. participant privacy or use of data from a third party—those must be specified.**

**Reviewer #3: No**

Response: We have provided woodland photos at 3072×1728 and 3456×4608 resolutions in the support information, along with all raw data summaries and software analysis results. After your reminder, we have checked the uploaded support information. We uploaded the support information S5 as a supplement, and modified the incorrect format of S9, to ensure compliance with the PLOS data policy.

**5. Is the manuscript presented in an intelligible fashion and written in standard English?

PLOS ONE does not copyedit accepted manuscripts, so the language in submitted articles must be clear, correct, and unambiguous. Any typographical or grammatical errors should be corrected at revision, so please note any specific errors here.**

**Reviewer #3: No**

Response: The manuscript was edited by Editage. Under your reminding, we checked the manuscript and corrected some mistakes. We returned the manuscript to the Editage's office, re-edited it in English, and uploaded the English editor certificate (filename: Certificate_of_editing.docx) through the submission channel.

**6. Review Comments to the Author

Please use the space provided to explain your answers to the questions above. You may also include additional comments for the author, including concerns about dual publication, research ethics, or publication ethics. (Please upload your review as an attachment if it exceeds 20,000 characters)**

**Reviewer #3: The work's goal is good, and the authors put forth a lot of effort to convey the findings.
The customized association analysis methodologies, on the other hand, are unreliable.
I don't believe this manuscript is ready for publishing.
There are numerous errors, some of which are as follows:**

**(1) Correct the spelling ‘TASSLE’ to TASSEL at line 21, 22. Similarly on line 186. Go through the manuscript.
Change ‘rosin’ to resin line 17, 172**

Response: Thanks for your reminding, and I'm sorry to let you see these careless error. I have checked carefully the whole manuscripts, and corrected these mistakes. In addition, I also corrected other language and grammatical errors through the Editage company.

(1) Lines 21, 198.—“TASSLE” corrected to “TASSEL”.

(2) Lines 16, 17, 35, 37, 94, 173, 306, 306(2), 308, 312, 407, 452 and Table 5 (13 errors in total).—“rosin” corrected to “resin”.

**(2) Spell out ‘RY’ when you** **use it first time in the manuscript it is better.**

Response: Thanks for your reminding, and I'm sorry to let you see this careless error. I checked the whole manuscript carefully and corrected all of similar errors.

(1) Lines 157, 168.—“RY”, is not a professional noun abbreviation, and it only appears twice in the manuscript, both corrected to “resin yield”.

(2) Lines 75.— “TPS”, is used for the first time in the manuscript, and corrected to “Terpene synthase (TPS)”.

(3) Lines 97.—“MAS”, is used for the first time in the manuscript, and corrected to “marker-assisted selection (MAS)”.

(4) Lines 382.—“FHA”, is used for the first time in the manuscript, and corrected to “forkhead-associated (FHA)”.

**(3) Statements don’t match about the population structure: You write sub-population (K) K1 to K9 in Method section while in** **K = 2 to 7 is mention in result part.**

Response: Thanks for your reminding, and it's my carelessness. I checked the whole manuscript carefully and corrected all of similar errors.

(1) Lines 134.—The K value in the result section is correct, “set K to 1~9” in the material method part is corrected to “K set to 2~7”.

(2) Lines 133.—There is a parameter value error, and modified “100,000” to correct value “10,000”.

(3) Lines 223.—There is a numerical value error, and modified “37” to the correct value “35”.

**(4) The method of ∆K was not mention in method section**
**The value of MCMC reps is also missing
Method is not clear at line 183 – 187 (GC procedure and GC-MS procedure)**

Response: Thanks for your comments. I have added the following contents:

(1) Lines 134-137.—added the method of optimal K and ΔK value.

“The optimal value of K was determined using the ΔK method with the online program (Structure Harvester: http://taylor0.biology.ucla.edu/struct_harvest/).”

(2) Lines 133.—added the parameter of "Number of MCMC Reps after Burnin".

“"Number of MCMC Reps after Burnin" set to 10,000”.

(3) Lines 183-186.—supplemented the programs of GC and GCMS.

“GC was carried out using the following parameters: 60 ℃ for 2 min, 5 ℃·min-1 to 80 ℃, 30 ℃·min-1 to 230 ℃, finally 5℃·min-1 to 260 ℃, for 10 min. Injection volume: 0.4 μL. GC-MS was carried out using the following parameters: 60 ℃ for 2 min, 5 ℃·min-1 to 80 ℃, 30 ℃·min-1 to 230 ℃, finally 5℃·min-1 to 260 ℃, for 10 min. Injection volume: 0.1 μL.”.

**(5) Line 188-189: It is structure based association analysis. My main concern is that how could SSR based Q value be used in the MLM model of association mapping which is based on SNPs data. It is difficult to compare population structure data of different molecular marker. You should do structure analysis and generate Q value for SNPs data also. The association analysis data is not reliable.
The used K matrix is based on SSRs data or SNPs data? If this is based on SSRs data again I shocked how you could use this in SNPs based association mapping. You should use SNPs based K matrix as well.**

Response: Thanks very much for your comments. As you said, it is difficult to compare data of different molecular markers. And we also would like to be able to use more SNP markers distributed along the genome to obtain population structure data. However, it is a pity that this manuscript did not get more SNP markers.

TPS genes were cloned, sequenced, and found SNP loci, and performed a simple correlation analysis (ANOVA) between these SNPs and phenotypic traits. There was a significant association between *PeTPS-(-)Apin* gene and resin traits. In order to find the association loci suitable for MAS, we tried to add two fixed effects, K-matrix and Q-matrix, into the independent variables of association analysis. However, we obtained only 59 SNPs with an average distance of 78.61 bp, which is difficult to be used for population structure analysis. What is more, it is difficult to obtain more SNPs from the genome in the short term in the case of the *P. elliottii* genome-wide unknown.

SSR is a broad-spectrum marker distributed throughout the genome and widely used in population structure research. Based on the related research of *P. taeda*. (Eckert, 2010: <https://doi.org/10.1534/genetics.114.164087>), we tried to use SSR markers to obtain K matrix and Q matrix. I think the manuscript still has some reference value, even though this method may be not the best.

The discussion section of the manuscript talked about the issues, but it wasn't comprehensive enough. I made some improvements.

Line 347-353.— The original: “We still cannot rule out the possibility of false positives in the association analysis because of the K matrix and Q matrix were calculated using SSR markers, since the available SNP markers in the association analysis of candidate genes could did not cover the whole genome. Nevertheless, some information can be obtained from the limited data. ”

The revised: “Based on a small data volume of nucleotides database (less than 300 ), our existing SNP data cannot cover the genome-wide. Moreover, it is difficult to obtain more SNP data in the short term due to the enormous genome (>20,000 Mbp). Therefore, the K-matrix and q-matrix in association analysis are calculated using SSR markers, which may be controversial. However, some information can be obtained from the limited data.”

**(6) The total no. of sample used in this study is 110 but the sum of all samples from four groups comes 109 in Table 3 line 221**

Response: Thanks very much for your reminding. There were 110 samples in the manuscript. But in subgroups grouping, we classified the sample with proportion of a single color exceeded 40 % (Q≥0.4) into the subpopulation of that color. However, the maximum Q value of sample No.59 was less than 0.4, which could not meet the grouping conditions. Therefore, there were only 109 samples in Table 3.

I supplemented the method of subgroups grouping and the description of related problems:

Line 220-222 : “When the proportion of a single color exceeded 40 % (Q≥0.4), we classified the individual into the subpopulation of that color, but the maximum Q value of sample No. 59 was less than 0.4, so no statistics were collected. ”

**(7) 236-237: Nucleotide diversity (0.00276) and watterson’s theta value (0.00259) is low might be due to the less sample size not moderate.**

Response: I agree with you and revise the manuscript accordingly.

Line 253-255: “The nucleotide polymorphism of π 0.00276 and θw 0.00259, were at a low level, probably because more polymorphisms were not detected from the small sample size in this study.”.
