# Supplementary material for: Analysis on single nucleotide polymorphisms of the PeTPS-(-)Apin gene in Pinus elliottii
Source: PLoS One. 2022 May 27;17(5):e0266503. doi: 10.1371/journal.pone.0266503 (PMC9140247; doi:10.1371/journal.pone.0266503)
Supplement: Attachment — Submitted filename: Response to Reviewers.docx [file pone.0266503.s015.docx]

PONE-D-21-06793R3

Association of single nucleotide polymorphisms in the *PeTPS-(-)Apin* gene with resin traits in *Pinus elliottii*

PLOS ONE

**Dear Dr. Sharma:**

I’m delighted to receive your e-mail, and thank you very much for giving us an opportunity to revise our manuscript. We appreciate the editor and reviewers very much for their positive and constructive comments and suggestions on our manuscript entitled “Association of single nucleotide polymorphisms in the *PeTPS-(-)Apin* gene with resin traits in *Pinus elliottii*” (PONE-D-21-06793R3). We revised it according to reviewers’ comments , and uploaded it according to your requirements.

We are grateful for the opinions of reviewers and very respectful to it. We changed the title of the manuscript to " Analysis on single nucleotide polymorphisms of the *PeTPS-(-)Apin* gene in *Pinus elliottii* ". We deleted the parts that were not approved from the manuscript according to the comments of the reviewers, and revised them accordingly. However, considering the particularity of this manuscript, we have still uploaded the deleted sections as supporting information.

If you have any questions, please feel free to contact us at the following address and email:

**Address:** Jiangxi Key Laboratory of Silviculture, 2011 Co-Innovation Center of Jiangxi Typical Trees Cultivation and Utilization, College of Forestry, Jiangxi Agricultural University, Nanchang 330045, China

**E-mail：**[zhanglu856@mail.jxau.edu.cn](mailto:zhanglu856@mail.jxau.edu.cn)

Thank you very much for your consideration of this manuscript.

We look forward to your kind response.

Sincerely,

Lu Zhang

**Response to comments and suggestions of reviewers:**

**1. If the authors have adequately addressed your comments raised in a previous round of review and you feel that this manuscript is now acceptable for publication, you may indicate that here to bypass the “Comments to the Author” section, enter your conflict of interest statement in the “Confidential to Editor” section, and submit your "Accept" recommendation.**

**Reviewer #3: (No Response)**

**2. Is the manuscript technically sound, and do the data support the conclusions?

The manuscript must describe a technically sound piece of scientific research with data that supports the conclusions. Experiments must have been conducted rigorously, with appropriate controls, replication, and sample sizes. The conclusions must be drawn appropriately based on the data presented.**

**Reviewer #3: Partly**

Response: Thanks for your recognition. We deleted the association data based on population structure in accordance with your requirement, and focused on SNP analysis of *PeTPS-(-)Apin* gene. Genotyping of the population based on three SNPs, and the plus trees with high α-pinene content was selected. Finally, the actual α-pinene content was 118.0%. The research of this paper has certain reference value.

**3. Has the statistical analysis been performed appropriately and rigorously?**

**Reviewer #3: Yes**

Response: Thank you very much for your recognition of the statistical analysis method in this manuscript.

**4. Have the authors made all data underlying the findings in their manuscript fully available?

The** [**PLOS Data policy**](http://www.plosone.org/static/policies.action#sharing) **requires authors to make all data underlying the findings described in their manuscript fully available without restriction, with rare exception (please refer to the Data Availability Statement in the manuscript PDF file). The data should be provided as part of the manuscript or its supporting information, or deposited to a public repository. For example, in addition to summary statistics, the data points behind means, medians and variance measures should be available. If there are restrictions on publicly sharing data—e.g. participant privacy or use of data from a third party—those must be specified.**

**Reviewer #3: Yes**

Response: Thank you very much for your recognition of the data underlying the findings in this manuscript.

**5. Is the manuscript presented in an intelligible fashion and written in standard English?

PLOS ONE does not copyedit accepted manuscripts, so the language in submitted articles must be clear, correct, and unambiguous. Any typographical or grammatical errors should be corrected at revision, so please note any specific errors here.**

**Reviewer #3: Yes**

Response: Thank you very much for your recognition.

**6. Review Comments to the Author

Please use the space provided to explain your answers to the questions above. You may also include additional comments for the author, including concerns about dual publication, research ethics, or publication ethics. (Please upload your review as an attachment if it exceeds 20,000 characters)**

**Reviewer #3: Dear Editor,**

**Most of the writing is now ok, and the authors’ provides substantial information. But again my main concern is about the association mapping approaches adapted by the authors in the manuscript. Authors used Q value of SSRs in SNP based association mapping. It is unacceptable as the population structure reduced the biasness of association by clustering of genotypes on the basis of polymorphic/unlinked marker irrespective of other variables. It is difficult to relate the location of SSRs and SNPs marker (linked or unlinked). In this paper there is a chance of highly wrong association. The genetic proportion value of admixture level will be totally different with different molecular marker if the nature of marker is different. If Q value of SSR is used in SNPs based association analysis it gives false association. The association analysis based on ANOVA is ok moreover the population structure /diversity analysis of P. elliotti based on SSR data is ok but Q matrix and K matrix generated through SSRs data cannot be used in SNPs based association analysis it can only be used when the physical location of the both markers is within the LD range. Have you compare the physical location of those SSRs to the SNPs? In my suggestion, population structure based association data should be remove from the manuscript and revised the manuscript accordingly.**

Response: Thank you very much for your comments and suggestions, and I agree with you.

According to your suggestion, I deleted the section of association analysis based on population structure. I changed the title to "Analysis on single nucleotide polymorphisms of the *PeTPS-(-)Apin* gene in *Pinus Elliottii*". The focus of this manuscript was changed to SNPs analysis of *PeTPS-(-)Apin* gene, avoiding highly wrong association. However, due to the particularity of the samples, I still retained the contents of phenotypic determination and population structure analysis in the form of supporting information.

Changes to the framework of the article were as follows:

(1) Lines 126,178, “Materials and methods” section — “Population structure analysis”, “Determination of resin production capacity”, “Determination of turpentine composition”, and “Association analysis” section was completely deleted. At the same time, added support information files S3 (Determination method for resin traits) and S5 (Group structure analysis method and results).

(2) Lines 128,139. “Materials and methods” section — The section “*PeTPS-(-)Apin* gene cloning and SNP site typing” was divided into two parts “*PeTPS-(-)Apin* gene cloning” and “SNP site analysis and typing”.

(3) Line 179. “Results” section — “Group structure and kinship” section was completely deleted. And added it to the support information files S5 (Text. Group structure analysis method and results).

(4) Lines 182,376. “Results” section — The section “*PeTPS-(-)Apin* gene sequence and SNP sites” was divided into two parts “*PeTPS-(-)Apin* gene sequence” and “Diversity analysis of SNPs”.

(5) Lines 371,389. “Results” section — The section “Linkage disequilibrium and haplotype block” was divided into two parts “Linkage disequilibrium” and “Haplotype block”.

(6) Line 409. “Results” section — Change the section “Resin traits and SNP association analysis” to “Genotyping of SNPs association with resin traits”.

Other corresponding modifications are as follows:

(1) Lines 22-24. “Abstract - *Methods*” section — Change “Bark stress wounding and GC-MS were used to determine 29 resin traits. Structure v2.3.3 software was used to analyze population structure and genetic relationships, DnaSP v4.0 software was used to evaluate genetic diversity, the MLM program of TASSEL was used for SNP association analysis, and false discovery rate (FDR) was used for adjusting the p value.”

to “*PeTPS-(-)Apin* gene was cloned by double primers (external and internal). DnaSP V4.0 software was used to evaluate genetic diversity and linkage disequilibrium. SHEsis was used for haplotype analysis. SPSS was used for ANOVA and χ2 test.”.

(2) Lines 98-100. “Introduction” section — Change “the *PeTPS-(-)Apin* gene was cloned, 29 resin traits were determined, and functional single nucleotide polymorphisms (SNPs) were screened by association analysis. The optimal selection scheme of *P. elliottii* with high α-pinene content was also considered.”

to “the *PeTPS-(-)Apin* gene was cloned, and its single nucleotide polymorphisms (SNPs) and linkage disequilibrium were analyzed. Functional SNPs were screened by ANOVA, 110 samples were typed, and the optimal selection scheme of P. elliottii with high α-pinene content was also considered.”.

(3) Lines 178-179. “Materials and methods” section — Added the sentence “SHEsis was used for haplotype analysis. ANOVA and χ2 were performed to evaluate if the results conformed to the Hardy-Weinberg equilibrium using SPSS software.”.

(4) Line 423. “Results - Genotyping of SNPs association with resin traits” section — The original Table 5 was deleted, and added it to Schedule 1.

(5) Line 433. “Results - Genotyping of SNPs association with resin traits” section — A new table 5 was added “Genotypes frequency and polymorphisms of 110 Pinus elliottii samples based on 3 SNPs”.

(6) Line 422. “Results - Genotyping of SNPs association with resin traits” section — Added the sentence “The determination methods of these resin traits were shown in supporting information (S3, S4).”.

(7) Lines 426-428. “Results - Genotyping of SNPs association with resin traits” section — Added the sentence “Genotyping and polymorphism analysis were performed on 110 samples of Pinus elliottii. The expected heterozygosity, had an average of 0.5365, and the observed heterozygosity, had an average of 0.5030 (Table 5).”.

(8) Lines 474-477. “Discussion” section — Change “Therefore, the K-matrix and q-matrix in association analysis are calculated using SSR markers, which may be controversial. Nevertheless, some information can be obtained from the limited data. Using candidate gene association analysis, we divided the PeTPS-(-)Apin gene sequence into six haplotypes (Fig 7), screened out 3 loci associated with α-pinene content from 59 SNPs, and performed polymorphism analysis (Table 7). The expected heterozygosity, had an average of 0.5365, and the observed heterozygosity, had an average of 0.5030. The linkage disequilibrium r2 values of the TagSNPs are listed in Table 8. There was a strong linkage between CG615 and AT641 in haplotype block (-)Apin-1 (r2=1). We found that the contribution rate of the PeTPS-(-)Apin gene to α-pinene content was 37.264 %; thus it was considered an important candidate gene for this trait.”

to “Therefore, simple associations (ANOVA) were used for association analysis, which may be controversial. Nevertheless, PeTPS-(-)Apin gene was considered as an important candidate gene for α -pinene content, and three TagSNPs (CG615, AT641 and AG3859) were associated with α -pinene content.”.

(9) Line 567. “Discussion” section — “Among the 12 trees, 2 (16.67%) were from the Red group, 3 (25%) are from the Green group, and 7 (58.33%) are from the Blue group.” was deleted.
